# Identification of Ti(salen) Complexes for Efficient Catalysis in Single‐Electron Steps by Cyclic Voltammetry

**DOI:** 10.1002/anie.202507673

**Published:** 2025-06-17

**Authors:** Niklas Schmickler, Sergei Gerber, Lennart Hanz, Stefan Grimme, Zheng‐Wang Qu, Inke Siewert, Andreas Gansäuer

**Affiliations:** ^1^ Kekulé‐Institut für Organische Chemie und Biochemie Universität Bonn Gerhard‐Domagk‐Straße 1 53121 Bonn Germany; ^2^ Institut für Anorganische Chemie Georg‐August‐Universität Göttingen Tammannstr. 4 37077 Göttingen Germany; ^3^ Mulliken Center for Theoretical Chemistry Universität Bonn Beringstraße 4 53115 Bonn Germany

**Keywords:** Arylation, Catalysts, Cyclic voltammetry, Radicals, Salen ligand

## Abstract

We describe the identification of an active Ti(salen) catalyst for the radical arylation of epoxides by a cyclic voltammetry study of mechanism‐based predictors, such as the redox potentials of the complexes and their E_q_C_r_‐equilibria, for the success of catalysis. Surprisingly, by far the most active catalyst features an uncommon tetrasubstituted ligand backbone, which renders chloride binding to the active Ti(III) species less favorable, thereby increasing catalyst activity due to improved substrate binding. Catalysis is most efficient in the “green” solvent ethyl acetate and can be initiated using base metals as well as electrochemical methods for the reduction of the Ti(salen)‐precatalyst. Compared to the commonly employed titanocene catalysis, the use of the newly developed Ti(salen) catalyst allows for the use of milder and more sustainable reactions conditions, a broader substrate scope, and facile modification of the catalyst's electronic and steric properties.

## Graphical abstract

**Catalyst screening without catalysis**. By combining cyclic voltammetry and DFT calculations we were able to identify a highly active catalyst for the radical arylation of epoxides without performing any reactions. The prevention of chloride binding that leads to catalyst inhibition was established as the key structural feature. Our best catalyst is applicable for a broad variety of substrates after electrochemical or chemical activation.

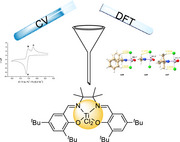

## Introduction

Catalysis is a central research area in chemistry. The advantages associated with its ecological as well as economical sustainability are shaping the landscape of chemical research to this day.^[^ ^1^, ^2^ ^]^ An often neglected but critical issue of sustainability in catalysis is the identification of superior catalysts and reaction conditions. Traditionally, this required extensive screening through expensive use of workforce, material input, and lab time. To avoid this, we recently proposed to identify mechanistic key aspects or so‐called predictors that allow a rational forecast of suitable catalysts and conditions before setting up any reaction.^[^ ^3^, ^4^, ^5^ ^]^

**Scheme 1:**
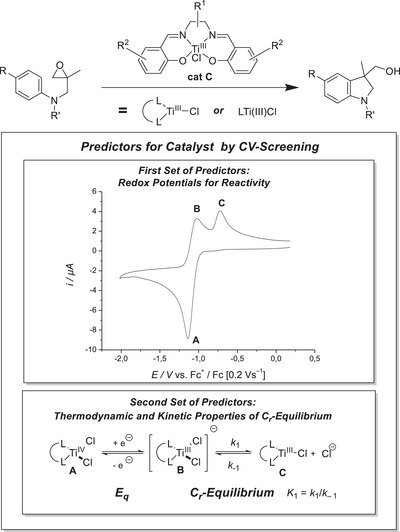
Predictor‐based CV‐screening approach to catalyst identification for the LTiCl_2_‐catalyzed radical arylation of epoxides.

To highlight our predictor concept, we describe our investigations into the identification of Ti(salen)Cl complexes as a new and efficient class of catalysts in the atom‐economical radical arylation reaction of epoxides (Scheme 1) by cyclic voltammetry (CV).^[^ ^6^ ^]^

To date, radical arylations of epoxides are carried out with titanocene(III) catalysts.^[^ ^6^, ^7^, ^8^, ^9^, ^10^, ^11^ ^]^

The broad applicability of these complexes suffers for two reasons: first, it is difficult to adjust their redox properties by variation of the cyclopentadienyl ligands’ substitution pattern because this requires noticeable synthetic effort.^[^ ^12^ ^]^ Second, after electrochemical reduction, titanocene complex **B** requires activation by halide abstraction through supramolecular H‐bond donors for efficient catalysis because halide loss from **B** is thermodynamically disfavored but required to form the catalytically active species **C**.^[^ ^3^, ^4^, ^5^, ^13^, ^14^, ^15^ ^]^ The quest for such efficient additives after the electrochemical reduction led to the CV screening approach that identified the features of the E_q_C_r_‐equilibrium as predictor for successful catalyst activation (Scheme 1, *E*_q_ denotes the quasi‐reversible electrochemical reduction of LTiCl_2_, and *C*_r_, the subsequent reversible chemical equilibrium reaction forming LTiCl). Further, our method also predicts that the substrate scope for the arylation reaction is related to the redox potential of the [(C_5_H_4_R)_2_TiX] catalysts. With this, we are able to circumvent performing catalytic reactions in the screening process but by rational design.^[^ ^3^, ^4^, ^5^ ^]^

To address the above mentioned short‐comings of the titanocene catalysts, we decided to study Ti(salen)Cl_2_ (LTi(IV)Cl_2_, Scheme 1) as catalysts for the radical arylation by our CV screening. They are potentially attractive targets as the ligands can be prepared easily in a large structural variety. They allow a modular variation of their steric and electronic properties by the substituents in the backbone (R^1^) and in the arenes (R^2^).^[^ ^16^, ^17^, ^18^, ^19^, ^20^, ^21^, ^22^, ^23^ ^]^

The use of the Ti(III)/Ti(IV) redox pair of Ti(salen) complexes for catalytic radical reactions is a relatively unexplored field of research. Examples include the Ti(salen)‐catalyzed pinacol coupling reported by Joshi^[^ ^24^ ^]^ and the formal Ti(salen)Cl‐catalyzed [3+2] cycloaddition of cyclopropyl ketones and alkenes developed by Lin. In cooperation, the Lin and Sigman groups have outlined factors affecting the enantioselectivity of this process.^[^ ^25^, ^26^ ^]^ More recently, Zhang reported a system for Ti‐catalyzed opening of methyl‐substituted glycidic epoxides with concomitant radical reduction or allylation, as well as kinetic resolutions by utilizing Ti(salen) complexes.^[^ ^27^, ^28^, ^29^, ^30^ ^]^

## Results and Discussion

### CV Screening of LTi(IV)Cl_2_ and Electrochemically Reduced LTi(IV)Cl_2_ as Catalysts in Radical Arylation

**Figure 1:**
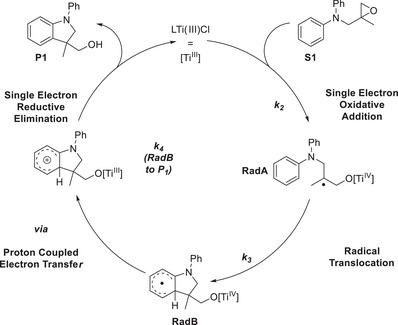
Catalytic cycle of the LTi(III)Cl‐catalyzed radical arylation of epoxides.^[^ ^6^ ^]^

The steps of the catalytic cycle of the radical arylation of epoxides (Figure 1) that critically depend on the redox‐properties of the LTi(III)Cl catalyst are the epoxide opening (the single‐electron oxidative addition, **S1** → **RadA**) and the rearomatization step (the single‐electron reductive elimination, **RadB** → **P1**).^[^ ^7^, ^31^, ^32^ ^]^ To assess the thermodynamic feasibility of these two steps, we employ the redox potentials of the Cp_2_Ti(III)Cl‐based systems as predictors because Cp_2_Ti(III)Cl constitutes a catalyst for the radical arylation. Thus, the Ti(salen) catalysts (LTi(IV)Cl_2_) should have reduction potentials in the range of −0.85 V (versus Fc^+|0^) to facilitate epoxide opening, similar to the value for the redox pair Cp_2_Ti(III)Cl|Cp_2_Ti(IV)Cl^+^ and about −1.27 V (versus Fc^+|0^) for rearomatization, which is the value for the redox pair Cp_2_Ti(IV)Cl_2_|Cp_2_Ti(III)Cl_2_^−^ obtained by Daasbjerg and coworkers.^[^ ^13^, ^14^, ^15^ ^]^ Those values are our first set of predictors.

The efficiency of catalysis will be affected by the relative concentration of the two Ti(III) species **B** and **C** (Scheme 1) that depend on the features of the C_r_‐part of the E_q_C_r_‐equilibrium.^[^ ^3^, ^4^, ^5^, ^33^ ^]^ The quantitative thermodynamic and kinetic data of the C_r_‐equilibrium of the Ti(salen)‐based LTi(III)Cl systems will constitute our second set of predictors.

**Figure 2:**
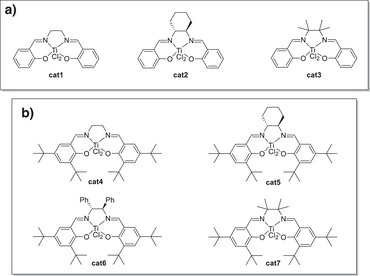
Library of LTi(IV)Cl_2_ complexes employed in the CV‐screening studies (a: ligands from salicylic aldehyde, b: arenes with *tert*‐butyl substituents). For the synthesis of the complexes, see Supporting Information.

In order to identify suitable catalysts for the radical arylation and to validate the applicability of our CV screening method, we synthesized a library of seven Ti(salen)Cl_2_ complexes (Figure 2a,b) to understand the arenes’ and the backbones’ substituent effects on our two predictors, the redox potentials of the LTi(IV)Cl_2_ systems, and the follow‐up C_r_‐equilibria.

All CV data were recorded in THF with [*n*Bu_4_N]PF_6_ as electrolyte, glassy carbon disk as working electrode, and referenced internally versus Fc^+|0^. All LTi(IV)Cl_2_‐complexes exhibit a reduction at a potential *E* ^0^ between −1.05 and −1.32 V. As expected, the *tert*‐butyl substituents at the aryl substituents lead to lower reduction potentials for the Ti^IV|III^ redox couple due to their electron‐donating character.

**Figure 3:**
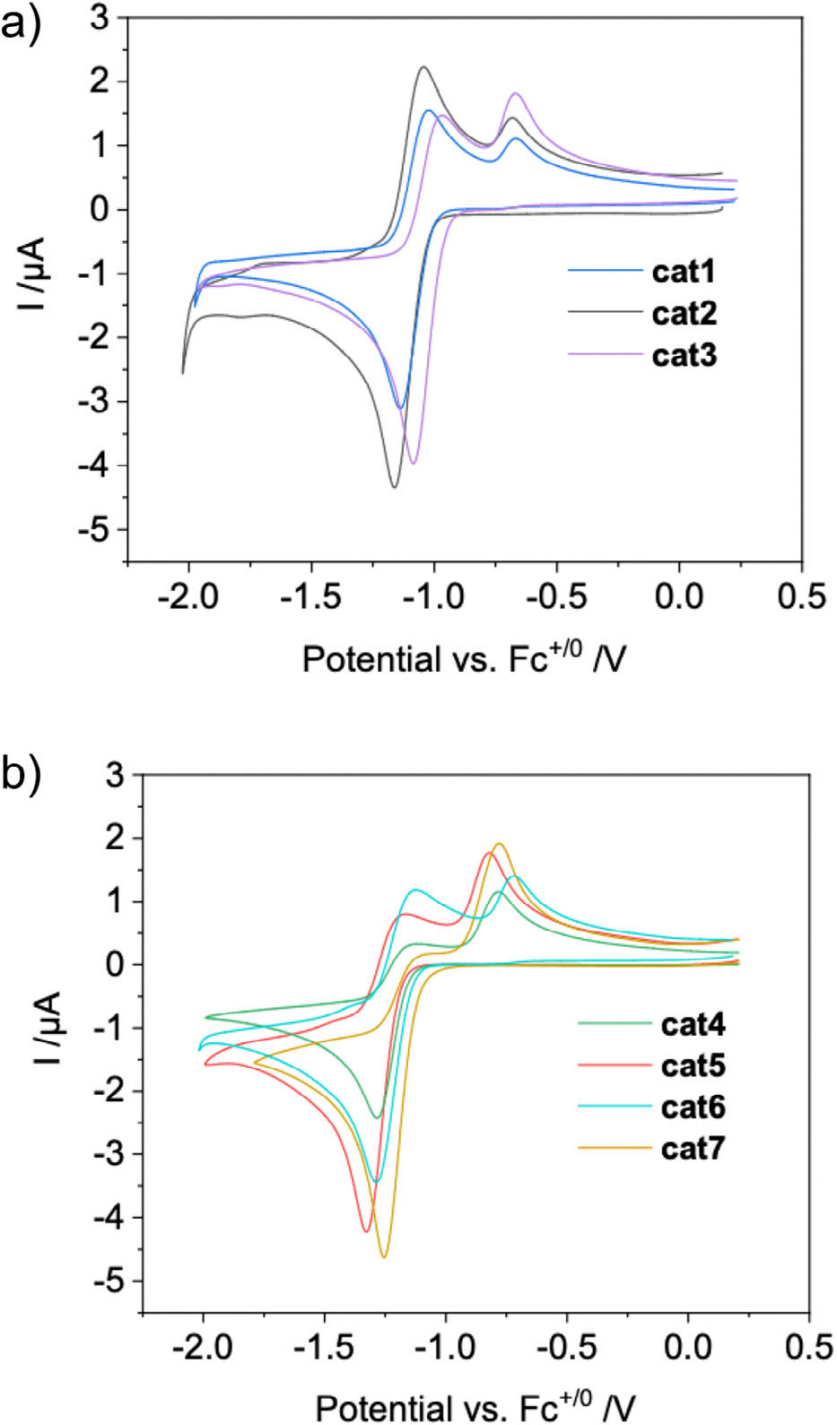
Cyclic voltammograms of a) **cat1**–**cat3** and b) **cat4**–**cat7** in THF, 0.1 M [nBu_4_N]PF_6_, *v* = 0.2 Vs^−1^.

All compounds exhibit two oxidations in the reverse scan, corresponding to the oxidation of LTi(III)Cl_2_^−^ and LTi(III)Cl, respectively. Most notably, for all salen complexes investigated, the wave pertaining to the oxidation of LTi(III)Cl is already visible at a scan rate of *ν* = 0.2 Vs^−1^. This is in sharp contrast to the CV data of Cp_2_Ti(IV)Cl_2_, where the oxidative wave of Cp_2_Ti(III)Cl is, if at all, barely visible at *ν* = 0.2 Vs^−1^ (Figure 3).^[^ ^3^, ^4^, ^5^, ^13^, ^14^, ^15^ ^]^

Scan rate normalized presentation of the CV data reveal that the ratios LTi(III)Cl_2_^−^|LTi(III)Cl decrease with increasing scan rates, which can only be rationalized by a fast equilibrium reaction for chloride loss after reduction as proposed previously for Cp_2_Ti(III)Cl_2_^−^ (Figures S3–S9). In order to quantify the kinetics and thermodynamics of the coupled electrochemical and chemical processes, the CV data were simulated using the DigiElch Software.^[^ ^34^ ^]^ A Butler‐Volmer model with fixed *α* = 0.5 was employed covering a large scan rate range from 0.05 to 10 Vs^−1^. Decent fits were obtained using the parameters shown in Tables 1 and S1. The results for Cp_2_Ti(IV)Cl_2_ in THF are taken from Daasbjerg's seminal study.^[^ ^13^, ^14^, ^15^ ^]^

**Table 1:** Quantitative kinetic and thermodynamic data of the E_q_C_r_‐equilibria of **cat1**–**cat7** obtained by a simulation of the CV data using the DigiElch Software.

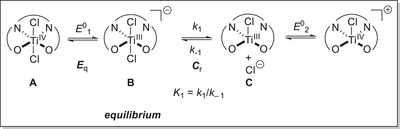					
L_2_TiCl_2_	*E* ^0^_1_ (V)^a)^	*E* ^0^_2_ (V)^a)^	*K*_1_ (M)	*k*_1_ (s^−1^)	*k_−_*_1_ (M^−1^s^−1^)
Cp_2_TiCl_2_	−1.27	−0.85	10^−4^	2 × 10^4^	2 × 10^8^
**cat1**	−1.10	−0.75	10^−3^	1.5 × 10^1^	1.5 × 10^4^
**cat2**	−1.13	−0.76	2 × 10^−3^	4 × 10^1^	5 × 10^3^
**cat3**	−1.05	−0.75	3 × 10^−3^	1.5 × 10^1^	2 × 10^4^
**cat4**	−1.27	−0.87	3 × 10^−2^	2.8 × 10^2^	9.2 × 10^3^
**cat5**	−1.32	−0.90	4.5 × 10^−2^	7 × 10^2^	1.6 × 10^4^
**cat6**	−1.29	−0.79	4.5 × 10^−2^	4 × 10^3^	8.9 × 10^4^
**cat7**	−1.23	−0.87	10^−1^	10^2^	10^3^

*Note*: values for Cp_2_TiCl_2_ taken from Refs. [13, 14, 15].

^a)^ Versus Fc^+|0^/V.

The oxidation potentials *E* ^0^_2_ of reduced **cat1**–**cat7** are close to the corresponding value of Cp_2_Ti(III)Cl, and thus, all complexes should be able to reductively open epoxides based on thermodynamic grounds. Moreover, they should also be able to act as oxidants in the rearomatization reaction of **RadB** via PCET (Figure 1) because their values of *E* ^0^_1_ (−1.05 to −1.32 V) are in the same range as those of Cp_2_Ti(IV)Cl_2_, taking into account that the exchange of Cl^−^ versus alkoxide has a similar impact on the redox potentials in all complexes (cf. the peak potential of the reductive wave of Cp_2_Ti(IV)(OEt)Cl (−1.21 V) is in the same range as that of Cp_2_Ti(IV)Cl_2_ (−1.27)).^[^ ^35^ ^]^

Assuming a free energy relationship, **cat1**–**cat3** should be particularly well‐suited for the rearomatization of **RadB**, the rate‐determining step of the arylation forming Cp_2_Ti(III)Cl, because their potentials *E* ^0^_1_ are the least negative ones and more positive than in Cp_2_Ti(IV)Cl_2_.

In order to rationalize the impact of the equilibrium on catalysis, we derived the rate law for product formation (Equation 1, see Supporting Information for its derivation)

(1) $$\;\frac{\partial \left[P\right]}{\partial t}=\frac{k_{2}K_{1}{\left[Ti^{\mathrm{tot}}\right]}_{0}\left[S\right]}{\left[Cl^{-}\right]+K_{1}+\frac{k_{2}K_{1}}{k_{3}}\left[S\right]+\frac{k_{2}K_{1}}{k_{4}}\left[S\right]}$$

where [Ti^tot^]_0_ is the initial concentration of the catalyst; *k*_1_ − *k*_4_: See Figure 1 and Scheme 1.

This can be further simplified assuming *k*_4_ ≪ *k*_2_ and *k*_3_.

(2) $$\frac{\partial \left[P\right]}{\partial t}=\frac{k_{2}K_{1}{\left[Ti^{\mathrm{tot}}\right]}_{0}\left[S\right]}{\left[Cl^{-}\right]+K_{1}+\frac{k_{2}K_{1}}{k_{4}}\left[S\right]}$$

Then the product formation depends on the reaction rates of the individual steps *k*_1_/*k*_−1_, *k*_2_, and *k*_4_. The reaction has a negative order in chloride ion concentration, that is, with decreasing concentration of chloride the reaction should get faster due to the pre‐equilibrium *K*_1_ of the catalytic active titanium species. Inspection of the reaction law reveals that equilibrium constant *K*_1_ should be large for fast turnover. Further, it implies that the formation *k*_1_ must be fast because *K*_1_ = *k*_1_/*k*_−1_, and that *k*_−1_ should be small as the reverse reaction *k*_−1_ competes with the productive ring opening reaction *k*_2_.

An analysis of the equilibrium constants *K*_1_ for chloride loss of the catalysts **cat1**–**cat7** shows that they are significantly larger for all catalysts by at least one order of magnitude compared to Cp_2_Ti(IV)Cl_2_. In turn, the crucial catalytically active species **C** would be the main species in 2 mM solutions of **cat1**–**cat7**, which is in contrast to solutions of 2 mM of Cp_2_Ti(III)Cl. The equilibrium constants *K*_1_ of the sterically more crowded *tert*‐butyl derivatives **cat4**–**cat7** are systematically higher than those of the nonsubstituted complexes **cat1**–**cat3**. The **cat7** with four methyl groups at the ethylene bridge of the ligand backbone has the highest value for *K*_1_ and the lowest value for *k−*_1_ and, thus, should be most suited for catalytic applications.

In order to understand the influence of the backbone substitution in the ligand on the C_r_‐equilibrium, quantum chemical calculations were conducted to provide structural data and energies for the chloride loss by reduced **cat4, cat6,** and **cat7**. These complexes are ideal candidates because they differ only in the degree of backbone substitution (**cat4**: unsubstituted, **cat6**: *trans*‐disubstituted, **cat7**: tetrasubstituted) and all contain the bis *tert*‐butyl substituted arenes. To this end, we carried out quantum chemical calculations at the PW6B95‐D3/def2‐TZVP + COSMO‐RS // TPSS‐D3/def2‐SVP + COSMO level of theory,^[^ ^36^, ^37^, ^38^, ^39^, ^40^, ^41^, ^42^, ^43^, ^44^, ^45^, ^46^, ^47^ ^]^ which has already been successfully applied in the investigation of elementary steps of reactions catalyzed and mediated by titanocene.^[^ ^48^, ^49^, ^50^ ^]^

**Figure 4:**
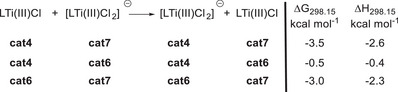
Free enthalpies and enthalpies (298.15 K in kcal mol^−1^) for the chloride exchange reactions between the neutral and anionic forms of **cat4**, **cat6**, and **cat7**.

The values of *ΔG*_298.15_ and *ΔH*_298.15_ for the chloride transfer between the anionic and neutral complexes are depicted in Figure 4 and the structural representations of **cat4, cat6,** and **cat7** in Figure 6.

**Figure 5:**
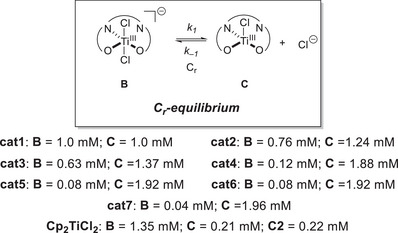
Equilibrium concentrations of the Ti(III) species in THF. In the case of Cp_2_Ti(IV)Cl_2_, **C2** refers to the dimer (Cp_2_Ti(III)Cl)_2_.

**Figure 6:**
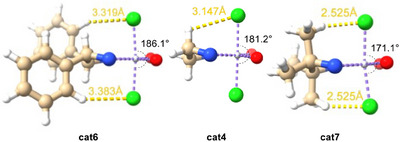
Effect of backbone substitution on the chloride binding by LTi(III)Cl as exemplified by the Cl–Ti–Cl angle of the chloride adduct LTi(III)Cl_2_^−^ for **cat4**, **cat6**, and **cat7**. Green, Cl; blue, N; red, O; colorless (bonded to Cl, N, and O), Ti; gold, C; colorless (ligand) backbone, H.

Our thermodynamic data clearly show that chloride loss to LTi(III)Cl is most advantageous for **cat7**. This is consistent with the equilibrium concentrations of the Ti(III) species (Figure 5) and shows that increasing steric bulk at the ligand backbone favors the formation of [LTi(III)Cl]. The structures of LTi(III)Cl_2_^−^ for **cat4**, **cat6**, and **cat7** reveal the reason for this behavior (Figure 6): The tetrasubstituted backbone of **cat7** results in repulsive interactions between the “pseudo”‐axial methyl groups and the chloride ligands as reflected by the Cl–Ti–Cl angles of LTi(III)Cl_2_^−^ (**cat6**: 186.1°; **cat4**: 181.2°; **cat7**: 171.1°) that deviate increasingly from 180° with substitution in the backbone.

We tested the hypothesis of **cat7** being most suited for the arylation reaction of **S1** by preparing electrochemically reduced solutions of **cat7**. As starting point, we used THF, the typically employed solvent in titanocene catalysis. As an alternative, ethyl acetate (EtOAc) was also tested because it has similar properties as THF, is generally considered as “greener”, and has been classified as “recommended” in a recent survey of solvent selection guides (Table 2).^[^ ^51^ ^]^

**Table 2:** Performance of 10 mol% electrochemically reduced **cat3**, **cat5**, **cat6**, and **cat7** in the radical.

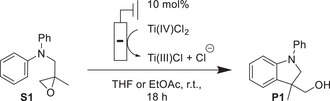			
Entry	Catalyst	Solvent	Conversion or isol. yield (%)
1	**cat7**	THF	27^a)^
2	**cat7**	EtOAc	79
3	**cat6**	EtOAc	18
4	**cat5**	EtOAc	13^a)^
5	**cat3**	EtOAc	17^a)^

^a)^ Conversion.

**Figure 7:**
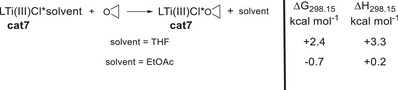
Free enthalpies and enthalpies (298.15 K in kcal mol^−1^) for the chloride exchange reactions between the neutral and anionic forms of **cat4**, **cat6**, and **cat7**.

At room temperature, THF resulted in a lower conversion (determined through measuring the **P1** to **S1** ratio by ^1^H NMR) than EtOAc that gave a complete conversion of **S1** and a good isolated yield of **P1** (79%). The **cat1**–**cat6** gave only disappointingly low conversions in both solvents. Our calculations (Figure 7) suggest that the reason for the superiority of EtOAc over THF as reaction solvent is the thermodynamically favored (*ΔG*_298.15_ and *ΔH*_298.15_) ligand exchange with the model substrate ethylene oxide.

Thus, our two sets of predictors, the redox potentials of **cat1**–**cat7** and the features of their E_q_C_r_‐equilibria, are crucial parameters to predict the superior catalysts.^[^ ^3^, ^4^, ^5^ ^]^ To establish the generality of our catalytic system, we explored the scope of the epoxide radical arylation (Table 3).

**Table 3:** Substrate scope for the radical arylation of epoxides utilizing 10 mol% electrochemically reduced **cat7** in EtOAc.

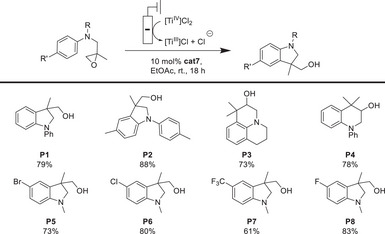

The yields of the desired products are good to high and electron‐withdrawing (**P5**–**P7**) and electron‐donating (**P2**) substituents at the arene are tolerated. The tricyclic product **P3** was also obtained in good yield.

Compared to electrochemically reduced Cp_2_Ti(IV)Cl_2_, the **cat7**‐based system developed here has the advantage of not requiring a supramolecular halide binder for shifting the C_r_‐equilibrium and also non‐activated epoxides are converted.^[^ ^3^, ^4^, ^5^, ^27^, ^28^, ^29^, ^30^ ^]^ Moreover, the arylation reactions can be performed in the “greener” solvent EtOAc^[^ ^51^ ^]^ and at room temperature. These points add to the overall sustainability of the electrochemically generated solutions of Ti(III) catalysts.

### Metal‐Reduced LTi(IV)Cl_2_: Shifting the C_r_‐equilibrium and Catalysis of the Radical Arylation

**Figure 8:**
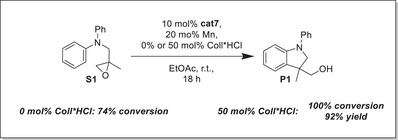
Performance of Mn‐reduced **cat7** in EtOAc with and without 50 mol% Coll*HCl (Coll = 2,4,6‐Me_3_‐pyridine).

An alternative to the electrochemical reduction of **cat7** is its reaction with a metal powder as chemical reductant. In titanocene catalysis, this is usually achieved with either Zn‐ or Mn‐dust.^[^ ^52^, ^53^ ^]^ During this reduction, MCl_2_ is formed, and, thereby, chloride is removed from the C_r_‐equilibrium. In this protocol, the formation of Cp_2_Ti(III)Cl_2_^−^ can be avoided and it may even be possible to access Cp_2_Ti(III)^+^ and [MCl_3_]^−^ or [MCl_4_]^2−^.^[^ ^3^, ^4^, ^5^, ^54^, ^55^ ^]^ As ZnCl_2_ is more Lewis acidic than MnCl_2_ the latter is usually preferred in titanocene catalysis. Hence, Mn‐dust was also employed in the chemical reduction of **cat7** in EtOAc (Figure 8).

Reduction of **cat7** by Mn dust was carried out either additive‐free or in the presence of 50 mol% Coll*HCl (Coll = 2,4,6‐Me_3_‐pyridine) because it has been shown in titanocene‐catalyzed radical arylations that this can result in a more stable catalyst.^[^ ^5^, ^33^ ^]^ In these cases, only MnCl_2_ and Cp_2_TiCl but no [Cp_2_TiCl_2_]^−^ are formed. It has been shown by CV that addition of Coll*HCl provides a resting state [Cp_2_TiCl_2_]^−^ for the catalytic system that prevents catalyst decomposition at higher conversion.^[^ ^5^, ^33^, ^56^ ^]^ We suggest that a similar mechanism is operating for the Ti(salen) complexes.

Indeed, the same effect was observed for **cat7** because conversion to **P1** was higher with Coll*HCl (74% versus 100%), and **P1** could be obtained in an excellent yield of 92%. However, the use of the metal and Coll*HCl results in less sustainable conditions.

We also tested the other catalysts and only **cat3** resulted in a high conversion to **P1** (84% yield, see Supporting Information for further details) at room temperature. Thus, the tetrasubstituted backbone is essential for efficient catalysis under the conditions of metal reduction and *tert*‐butyl substitution of the arenes increases this effect further.

The scope of the arylation with Mn‐reduced **cat7** (Table 4) is broad. Typically, good to high yields of the desired products were obtained.

**Table 4:** Substrate scope for the radical arylation of epoxides utilizing 10 mol% Mn‐reduced **cat7** in EtOAc in the presence of 50 mol% Coll*HCl.

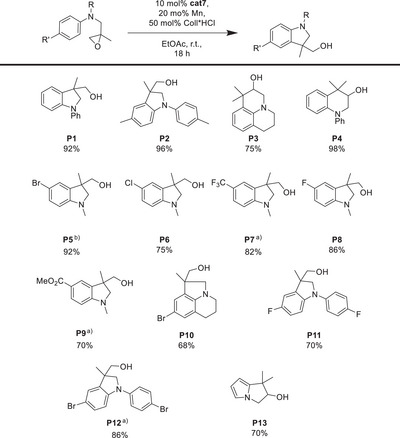

^a)^ 60 °C.

^b)^ 24 h.

Electron‐donating and electron‐withdrawing substituents are tolerated, tricyclic systems, in particular the strained **P10**, are accessible and the reaction can also be carried out with pyrroles. Notably, **P12** can be obtained with **cat7** by increasing the temperature to 60 °C, which is a substantial advantage over previous Cp_2_Ti(III)Cl catalysis that requires either the introduction of electron‐withdrawing substituents at the cyclopentadienyl ligand or exchange of chloride for sulfonates for the preparation of **P12**.^[^ ^6^, ^7^, ^8^, ^9^, ^10^, ^11^ ^]^

## Conclusion

In summary, we have identified the active catalyst **cat7** for the epoxide radical arylation in EtOAc from a library of Ti(salen) complexes. This was achieved by analyzing two sets of predictors for successful catalysis, the redox potentials of **cat1**–**cat7** and the features of the E_q_C_r_‐equilibria of the electrochemically reduced **cat1**–**cat7**, by CV. We have been able to provide a mechanistic rationale for the superiority of **cat7** from the kinetic and thermodynamic data of the E_q_C_r_‐equilibrium and DFT calculations. The tetrasubstituted backbone of **cat7** renders chloride binding less favorable and, thus, seems to prevent an inhibition of epoxide binding more efficiently than in all other complexes investigated. **Cat7** also constitutes the most active catalyst under the alternative conditions of a reduction by Mn power in the presence of Coll*HCl.

We believe that our CV‐based catalyst identification, which exploits mechanism‐based predictors to develop superior catalysis, provides a powerful tool for the identification of other metal catalysts for catalysis in single‐electron steps.

## Supporting Information

The authors have cited additional references within Supporting Information.

## Conflict of Interests

The authors declare no conflict of interest.

## Supporting information



Supporting Information

## Data Availability

The data that support the findings of this study are available in Supporting Information of this article.
