# Supplementary material for: Identification of Ti(salen) Complexes for Efficient Catalysis in Single‐Electron Steps by Cyclic Voltammetry
Source: Angew Chem Int Ed Engl. 2025 Jun 17;64(31):e202507673. doi: 10.1002/anie.202507673 (PMC12304812; doi:10.1002/anie.202507673)

**Contents**

[1. General information 4](#_Toc192587774)

[2. Reagents 4](#_Toc192587775)

[3. General Procedures 4](#_Toc192587776)

[3.1. Synthesis of epoxides for the radical arylation (GP1) 4](#_Toc192587777)

[3.2. Radical arylation under chemical reduction conditions (GP2) 5](#_Toc192587778)

[3.3. Radical arylation under electrochemical reduction conditions (GP3) 5](#_Toc192587779)

[3.4 Synthesis of Salen-Ligands (GP4) 5](#_Toc192587780)

[3.5 Synthesis of the Catalyst (GP5) 5](#_Toc192587781)

[4. Cyclic Voltammetry Experiments 5](#_Toc192587782)

[4.1 General Infromation 5](#_Toc192587783)

[4.2 General Procedure for CV-Experiments 6](#_Toc192587784)

[5. Bulk Electrolysis 6](#_Toc192587785)

[5.1 Controlled-Potential Electrolysis 7](#_Toc192587786)

[6. Synthesis of the substrates for the radical arylation 8](#_Toc192587787)

[6.1. 2-(bromomethyl)-2-methyloxirane (E1) 8](#_Toc192587788)

[6.2. 3-(bromomethyl)-2,2-dimethyloxirane (E2) 8](#_Toc192587789)

[6.3. N-((2-methyloxiran-2-yl)methyl)-N-phenylaniline (S1) 8](#_Toc192587790)

[6.4. 4-methyl-N-((2-methyloxiran-2-yl)methyl)-N-(p-tolyl)aniline (S2) 9](#_Toc192587791)

[6.5. 1-((3,3-dimethyloxiran-2-yl)methyl)-1,2,3,4-tetrahydroquinoline (S3) 9](#_Toc192587792)

[6.6. N-((3,3-dimethyloxiran-2-yl)methyl)-N-phenylaniline (S4) 10](#_Toc192587793)

[6.7. 4-bromo-N-methyl-N-((2-methyloxiran-2-yl)methyl)aniline (S5) 10](#_Toc192587794)

[6.8. 4-chloro-N-methyl-N-(2-methylallyl)aniline (A9) 11](#_Toc192587795)

[6.9. 4-chloro-N-methyl-N-((2-methyloxiran-2-yl)methyl)aniline (S6) 12](#_Toc192587796)

[6.10. *N*-methyl-N-((2-methyloxiran-2-yl)methyl)-4-(trifluoromethyl)aniline (S7) 12](#_Toc192587797)

[6.11. methyl 4-(methyl(2-methylallyl)amino)benzoate (A9) 13](#_Toc192587798)

[6.12. methyl 4-(methyl((2-methyloxiran-2-yl)methyl)amino)benzoate (S9) 13](#_Toc192587799)

[6.13. 6-bromo-1-(2-methylallyl)-1,2,3,4-tetrahydroquinoline (A10) 14](#_Toc192587800)

[6.14. 6-bromo-1-((2-methyloxiran-2-yl)methyl)-1,2,3,4-tetrahydroquinoline (S10) 15](#_Toc192587801)

[6.15. bis(4-fluorophenyl)amine (A11) 15](#_Toc192587802)

[6.16. 4-fluoro-N-(4-fluorophenyl)-N-((2-methyloxiran-2-yl)methyl)aniline (S11) 16](#_Toc192587803)

[6.17. bis(4-bromophenyl)amine (A12) 16](#_Toc192587804)

[6.18. 4-bromo-N-(4-bromophenyl)-N-((2-methyloxiran-2-yl)methyl)aniline (S12) 17](#_Toc192587805)

[6.19. 1-((3,3-dimethyloxiran-2-yl)methyl)-1*H*-pyrrole (S13) 17](#_Toc192587806)

[7. Synthesis of the Ligands 18](#_Toc192587807)

[7.1 (1*R*,2*R*)-*N,N’*-bis(salicylidene)-1,2-cyclohexanediamine (L1) 18](#_Toc192587808)

[7.2 2,2′‑[(1*R*,2*R*)‑1,2‑Cyclohexanediylbis[(*E*)‑nitrilomethylidyne]]bis[4,6 ‑bis(1,1‑dimethylethyl) phenol (L2) 20](#_Toc192587809)

[7.3 Bis(salicylidene)-1,1,2,2-tetramethylethylenediamine (L3) 19](#_Toc192587810)

[7.4 N,N‘-Bis(3,5-di-tert-butylsalicylidene)-1,1,2,2-tetramethyl-1,2-diaminoethane (L4) 21](#_Toc192587811)

[7.5 6,6'‑((1E,1'E)‑(((1R,2R)‑1,2‑diphenylethane‑1,2‑diyl)bis(azaneylylidene))bis(methaneylylidene))bis(2,4-di-tert-butylphenol) (L5) 20](#_Toc192587812)

[8. Synthesis of the Catalysts 21](#_Toc192587813)

[8.1 (1*R*,2*R*)-Cyclohexyl-Salen- TiCl_2_ (cat2) 22](#_Toc192587814)

[8.2 ^t^Bu-(1*R*,2*R*)-Cyclohexyl-Salen- TiCl_2_ (Cat5) 24](#_Toc192587815)

[8.3 Tetramethyl-Salen-TiCl2 (Cat3) 22](#_Toc192587816)

[8.4 ^t^Bu-(1*R*,2*R*)-Diphenyl-Salen-TiCl_2_ (Cat6) 24](#_Toc192587817)

[8.5 ^t^Bu-Tetramethyl-Salen-TiCl_2_ (Cat7) 25](#_Toc192587818)

[9. Catalysis under chemical and electrochemical reduction conditions 25](#_Toc192587819)

[9.1. (3-methyl-1-phenylindolin-3-yl)methanol (P1) 25](#_Toc192587820)

[9.2. (3,5-dimethyl-1-(p-tolyl)indolin-3-yl)methanol (P2) 27](#_Toc192587821)

[9.3. 1,1-dimethyl-2,3,6,7-tetrahydro-1H,5H-pyrido[3,2,1-ij]quinolin-2-ol (P3) 27](#_Toc192587822)

[9.4. 4,4-dimethyl-1-phenyl-1,2,3,4-tetrahydroquinolin-3-ol (P4) 28](#_Toc192587823)

[9.5. (5-bromo-1,3-dimethylindolin-3-yl)methanol (P5) 29](#_Toc192587824)

[9.6. (5-chloro-1,3-dimethylindolin-3-yl)methanol (P6) 30](#_Toc192587825)

[9.7. (1,3-dimethyl-5-(trifluoromethyl)indolin-3-yl)methanol (P7) 30](#_Toc192587826)

[9.8. (5-fluoro-1,3-dimethylindolin-3-yl)methanol (P8) 31](#_Toc192587827)

[9.9. methyl 3-(hydroxymethyl)-1,3-dimethylindoline-5-carboxylate (P9) 32](#_Toc192587828)

[9.10. (8-bromo-1-methyl-1,2,5,6-tetrahydro-4H-pyrrolo[3,2,1-ij]quinolin-1-yl)methanol (P10) 32](#_Toc192587829)

[9.11. (5-fluoro-1-(4-fluorophenyl)-3-methylindolin-3-yl)methanol (P11) 33](#_Toc192587830)

[9.12. (5-bromo-1-(4-bromophenyl)-3-methylindolin-3-yl)methanol (P12) 34](#_Toc192587831)

[9.13. 1,1-dimethyl-2,3-dihydro-1H-pyrrolizin-2-ol (P13) 34](#_Toc192587832)

[10. Deviation of the rate law 35](#_Toc192587833)

[11. CV data 37](#_Toc192587834)

[12. Simulation of the CV data 40](#_Toc192587835)

[13. Computational details 44](#_Toc192587836)

[14. References 62](#_Toc192587837)

[15. NMR Spectra 64](#_Toc192587838)

# General information

All moisture- or oxygen-sensitive reactions were carried out under inert atmosphere (Ar) using standard *Schlenk* and vacuum line technique. The solvents used were purified and deoxygenated by distillation (THF, EtOAc dried over CaH_2_ applied to freeze-pump-thaw cycles for CV experiments and electrolysis), by an M Braun MB-SPS-800 system (DMF, THF) or used as received when stored in AcrosSeal® bottles (EtOAc for chemical reduction). Other solvents were either distilled under air (cyclohexane (CH), ethyl acetate (EA)) or used without further purification (Et_2_O, NEt_3_). All reactions were monitored by thin-layer chromatography (TLC) on Merck silica gel 60 F254 plates using UV light as visualizing agent (if applicable) or a solution of ammoniummolybdate tetrahydrate (25 g/L) and Ce(SO_4_)_2_*4 H_2_O (10 g/L) in 10% aqueous H_2_SO_4_ followed by heating. The products were purified by flash chromatography on Merck silica gel 60 (0.035–0.070 mm). ^1^H-, ^13^C-NMR spectra were recorded on Bruker Avance I 400 MHz (^1^H-base frequency: 400.13 MHz), Bruker Avance I 500 MHz ( 1H-base frequency: 499.13 MHz), or Bruker Avance III HD Ascend 500 MHz (^1^H-base frequency: 500.13 MHz) at 298 K. Chemical shifts are denoted in ppm (δ), and calibrated by using residual nondeuterated solvent [CHCl_3_ (7.26 ppm), C_6_H_6_ (7.16 ppm) or CD_2_Cl_2_ (5.32 ppm)] as internal reference for ^1^H-NMR and the deuterated solvent [CDCl_3_ (77.2 ppm), C_6_D_6_ (128.1 ppm) or CD_2_Cl_2_ (53.8 ppm)] as internal standard for ^13^C-NMR.^[20]^ High resolution mass spectra analysis was performed on a Thermoquest MAT 95 XL instrument (Thermo Finnigan, EI/ESI), an Orbitrap XL mass spectrometer (Thermo Fisher Scientific, APCI/ESI) or MAT 90 (EI, LIFDI). IR spectra were recorded on the ATR-IR spectrometers NicoletTM 380 or Shimadzu IRSpirit. The conversion was determined from the crude ^1^H-NMR by comparing the integrals from the corresponding CH_3_ groups of substrate and product.

# Reagents

4-fluoro-N-methyl-N-((2-methyloxiran-2-yl)methyl)aniline **(S8)** was obtained from *Zhang* according to the reported procedure.^[4]^

# General Procedures

## Synthesis of epoxides for the radical arylation (GP1)

Corresponding amine was dissolved in dry DMF (0.4 M) and NaH (60% in mineral oils, 1.3 eq.) was added portion wise. The suspension was stirred for 30 mins at room temperature and 2-(bromomethyl)-2-methyloxirane was added dropwise. The suspension was stirred at room temperature overnight. Saturated ammonium chloride solution was added and the aqueous layer was extracted 3x with EtOAc. The combined organic layers were washed 2x with sat. NH_4_Cl solution, once with brine and dried over MgSO_4_. The solvent was removed under reduced pressure and the crude product was purified by column chromatography.

## Radical arylation under chemical reduction conditions (GP2)

Ti(salen)Cl_2_ (10 mol%), Mn powder (20 mol%) and freshly sublimed Collidinium chloride (50 mol%) were placed in a flame dried *Schlenk* tube and flushed with argon 3 times. EtOAc (0.1 M with regard to the substrate) was added and the solution was stirred at room temperature until a colour change from red to green was observed (typically 15 mins). The corresponding substrate (1.0 eq.) was added and the solution was stirred at room temperature for 18 h. The organic layer was washed 2x with sat. NH_4_Cl solution and dried over MgSO_4_. The solvent was removed under reduced pressure and the crude product purified by column chromatography.

## Radical arylation under electrochemical reduction conditions (GP3)

The corresponding substrate was placed in a flame dried *Schlenk* tube evacuated and put into a *Glovebox*. A solution of Ti(salen)Cl (0.01 M in EtOAc with 0.2 M NBu_4_PF_6_) was added and the resulting solution was stirred outside the *Glovebox* for 18 h. The solution was transferred with Et_2_O and filtered. The solvent was removed under reduced pressure and the crude product was purified by column chromatography.

## 3.4 Synthesis of Salen-Ligands (GP4)

The corresponding amine (1.0 eq.) was placed in a round flask equipped with K_2_CO_3_ (1.2 eq.) and water (1.3 M with regard to the amine) and the mixture was stirred for 0.5 h at room temperature. Methanol (0.3 M with regard to the amine) was added and the mixture was stirred for another 15 min. The corresponding Aldehyde (2.0 eq.) was added and the mixture refluxed for 18h. The residue was dissolved in DCM, washed 3x with water and dried with MgSO_4_. The solvent was removed under reduced pressure and the crude product was employed in the next step without further purification.

## 3.5 Synthesis of the Catalyst (GP5)

The flame dried *Schlenk* was equipped with the corresponding Salen-Ligand (1.0 eq.) and dry toluene (0.45 M with regard to the ligand). Ti(O^i^Pr)_4_ (1.0 eq.) was added and the mixture was stirred for 18 h at room temperature. Same Amount of Toluene and TMSCl (2.4 eq.) was added. The mixture turned red and was stirred for 4 h at room temperature. The crude product was filtered off under argon atmosphere and washed with dry toluene. After drying under reduced pressure, the product was obtained as red solid which was stored in the *Glovebox.*

# Cyclic Voltammetry Experiments

## 4.1 General Information

The chemicals tetrabutylammonium hexafluorophosphate (NBu_4_PF_6_), tetrabutylammonium chloride (NBu_4_Cl) and AgNO_3_ were purchased in electrochemical grade from Aldrich and stored in a glovebox under an inert atmosphere (Ar).

All CV-experiments were carried out under inert atmosphere in a glovebox and performed by an Electrochemical Analyzer 1400D (*CH-Instruments*). A glassy carbon disk of 1 mm diameter was used as a working electrode material. Its surface was polished with diamond paste (0.25 μm) provided by *Struers* followed by cleaning in an ethanol bath. A platinum coil served as the counter electrode and the reference consist of a silver wire immersed in a Pyrex tube containing NBu_4_PF_6_ (0.2 m) and NBu_4_I (0.02 m) in THF separated from the main solution by a ceramic frit. The potentials were referenced against the Fc^+^/Fc redox couple (the values of a SCE in 0.2 m NBu_4_PF_6_/THF are obtained by the addition of 0.52 V to the potential). The iR compensation mode of the *CH-Instrument* Electrochemical Analyzer was used for all cyclic voltammetry experiments.

## 4.2 General Procedure for CV-Experiments

An oven dried cyclic voltammetry cell was equipped with magnetic stir bar and filled with 10 mL freshly distilled solvent. After dissolving the conducting salt NBu_4_PF_6_ (0.775 g, 0.200 mmol) background measurements were conducted to reduce the coulomb current in the analysis by subtraction from the CVs recorded with analyte. Titan-Salen (0.02 mmol) was added to the cell and CVs were recorded. The CV-experiment was performed at different sweep rate (0.05 Vs^–1^, 0.1 Vs^–1^, 0.2 Vs^–1^, 0.5 Vs^–1^, 1 Vs^–1^, 2 Vs^–1^, 5 Vs^–1^ and 10 Vs^–1^) and the solution was stirred after the respective sweep rate. For recording the potential of Fc^+^/Fc redox couple a small amount of ferrocene (0.02 mmol) was added as an infernal reference at the end of the experiment.

# Bulk Electrolysis

The divided bulk electrolysis cell was purchased from *ALS* which is distributed by *C3 Prozess- und Analysentechnik GmbH* in Germany. All controlled potential electrolysis experiments were carried out in a glovebox and were performed by a 1400D Electrochemical Analyzer (*CH-Instruments*). A glassy carbon mesh electrode was used as the cathode and a platinum wire immersed into a solution of CH_3_CN/NBu_4_PF_6_ (0.2 M) and Cp_2_TiCl_2_ (0.01 M) served as anode. The reference electrode is composed of a silver wire and a solution of NBu_4_PF_6_ (0.1 M) and AgNO_3_ (0.01 M) in CH_3_CN.


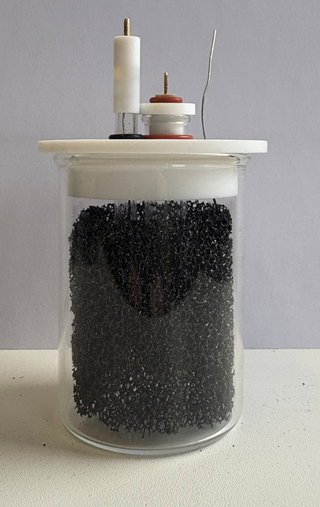


Figure S1: Bulk electrolysis cell.

## 5.1 Controlled-Potential Electrolysis

The bulk electrolysis cell (100 mL) was equipped with a stirring bar and conducting salt NBu_4_PF_6_ (3.87 g, 10.0 mmol), corresponding catalyst (0.50 mmol) and solvent (50 mL) were added inside a glovebox. After filling the anode and the reference electrode with the described solutions all the electrodes were put inside the cell and connected to the potentiostat. The stirring was set to 280 rpm and the experiment started at controlled potential (E = -1.4 V). The current flow was monitored and after it was below 1 mA the electrochemical reduction was stopped. During the experiment the color of the solution was changed from dark red (Ti^IV^) to dark green (Ti^III^) and this solution was used for performing radical arylation.


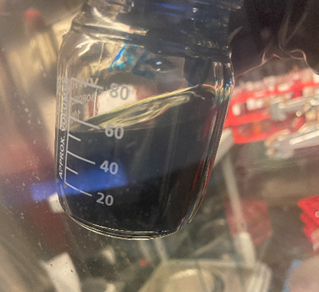


Figure S2: Electrochemically activated solution of a salen complex.

# Synthesis of the substrates for the radical arylation

## 2-(bromomethyl)-2-methyloxirane (E1)

3-bromo-2-methylprop-1-ene (1.00 eq., 50.0 mmol, 6,75 g) was dissolved in DCM (250 mL) and cooled to 0 °C *m*CPBA (70% in water, 1.50 eq., 75.0 mmol, 18.5 g) was added portion wise and the solution was warmed to room temperature overnight. The organic layer was washed 5 times with 2 M NaOH (250 mL), once with brine (250 mL) and dried over MgSO_4_. The solvent was removed under reduced pressure and the product was obtained as a colourless liquid (7.10 g, 47.0 mmol, 94%).

^1^H NMR (499 MHz, C_6_D_6_) δ 2.83 (d, *J* = 10.4 Hz, 1H), 2.77 (d, *J* = 10.4 Hz, 1H), 2.17 – 2.07 (m, 2H), 1.10 (s, 3H).

^13^C NMR (126 MHz, C_6_D_6_) δ 55.4, 54.4, 38.4, 18.9.

## 3-(bromomethyl)-2,2-dimethyloxirane (E2)

1-bromo-3-methylbut-2-ene (1.00 eq., 75.0 mmol, 11.2 g) was dissolved in DCM (225 mL) and cooled to 0 °C *m*CPBA (70% in water, 1.50 eq., 113.0 mmol, 25.9 g) was added portion wise and the solution was warmed to room temperature overnight. The organic layer was washed 5 times with 2 M NaOH (250 mL), once with brine (250 mL) and dried over MgSO_4_. The solvent was removed under reduced pressure and the product was obtained as a colourless liquid (12.3 g, 74.7 mmol, 99%).

^1^H NMR (500 MHz, C_6_D_6_) δ [ppm] 3.01 (dd, *J*=10.2, 5.9, 1H), 2.76 (dd, *J*=10.2, 7.3, 1H), 2.70 (dd, *J*=7.3, 5.9, 1H), 0.96 (s, 3H), 0.89 (s, 3H).

^13^C NMR (126 MHz, C_6_D_6_) δ [ppm] 62.0, 59.8, 30.4, 24.3, 18.0.

## N-((2-methyloxiran-2-yl)methyl)-N-phenylaniline (S1)

According to GP1 diphenylamine (1.00 eq, 23.6 mmol, 4.00 g), NaH (60% in mineral oil, 1.30 eq. 30.7 mmol, 1.23 g) and 2-(chloromethyl)-2-methyloxirane (2.0 eq., 46.8 mmol, 4.99 g) was stirred in DMF (60 mL) at room temperature overnight. After aqueous workup the crude product was purified by column chromatography (CH:EA:NEt_3_ 96:3:1, SiO_2_) to obtain **S1** (4.70 g, 19.7 mmol, 83%) as a colourless solid.

^1^H NMR (499 MHz, C_6_D_6_) δ 7.24 – 7.18 (m, 4H), 7.16 – 7.09 (m, 4H), 6.94 (tt, *J* = 7.2, 1.1 Hz, 2H), 3.72 – 3.61 (m, 2H), 2.48 (dd, *J* = 5.0, 0.8 Hz, 1H), 2.21 (d, *J* = 4.9 Hz, 1H), 1.14 (d, *J* = 0.6 Hz, 3H).

^13^C NMR (126 MHz, CDCl_3_) δ 149.0, 129.6, 121.9, 121.5, 56.9, 56.1, 51.9, 19.6.

IR (neat) [cm^-1^]: 505, 591, 611, 691, 747, 796, 852, 1074, 1238, 1361, 1449, 1493, 1588, 2925, 3037.

MS (ESI+): [M+H^+^] calculated for C_16_H_18_NO^+^: 240.1383, found: 240.1379.

## 4-methyl-N-((2-methyloxiran-2-yl)methyl)-N-(p-tolyl)aniline (S2)

According to GP1 di-*p*-tolylamine (1.00 eq., 5.00 mmol, 0.986 g), NaH (60% in mineral oils, 1.3 eq., 6.50 mmol, 0.260 g) and **E1** (2.10 eq., 10.5 mmol, 1.59 g) was stirred in DMF (12.5 mL) at room temperature overnight. After aqueous workup the crude product was purified by column chromatography (CH:EA:NEt_3_ 95:4:1, SiO_2_) to obtain **S2** (1.10 g, 5.00 mmol, 82%) as a colourless oil.

^1^H NMR (499 MHz, CDCl_3_) δ 7.04 – 6.93 (m, 8H), 3.62 (d, *J* = 1.9 Hz, 2H), 2.44 (d, *J* = 5.0 Hz, 1H), 2.16 (d, *J* = 5.1 Hz, 1H), 2.13 (s, 6H), 1.10 (d, *J* = 0.7 Hz, 3H).

^13^C NMR (126 MHz, CDCl_3_) δ 147.0, 130.7, 130.2, 121.4, 57.2, 56.2, 52.0, 20.7, 19.7.

IR (neat) [cm^-1^]: 425, 500, 540, 730, 790, 805, 1090, 1220, 1260, 1355, 1450, 1510, 2860, 2915.

MS (ESI+): [M+H^+^] calculated for C_18_H_22_NO^+^: 268.1696, found: 268.1691.

## 1-((3,3-dimethyloxiran-2-yl)methyl)-1,2,3,4-tetrahydroquinoline (S3)

In a *Schlenk* flask diisopropylamine (1.60 eq., 9.60 mmol, 1.35 mL) was dissolved in dry THF (15 mL) and cooled to 0 °C. BuLi (2.5 M in hexanes, 1.60 eq. 9.60 mmol, 3.85 mL) is added dropwise and the mixture is warmed to room temperature over the course of 20 minutes. The solution is cooled to ‑20 °C, 1,2,3,4-tetrahydroquinoline (1.00 eq., 6.00 mmol, 0.799 g) was added dropwise, the mixture is stirred 40 minutes at that temperature. At -20 °C **E2** (2.10 eq. 12.6 mmol, 2.08 g) was added and the mixture was warmed to room temperature overnight. Sat. NH_4_Cl solution (15 mL) was added, the aqueous layer was extracted three times with Et_2_O (20 mL) and the combined organic layers were dried over MgSO_4_. The solvent was removed under reduced pressure and the crude product was purified by column chromatography (CH:EA:NEt_3_ 95:4:1 to 9:9:1, SiO_2_) to obtain **S3** (0.982 g, 4.52 mmol, 75%) as a colourless oil.

^1^H NMR (499 MHz, C_6_D_6_) δ 7.13 (dddd, *J* = 8.1, 7.4, 1.7, 0.8 Hz, 1H), 6.96 (ddd, *J* = 7.2, 1.8, 1.0 Hz, 1H), 6.77 – 6.70 (m, 2H), 3.24 (dd, *J* = 15.4, 4.4 Hz, 1H), 3.18 – 3.02 (m, 2H), 2.99 – 2.91 (m, 1H), 2.77 (t, *J* = 4.9 Hz, 1H), 2.54 (td, *J* = 6.2, 2.0 Hz, 2H), 1.70 – 1.60 (m, 2H), 1.06 (s, 4H), 1.04 (s, 3H).

^13^C NMR (126 MHz, C_6_D_6_) δ 145.8, 129.6, 127.6, 123.0, 117.0, 111.8, 61.4, 57.0, 51.1, 50.0, 28.4, 24.6, 22.7, 19.1.

IR (neat) [cm^-1^]: 417, 678, 715, 741, 813, 880, 1058, 1120, 1169, 1193, 1212, 1235, 1249, 1301, 1328, 1344, 1377, 1456, 1499, 1601, 2926.

MS (ESI+): [M+H^+^] calculated for C_14_H_20_NO^+^: 218.1539, found: 218.1535.

## N-((3,3-dimethyloxiran-2-yl)methyl)-N-phenylaniline (S4)

According to GP1 diphenylamine (1.00 eq., 6.00 mmol, 1.02 g), NaH (60% in mineral oils, 1.3 eq., 7.80 mmol, 0.312 g) and **E2** (2.10 eq., 12.6 mmol, 2.08 g) was stirred in DMF (15 mL) at room temperature overnight. After aqueous workup the crude product was purified by column chromatography (CH:EA:NEt_3_ 95:4:1 to 90:9:1, SiO_2_) to obtain **S4** ((1.30 g, 5.13 mmol, 85%) as a colourless oil.

^1^H NMR (500 MHz, C_6_D_6_) δ 7.14 – 7.07 (m, 4H), 7.05 – 6.99 (m, 4H), 6.89 – 6.79 (m, 3H), 3.71 (dd, *J* = 15.5, 5.0 Hz, 1H), 3.63 (dd, *J* = 15.6, 5.1 Hz, 1H), 2.91 (t, *J* = 5.0 Hz, 1H), 0.97 (s, 3H), 0.85 (s, 3H).

^13^C NMR (126 MHz, C_6_D_6_) δ 148.6, 129.7, 122.0, 121.8, 61.4, 57.9, 52.0, 24.4, 18.8.

IR (neat) [cm^-1^]: 410, 503, 604, 691, 746, 856, 880, 1082, 1116, 1216, 1245, 1309, 1363, 1493, 1589.

MS (ESI+): [M+H^+^] calculated for C_17_H_20_NO^+^: 254.1539, found: 254.1536.

## 4-bromo-N-methyl-N-((2-methyloxiran-2-yl)methyl)aniline (S5)

In a *Schlenk* flask diisopropylamine (1.71 eq., 8.56 mmol, 1.2 mL) was dissolved in dry THF (12.5 mL) and cooled to 0 °C. BuLi (2.5 M in hexanes, 1.60 eq. 8.00 mmol, 3.2 mL) is added dropwise and the mixture is warmed to room temperature over the course of 1 h. The solution is cooled to ‑20 °C, 4‑bromo-*N*-methylaniline (1.00 eq., 4.98 mmol, 0.926 g) was added dropwise, the mixture is stirred 30 minutes at that temperature. At -20 °C **E1** (2.1 eq., 10.5 mmol, 1.59 g) was added and the mixture was warmed to room temperature overnight. Sat. NH_4_Cl solution (40 mL) was added, the aqueous layer was extracted three times with EtOAc (20 mL) and the combined organic layers were dried over MgSO_4_. The solvent was removed under reduced pressure and the crude product was purified by column chromatography (CH:EA:NEt_3_ 90:8:2, SiO_2_) to obtain **S5** (1.03 g, 4.02 mmol, 81%) as a colourless oil.

^1^H NMR (400 MHz, C_6_D_6_) δ 7.33 – 7.24 (m, 2H), 6.29 – 6.21 (m, 2H), 2.94 (d, *J* = 15.6 Hz, 1H), 2.77 (d, *J* = 15.6 Hz, 1H), 2.46 (s, 3H), 2.15 (dd, *J* = 5.0, 0.8 Hz, 1H), 2.07 (d, *J* = 5.0 Hz, 1H), 0.92 (d, *J* = 0.7 Hz, 3H).

^13^C NMR (101 MHz, C_6_D_6_) δ 149.0, 132.1, 114.2, 109.0, 56.9, 55.9, 50.9, 38.8, 19.0.

IR (neat) [cm^-1^]: 505, 620, 805, 905, 1070, 1370, 1450, 1490, 1590, 2915.

MS (ESI+): [M+H^+^] calculated for C_11_H_15_BrNO^+^: 256.0332, found: 256.0335.

## 4-chloro-N-methyl-N-(2-methylallyl)aniline (A6)

In a flask 4-chloro-N-methylaniline (1.00 eq., 10.0 mmol, 1.42 g) and K_2_CO_3_ (2.00 eq., 20.0 mmol, 2.76 g), were dissolved in 0.5 mL DMSO. . 3-chloro-2-methylpropene (1.50 eq., 15.0 mmol, 1.36 g) was added dropwise and the mixture was stirred at 70° for 18 h. After cooling DCM and H_2_O were added. The aqueous layer was extracted 3 times with DCM and the combined organic layers were washed with sat. NaCl-solution and dried over MgSO_4_. The solvent was removed under reduced pressure and the crude product was purified by column chromatography (CH:EA 95:5, SiO_2_) to obtain **A6** (1.20 g, 6.12 mmol, 61%) as a colorless oil.

^1^H NMR (499 MHz, CDCl_3_) δ 7.17 – 7.12 (m, 2H), 6.63 – 6.56 (m, 2H), 4.87 – 4.84 (m, 1H), 4.78 – 4.75 (m, 1H), 3.77 (s, 2H), 2.94 (s, 3H), 1.73 – 1.70 (m, 3H).

^13^C NMR (126 MHz, CDCl_3_) δ 148.1, 140.9, 128.8, 121.0, 113.1, 111.0, 58.9, 38.6, 20.0.

The data is in agreement with the literature.^[3]^

## 4-chloro-N-methyl-N-((2-methyloxiran-2-yl)methyl)aniline (S6)

According to the literature procedure^[2]^ in a 1:1:1 solution of THF, acetone and water (10 mL each) with a few drops of a phosphate buffer solution (ph=7) **A6** (1.00 eq., 6.12 mmol, 1.20 g,), potassium osmate dihydrate (0.01 eq., 61μmol, 22 mg) and *N*-methylmorpholine *N-*oxide (1.40 eq., 8.57 mmol, 1.00 g,) were dissolved and stirred at room temperature for 18 hours. A sat. solution of NaHSO_3_ (20 mL) and diethyl ether (20 mL). were added. The aqueous layer was extracted three times with diethyl ether (50 mL). The combined organic layers were washed three times with brine (20 mL) and dried over MgSO_4_. The solvent was removed under reduced pressure in a *Schlenk* flask and redissolved in dry THF. NaH (60% suspended in mineral oil, 4.00 eq., 24.0 mmol, 576 mg) was added slowly and the reaction mixture was stirred for 30 minutes. TsCl (1.10 eq., 6.60 mmol, 1.26 g) was added and the reaction mixture was stirred for another 18 hours. Water (20 mL) and Et_2_O (20 mL) were added and the aqueous layer was extracted three times with diethyl ether (20 mL). The combined organic layers were washed once with brine (20 mL), dried over MgSO_4_ and the solvents were removed under reduced pressure. The crude product was purified by column chromatography (CH:EA:NEt_3_, 90:9:1, SiO_2_) to obtain **S6** (969 mg, 4.58 mmol, 75 %) as a pale yellow oil.

^1^H NMR (700 MHz, CDCl_3_) δ 7.17 – 7.14 (m, 2H), 6.64 – 6.61 (m, 2H), 3.53 (d, *J* = 15.8 Hz, 1H), 3.35 (d, *J* = 15.8 Hz, 1H), 2.95 (s, 3H), 2.62 (d, *J* = 4.9 Hz, 1H), 2.60 (d, *J* = 4.8 Hz, 1H), 1.34 (d, *J* = 0.7 Hz, 3H).

^13^C NMR (700 MHz, CDCl_3_) δ 148.5, 129.0, 121.6, 113.4, 57.4, 56.7, 51.8, 39.5, 19.5.

The data is in agreement with the literature. ^[3]^

## *N*-methyl-N-((2-methyloxiran-2-yl)methyl)-4-(trifluoromethyl)aniline (S7)

According to GP1 *N*-methyl-4-(trifluoromethyl)aniline (1.00 eq., 6.00 mmol, 1.05 g), NaH (60% in mineral oils, 1.3 eq., 7.80 mmol, 0.312 g) and **E1** (2.10 eq., 12.6 mmol, 1.90 g) was stirred in DMF (15 mL) at 60 °C overnight. After aqueous workup the crude product was purified by column chromatography (CH:EA:NEt_3_ 90:9:1 to 85:14:1, SiO_2_) to obtain **S7** (0.978 g, 4.00 mmol, 67%) as a colourless liquid.

^1^H NMR (500 MHz, C_6_D_6_) δ 7.46 – 7.39 (m, 2H), 6.33 (dd, *J* = 8.5, 1.9 Hz, 2H), 2.97 (dd, *J* = 15.8, 1.7 Hz, 1H), 2.79 (dd, *J* = 15.8, 1.5 Hz, 1H), 2.47 (s, 3H), 2.13 – 2.08 (m, 1H), 2.06 (dd, *J* = 4.9, 1.3 Hz, 1H), 0.90 (s, 3H).

^13^C NMR (126 MHz, C_6_D_6_) δ 151.59, 126.36 (q, *J* = 3.8 Hz), 117.91 (q, *J* = 32.5 Hz), 111.14, 55.93, 55.36, 50.43, 38.31, 18.58.

IR (neat) [cm^-1^]: 590, 620, 820, 905, 1070, 1100, 1160, 1200, 1325, 1380, 1535, 1615.

## Methyl 4-(methyl(2-methylallyl)amino)benzoate (A9)

In a *Schlenk* flask methyl-4-(*N*-methylamino)benzoate (1.00 eq., 20.00 mmol, 3.30 g) was dissolved in dry DMF (40 mL) and sodium hydride (60% suspended in mineral oil, 1.30 eq., 26.00 mmol, 1.04 g) was added slowly and the reaction mixture was stirred for 30 minutes. 3-bromo-2-methylpropene (2.09 eq., 41.85 mmol, 5.65 g) was added dropwise and the reaction mixture was heated stirred at 60 °C for 16 h. After cooling sat. NH_4_Cl solution (40 mL) and ethyl acetate (40 mL) were added. The layers were separated and the aqueous layer was extracted three times with ethyl acetate (40 mL). The combined organic layers were washed twice with sat. NH_4_Cl solution (40 mL), once with brine (40 mL) and dried over MgSO_4_. The crude product is concentrated under reduced pressure and purified by column chromatography (CH:EA, 9:1) to obtain **A9** (4.00 g, 18.22 mmol, 91 %) as a pale-yellow oil.

^1^H-NMR (499 MHz, CDCl_3_) δ 7.88 (d, *J* = 9.2 Hz, 2H), 6.62 (d, *J* = 9.0 Hz, 2H), 4.88 – 4.84 (m, 1H), 4.75 – 4.71 (m, 1H), 3.87 (s, 2H), 3.84 (s, 3H), 3.03 (s, 3H), 1.74 – 1.70 (m, 3H).

^13^C-NMR (126 MHz, CDCl_3_) δ 167.5, 152.8, 140.2, 131.4, 117.1, 111.0, 110.7, 58.3, 51.6, 38.6, 20.1.

The data is in agreement with literature.^[3]^

## Methyl 4-(methyl((2-methyloxiran-2-yl)methyl)amino)benzoate (S9)

According to the literature procedure^[2]^ in a 1:1:1 solution of THF, acetone and water (18 mL each) with a few drops of a phosphate buffer solution (ph=7) **A9** (1.00 eq., 18.17 mmol, 3.98 g,), potassium osmate dihydrate (0.01 eq., 182 μmol, 67 mg) and *N*-methylmorpholine *N-*oxide (1.50 eq., 27.3 mmol, 3.19 g,) were dissolved and stirred at room temperature for 30 hours. A sat. solution of NaHSO_3_ (50 mL) and diethyl ether (50 mL). were added. The aqueous layer was extracted three times with diethyl ether (50 mL). The combined organic layers were washed three times with brine (20 mL) and dried over MGSO_4_. The solvent was removed under reduced pressure in a *Schlenk* flask and redissolved in dry THF. NaH (60% suspended in mineral oil, 3.90 eq., 70.20 mmol, 2.81 g) was added slowly and the reaction mixture was stirred for 30 minutes. TsCl (2.00 eq., 36.00 mmol, 6.86 g) was added and the reaction mixture was stirred for another four hours. Water (50 mL) and Et_2_O (50 mL) were added and the aqueous layer was extracted three times with diethyl ether (20 mL). The combined organic layers were washed once with brine (20 mL), dried over MgSO_4_ and the solvents were removed under reduced pressure. The crude product was purified by column chromatography (CH:EA:NEt_3_, 90:9:1, SiO_2_) to obtain **S9** (1.39 g, 5.91 mmol, 34 %) as a pale-yellow oil.

^1^H-NMR (700 MHz, C_6_D_6_) δ 8.26 – 8.12 (m, 2H), 6.45 – 6.40 (m, 2H), 3.62 (s, 3H), 3.01 (dd, *J* = 15.7, 1.6 Hz, 1H), 2.85 (dd, *J* = 15.8, 1.4 Hz, 1H), 2.52 (d, *J* = 1.2 Hz, 3H), 2.13 (d, *J* = 4.9 Hz, 1H), 2.06 (d, *J* = 5.0 Hz, 1H), 0.89 (d, *J* = 1.0 Hz, 3H).

^13^C-NMR (176 MHz, C_6_D_6_) δ 167.1, 153.0, 131.8, 118.5, 111.2, 56.1, 55.8, 51.2, 50.8, 38.7, 19.0.

MS (APCI): [M+H^+^] calculated for C_13_H_18_NO_3_^+^: 236.1208, found: 236.1207.

## 6-bromo-1-(2-methylallyl)-1,2,3,4-tetrahydroquinoline (A10)

In a flask 6-bromo-1,2,3,4-tetrahydroquinoline (1.00 eq., 10.0 mmol, 2.12 g) and K_2_CO_3_ (2.00 eq., 20.0 mmol, 2.76 g), were dissolved in 0.5 mL DMSO. 3-chloro-2-methylpropene (1.50 eq., 15.0 mmol, 1.36 g) was added dropwise and the mixture was stirred at 70° for 18 h. After cooling DCM and H_2_O were added. The aqueous layer was extracted 3 times with DCM and the combined organic layers were washed with sat. NaCl-solution and dried over MgSO_4_. The solvent was removed under reduced pressure and the crude product was purified by column chromatography (CH:EA 95:5, SiO_2_) to obtain **A10** (1.51 g, 5.70 mmol, 57 %) as a pale yellow oil.

^1^H-NMR (499 MHz, CDCl_3_) δ 7.09 – 7.02 (m, 2H), 6.32 (d, J = 8.7 Hz, 1H), 4.85 – 4.82 (m, 1H), 4.81 – 4.77 (m, 1H), 3.70 (s, 2H), 3.28 (t, J = 5.8 Hz, 2H), 2.74 (t, J = 6.3, Hz, 2H), 1.98 – 1.91 (m, 2H), 1.74 – 1.71 (m, 3H).

^13^C-NMR (126 MHz, CDCl_3_) δ 144.5, 140.5, 131.3, 129.70, 124.2, 112.5, 110.6, 107.2, 57.5, 49.8, 28.2, 22.2, 20.2.

The data is in agreement with literature.^[3]^

## 6-bromo-1-((2-methyloxiran-2-yl)methyl)-1,2,3,4-tetrahydroquinoline (S10)

According to the literature procedure^[2]^ in a 1:1:1 solution of THF, acetone and water (10 mL each) with a few drops of a phosphate buffer solution (ph=7) **A10** (1.00 eq., 5.65 mmol, 1.50 g,), potassium osmate dihydrate (0.01 eq., 56μmol, 21 mg) and *N*-methylmorpholine *N-*oxide (1.40 eq., 7.91 mmol, 926 mg) were dissolved and stirred at room temperature for 18 hours. A sat. solution of NaHSO_3_ (20 mL) and dietyl ether (20 mL). were added. The aqueous layer was extracted three times with diethyl ether (50 mL). The combined organic layers were washed three times with brine (20 mL) and dried over MgSO_4_. The solvent was removed under reduced pressure in a *Schlenk* flask and redissolved in dry THF. NaH (60% suspended in mineral oil, 4.00 eq., 22.6 mmol, 904 mg) was added slowly and the reaction mixture was stirred for 30 minutes. TsCl (1.10 eq., 6.21 mmol, 1.18 g) was added and the reaction mixture was stirred for another 18 hours. Water (20 mL) and Et_2_O (20 mL) were added and the aqueous layer was extracted three times with diethyl ether (20 mL). The combined organic layers were washed once with brine (20 mL), dried over MgSO_4_ and the solvents were removed under reduced pressure. The crude product was purified by column chromatography (CH:EA:NEt_3_, 90:9:1, SiO_2_) to obtain **S10** (969 mg, 4.58 mmol, 51 %) as a brown oil.

^1^H NMR (499 MHz, CDCl_3_) δ 7.09 (dd, *J* = 8.7, 2.5 Hz, 1H), 7.04 – 7.02 (m, 1H), 6.43 (d, *J* = 8.8 Hz, 1H), 3.46 (d, *J* = 15.8 Hz, 1H), 3.37 – 3.25 (m, 4H), 2.71 (t, *J* = 6.3 Hz, 2H), 2.65 (d, *J* = 4.8 Hz, 1H), 2.60 (d, *J* = 4.8 Hz, 1H), 1.93 – 1.87 (m, 2H), 1.35 (s, 3H).

^13^C NMR (126 MHz, CDCl_3_) δ 144.8, 131.6, 129.7, 124.4, 112.6, 107.7, 56.5, 56.0, 51.9, 50.9, 28.2, 22.0, 19.5.

The data is in agreement with the literature. ^[3]^

## bis(4-fluorophenyl)amine (A11)

In a *Schlenk* flask 1-fluoro-4-iodobenzene (1.00 eq-, 15.0 mmol, 3.33 g) is dissolved in dry toluene (40 mL). Pd(OAc)_2_ (0.15 eq., 2.25 mmol, 0.505 g), Cs_2_CO_3_ (1.50 eq., 22.5 mmol, 7.33g), XPhos (0.15 eq., 2.25 mmol, 1.07 g) and 4-fluoroaniline (1.00 eq., 15.0 mmol, 1.67 g) are added and the mixture is stirred at 90 °C overnight. The suspension is cooled to room temperature, washed once with water (40 mL), once with brine (40 mL) and dried over MgSO_4_. The solvent is removed under reduced pressure and the crude product is purified by column chromatography (CH:EA, 90:10, SiO_2_) to obtain **A11** (2.09 g, 10.2 mmol, 68%) as a brown solid.

^1^H NMR (400 MHz, CDCl_3_) δ 6.98-6.94 (m, 8H)

The data is in agreement with the literature. ^[22]^

## 4-fluoro-N-(4-fluorophenyl)-N-((2-methyloxiran-2-yl)methyl)aniline (S11)

According to GP1 **A11** (1.00 eq., 9.84 mmol, 2.02 g), NaH (60% in mineral oils, 1.3 eq., 12.8 mmol, 0.512 g) and **E1** (2.10 eq., 20.7 mmol, 3.12 g) was stirred in DMF (25 mL) at 60 °C overnight. After aqueous workup the crude product was purified by column chromatography (CH:EA:NEt_3_ 90:9:1, SiO_2_) to obtain **S11** ((2.03 g, 7.37 mmol, 75%) as a colourless liquid.

^1^H NMR (499 MHz, C_6_D_6_) δ 6.80 – 6.72 (m, 4H), 6.71 – 6.63 (m, 4H), 3.35 (d, *J* = 15.9 Hz, 1H), 3.29 (d, *J* = 15.9 Hz, 1H), 2.26 (d, *J* = 4.9 Hz, 1H), 2.10 (d, *J* = 5.0 Hz, 1H), 0.99 (s, 3H).

^13^C NMR (126 MHz, C_6_D_6_) δ 158.6 (d, *J* = 240.6 Hz), 145.3 (d, *J* = 2.6 Hz), 122.7 (d, *J* = 7.8 Hz), 116.2 (d, *J* = 22.2 Hz), 57.6, 55.9,51.7, 19.5.

IR (neat) [cm^-1^]: 536, 579, 780, 819, 1086, 1210, 1499.

MS (EI): [M^+●^] calculated for C_16_H_15_F_2_NO^●+^: 275,1116, found: 275,1113.

## bis(4-bromophenyl)amine (A12)

Diphenylamine (1.00 eq., 40 mmol, 6.77 g) was dissolved in DMF (40 mL) and cooled to 0°C.*N*-bromosuccinimide (2.00 eq , 80 mmol,)14.24 g) was added portion wise and the reaction mixture was stirred at 0°C for 6 hours. The crude reaction mixture was diluted with water (80 mL) and ethyl acetate (80 mL). The aqueous layer was extracted three times with ethyl acetate (40 mL). The combined organic layers were washed twice with water (40 mL), once with brine (40 mL) and dried over MgSO_4_. The solvents were removed under reduced pressure and the crude product was purified by column chromatography (CH:EA, 95:5 to 9:1, SiO_2_) to obtain **A12** (11.10 g, 33.94 mmol, 85 %) as a colourless solid.

^1^H NMR (499 MHz, CDCl_3_) δ 7.39 – 7.33 (m, 4H), 6.96 – 6.88 (m, 4H), 5.64 (s, 1H).

^13^C NMR (126 MHz, CDCl_3_) δ 141.9, 132.5, 119.7, 113.6.

The NMR is in agreement with literature.^[3]^

## 4-bromo-N-(4-bromophenyl)-N-((2-methyloxiran-2-yl)methyl)aniline (S12)

According to GP1 **A12** (1.00 eq., 19.1 mmol, 6.26 g), NaH (60% in mineral oils, 1.3 eq., 24.9 mmol, 0.995 g) and **E1** (2.10 eq., 40.2 mmol, 6.07 g) was stirred in DMF (40 mL) at room temperature overnight. After aqueous workup the crude product was purified by column chromatography (CH:EA:NEt_3_ 96:3:1, SiO_2_) to obtain **S12** ((5.61 g, 14.1 mmol, 74%) as a colourless solid.

^1^H NMR (499 MHz, C_6_D_6_) δ 7.21 – 7.17 (m, 4H), 6.60 – 6.53 (m, 4H), 3.31 (d, *J* = 16.0 Hz, 1H), 3.20 (d, *J* = 16.1 Hz, 1H), 2.19 (d, *J* = 4.8 Hz, 1H), 2.05 (d, *J* = 4.8 Hz, 1H), 0.92 (d, *J* = 4.8 Hz, 3H).

^13^C NMR (126 MHz, C_6_D_6_) δ 147.3, 132.6, 123.0, 114.8, 56.7, 55.7, 51.6, 19.4.

IR (neat) [cm^-1^]: 460, 500, 513, 684, 756, 790, 816, 998, 1086, 1192, 1233, 1256, 1354, 1483, 1579.

MS (ESI+): [M+H^+^] calculated for C_16_H_16_Br_2_NO^+^: 397.9575, found: 397.9572.

## 1-((3,3-dimethyloxiran-2-yl)methyl)-1*H*-pyrrole (S13)

In a *Schlenk* flask 1*H*-pyrrole (1.00 eq., 5.00 mmol, 0.335 g) was dissolved in dry DMSO (6.25 mL), freshly ground KOH (2.00 eq., 10.0 mmol, 0.561 g) was added and the solution was stirred for 20 mins. **E2** (1.50 eq., 7.50 mmol, 1.24 g) was added dropwise and the mixture is stirred at room temperature overnight. Sat. NH_4_Cl solution (6 mL) is added and the aqueous layer is extracted three times with EtOAc (10 mL each). The combined organic layer is washed once with water (20 mL), once with brine (20 mL) and dried over MgSO_4_. The solvent is removed under reduced pressure, the crude product was purified by column chromatography and obtained as a yellow oil (0.721 g, 4.77 mmol, 95%).

^1^H NMR (400 MHz, C_6_D_6_) δ 6.55 (t, *J* = 2.1 Hz, 2H), 6.36 (t, *J* = 2.1 Hz, 2H), 3.47 (dd, *J* = 14.5, 6.2 Hz, 1H), 3.35 (dd, *J* = 14.5, 5.3 Hz, 1H), 2.56 (dd, *J* = 6.2, 5.3 Hz, 1H), 0.93 (s, 6H).

^13^C NMR (101 MHz, C_6_D_6_) δ 120.8, 109.3, 62.3, 57.5, 49.0, 24.3, 18.6.

The data is in agreement with literature.^[28]^

# Synthesis of the Ligands

- 1. **2,2'-((1E,1'E)-(ethane-1,2-diylbis(azaneylylidene))bis(methaneylylidene))diphenol (L1)**

In a round bottom flask, salicylic aldehyde (2.00 eq., 10.0 mmol, 1.22 g) was dissolved in MeOH (33 mL). Ethylenediamine (1.00 eq., 5.00 mmol, 0.30 mg) was added and the mixture was stirred under reflux overnight. The solid was filtered off and washed with MeOH (20 mL). The solvent from the filtrate was removed and **L1** (1.30 g, 4.85 mmol, 97%) was obtained from both the filtrate and the filtration residue as a yellow solid.

^1^H NMR (500 MHz, CDCl_3_) δ [ppm] 13.2 (s, 2H), 8.4 (s, 2H), 7.3 (ddd, *J*=8.3, 7.3, 1.7, 2H), 7.2 (dd, *J*=7.6, 1.7, 2H), 6.9 (ddt, *J*=8.3, 1.1, 0.5, 2H), 6.9 (td, *J*=7.5, 1.1, 2H), 3.9 (s, 4H).

^13^C NMR (126 MHz, CDCl_3_) δ [ppm] 166.6, 161.2, 132.5, 131.6, 118.8, 117.1, 59.9.

The data is in agreement with literature.^[29]^

## (1*R*,2*R*)-*N,N’*-bis(salicylidene)-1,2-cyclohexanediamine (L2)

According to GP4 (1*R*,2*R*)-*trans*-cyclohexane-1,2-diammonium *L*-tartrate (1.0 eq., 5.0 mmol, 1.31 g), K_2_CO_3_ (1.2 eq., 5.0 mmol, 0.69 g), H_2_O (4.0 mL) and Salicylaldehyde (1.0 eq., 10.0 mmol, 1.07 mL) were refluxed in MeOH (17.5 mL). After aqueous work up the crude product was obtained as yellow solid (1.55 g, 4.81 mmol, 96 %).

^1^H NMR (500 MHz, CDCl_3_) δ 8.26 (s, 2H), 7.27 – 7.21 (m, 2H), 7.14 (dd, *J* = 7.6, 1.7 Hz, 2H), 6.91 – 6.87 (m, 2H), 6.79 (td, *J* = 7.5, 1.1 Hz, 2H), 3.36 – 3.28 (m, 2H), 1

.99 – 1.83 (m, 4H), 1.79 – 1.68 (m, 2H), 1.54 – 1.42 (m, 2H).

^13^C NMR (126 MHz, CDCl_3_) δ 164.9, 161.1, 132.3, 131.6, 118.8, 118.7, 116.9, 72.8, 33.2, 24.3.

The data is in agreement with literature.^[23]^

## Bis(salicylidene)-1,1,2,2-tetramethylethylenediamine (L3)

According to GP4 2,3-dimethyl-2,3-Butanediamine hydrochloride (1.0 eq., 3.00 mmol, 567 mg), K_2_CO_3_ (1.0 eq., 3.00 mmol, 414 mg), H_2_O (2.25 mL) and Salicylaldehyde (2.0 eq., 6.00 mmol, 733 mg) were refluxed in MeOH (10.5 mL). After aqueous work up the crude product was obtained as yellow solid (942 mg, 2.9 mmol, 97 %).

^1^H NMR (500 MHz, CD_2_Cl_2_) δ [ppm] 13.95 (s, 2H), 8.40 (s, 2H), 7.29 (td, *J* = 7.6, 1.5 Hz, 4H), 6.93 – 6.85 (m, 4H), 1.38 (s, 12H).

^13^C NMR (126 MHz, CD_2_Cl_2_) δ [ppm] 162.2, 162.0, 132.6, 132.2, 119.6, 118.9, 117.3, 65.8, 23.4.

IR (neat) [cm^-1^]: 425, 450, 496, 542, 562, 647, 737, 749, 781, 830, 891, 944, 977, 984, 1032, 1109, 1126, 1151, 1171, 1217, 1277, 1366, 1379, 1415, 1456, 1496, 1580, 1624, 2981, 3065.

MS (ESI+): [M+H^+^] calculated for C_20_H_25_N_2_O_2_^+^: 325.1911, found: 325,1910.

- 1. **6,6'-((1E,1'E)-(ethane-1,2-diylbis(azaneylylidene))bis(methaneylylidene))bis(2,4-di-tert-butylphenol) (L4)**

In a round bottom flask, 3,5-di-tert-butyl-2-hydroxybenzaldehyde (2.00 eq., 10.0 mmol, 2.34 g) was dissolved in MeOH (33 mL). Ethylenediamine (1.00 eq., 5.00 mmol, 0.30 mg) was added and the mixture was stirred under reflux overnight. The solid was filtered off and washed with MeOH (20 mL) and Et_2_O (20 mL). The solvent from the filtrate was removed and **L4** (2.39 g, 4.85 mmol, 97%) was obtained from both the filtrate and the filtration residue as a bright yellow solid.

^1^H NMR (500 MHz, CDCl_3_) δ = 13.63 (s, 2H), 8.40 (s, 2H), 7.38 (d, *J*=2.5, 2H), 7.08 (d, *J*=2.5, 2H), 3.39 (s, 4H), 1.45 (s, 18H), 1.30 (s, 18H).

^13^C NMR (126 MHz, CDCl_3_) δ [ppm] 167.7, 158.2, 140.2, 136.8, 127.2, 126.2, 118.0, 59.8, 35.2, 34.3, 31.6, 29.6.

The data is in agreement with literature.^[30]^

## 2,2′‑[(1*R*,2*R*)‑1,2‑Cyclohexanediylbis[(*E*)‑nitrilomethylidyne]]bis[4,6 ‑bis(1,1‑dimethylethyl) phenol (L5)

According to GP4 (1*S*,2*S*)-*trans*-cyclohexane-1,2-diammonium *L*-tartrate (1.0 eq., 5.00 mmol, 1.31 g), K_2_CO_3_ (1.0 eq., 5.00 mmol, 691 mg), H_2_O (4 mL) and 3,5-Di-*tert*-butylsalicylaldehyde (2.0 eq., 10 mmol, 2.34 g) were refluxed in MeOH (18 mL). After aqueous work up the crude product was obtained as yellow solid (2.48 g, 4.54 mmol, 91 %).

^1^H NMR (500 MHz, CDCl_3_) δ 13.72 (s, 2H), 8.34 (s, 2H), 7.35 (d, *J* = 2.5 Hz, 2H), 7.02 (d, *J* = 2.4 Hz, 2H), 3.39 – 3.32 (m, 2H), 2.02 – 1.96 (m, 2H), 1.93 – 1.88 (m, 2H), 1.83 – 1.72 (m, 2H), 1.53 – 1.47 (m, 2H), 1.46 (s, 18H), 1.28 (s, 18H).

^13^C NMR (126 MHz, CDCl_3_) δ 166.0, 158.2, 140.0, 136.5, 126.9, 126.2, 118.0, 77.2, 72.6, 35.1, 34.2, 33.4, 31.6, 29.6, 24.5.

The data is in agreement with literature.^[24]^

## 6,6'-((1E,1'E)-(((1R,2R)-1,2-diphenylethane-1,2-diyl)bis(azaneylylidene)) bis(methaneylylidene))bis(2,4-di-tert-butylphenol) (L6)

In a round flask (1*R*,2*R*)-1,2-diphenylethane-1,2-diamine (1.0 eq., 5.00 mmol, 1.06 g) and 3,5-Di-*tert*-butylsalicylaldehyde (2.0 eq., 10.0 mmol, 2.34 g) were refluxed in MeOH (15 mL). The solvent was removed under reduced pressure and the crude product was obtained as yellow solid (3.07 g, 4.77 mmol, 95 %).

^1^H NMR (500 MHz, C_6_D_6_) δ 14.06 (s, 2H), 7.92 (s, 2H), 7.49 (d, *J* = 2.4 Hz, 2H), 7.19 – 7.16 (m, 5H), 7.03 – 6.98 (m, 4H), 6.98 – 6.94 (m, 2H), 6.88 (d, *J* = 2.4 Hz, 2H), 4.49 (s, 2H), 1.63 (s, 19H), 1.25 (s, 19H).

^13^C NMR (126 MHz, C_6_D_6_) δ 167.9, 158.8, 140.3, 140.2, 136.9, 128.6, 128.4, 127.7, 127.4, 126.9, 118.6, 80.3, 35.4, 34.2, 31.7, 29.9.

The data is in agreement with literature.^[26]^

## N,N‘-Bis(3,5-di-tert-butylsalicylidene)-1,1,2,2-tetramethyl-1,2-diaminoethane (L7)

According to GP4 2,3-dimethyl-2,3-Butanediamine hydrochloride (1.0 eq., 1.20 mmol, 226 mg), K_2_CO_3_ (1.0 eq., 1.20 mmol, 166 mg), H_2_O (1.0 mL) and 3,5-Di-*tert*-butylsalicylaldehyde (2.0 eq., 2.40 mmol, 562 mg) were refluxed in MeOH (4.5 mL). After aqueous work up the crude product was obtained as yellow solid (588 mg, 1.07 mmol, 89 %).

^1^H NMR (500 MHz, CD_2_Cl_2_) δ 14.38 (s, 2H), 8.42 (s, 2H), 7.36 (d, *J* = 2.4 Hz, 2H), 7.14 (d, *J* = 2.4 Hz, 2H), 1.43 (s, 19H), 1.30 (s, 17H).

^13^C NMR (126 MHz, CD_2_Cl_2_) δ 163.3, 159.0, 140.4, 137.0, 127.3, 126.9, 118.6, 65.5, 54.0, 35.5, 34.6, 31.8, 29.8, 23.5.

The data is in agreement with literature.^[25]^

# Synthesis of the Catalysts

## En-salen-TiCl_2_ (cat1)

A flame dried *Schlenk* was charged with dry toluene (10 mL) and **L1**(1.0 eq, 3.03 mmol, 812 mg) and Ti(OiPr)4 (1.0 eq, 3.03 mmol, 860 mg) and the mixture was stirred for 18h at room temperature. Subsequently TMSCl (4.2 eq, 12.6 mmol, 1.60 mL) was added. The reaction mixture turned red and was stirred for an additional 20 hours. The crude product was filtered off under argon atmosphere and was washed with dry toluene. After drying under reduced pressure, the product (887 mg, 2.30 mmol, 76 %) was obtained as a red solid which was stored in the *Glovebox*.

^1^H NMR (500 MHz, CD_2_Cl_2_) δ = 8.41 (s, 2H), 7.65 – 7.55 (m, 4H), 7.13 (ddd, *J*= 7.7, 7.3, 1.1, 2H), 6.84 (ddt, *J*= 8.2, 1.1, 0.5, 2H), 4.23 (t, *J*= 0.7, 4H).

^13^C NMR (126 MHz, CD_2_Cl_2_) δ = 163.3, 162.3, 136.5, 134.7, 125.0, 122.8, 116.1, 58.8.

IR (neat) [cm^-1^]: 449, 508, 567, 657, 762, 814, 911, 1126, 1218, 1249, 1269, 1392, 1438, 1556, 1595, 1613.

MS (LIFDI): [M-Cl^-^] calculated for C_16_H_14_ClN_2_O_2_Ti^+^ 349.0220, found: 349.0229.

## (1*R*,2*R*)-Cyclohexyl-Salen- TiCl_2_ (cat2)

According to GP5 **L2** (1.0 eq., 4.80 mmol, 1.56 g), Ti(O^i^Pr)_4_ (4.8 mmol, 1.0 M in toluene) and TMSCl (2.4 eq., 11.5 mmol, 1.46 mL) were stirred in Toluene (11 mL). After filtration and washing the product (1.86 g, 4.24 mmol, 88 %) was obtained as red solid.

^1^H NMR (500 MHz, CDCl_3_) δ 8.34 (s, 2H), 7.58 – 7.51 (m, 4H), 7.11 – 7.06 (m, 2H), 6.86 (d, *J* = 8.2 Hz, 2H), 4.11 – 4.05 (m, 2H), 2.61 – 2.54 (m, 2H), 2.15 – 2.07 (m, 2H), 1.66 – 1.57 (m, 2H), 1.48 – 1.41 (m, 2H).

^13^C NMR (126 MHz, CDCl_3_) δ 162.6, 159.7, 136.6, 135.2, 125.5, 122.9, 116.5, 77.2, 67.9, 28.8, 24.2.

The data is in agreement with literature.^[8]^

## Tetramethyl-Salen-TiCl2 (Cat3)

According to GP5 **L3** (1.0 eq., 1.00 mmol, 324 mg), Ti(O^i^Pr)_4_ (1.00 mmol., 1.0 M in toluene) and TMSCl (3.0 eq., 3.00 mmol, 0.38 mL) were stirred in Toluene (2.2 mL). After filtration and washing the product (321 mg, 0.7 mmol, 73 %) was obtained as red solid.

^1^H NMR (499 MHz, CDCl_3_) δ 8.53 (s, 2H), 7.60 – 7.51 (m, 4H), 7.05 (td, *J* = 7.6, 1.1 Hz, 2H), 6.84 (d, *J* = 8.2 Hz, 2H), 1.60 (s, 13H).

^13^C NMR (126 MHz, CDCl_3_) δ 163.6, 137.3, 135.7, 125.2, 122.5, 116.2, 73.6, 28.5.

IR (neat) [cm^-1^]: 416, 451, 472, 488, 522, 541, 574, 659, 700, 735, 753, 765, 818, 847, 912, 933, 953, 960, 986, 1033, 1122, 1141, 1155, 1220, 1254, 1277, 1366, 1380, 1397, 1443, 1471, 1551, 1595, 2360, 2980, 3057.

MS (LIFDI): [M-Cl^-^] calculated for C_20_H_22_ClN_2_O_2_Ti^+^: 405.0846, found: 405.0841.

## En-^t^Bu-salen-TiCl_2_ (cat4)

A flame dried *Schlenk* was charged with dry toluene (10 mL) and **L4**(1.0 eq, 3.48 mmol, 1.71 g) and Ti(OiPr)4 (1.0 eq, 3.49 mmol, 991 mg) and the mixture was stirred for 24h at room temperature. Subsequently TMSCl (4.3 eq, 15.0 mmol, 1.90 mL) was added. The reaction mixture turned red and was stirred for an additional 27 hours. The crude product was filtered off under argon atmosphere and was washed with dry toluene. After drying under reduced pressure, the product (1.33 g, 2.18 mmol, 63 %) containing residual toluene was obtained as a red solid which was stored in the *Glovebox*.

^1^H NMR (500 MHz, CD_2_Cl_2_) δ = 8.37 (s, 2H), 7.66 (d, *J*= 2.4, 2H), 7.40 (d, *J*= 2.4, 2H), 4.19 (t, *J*= 0.7, 4H), 1.51 (s, 18H), 1.36 (s, 18H).

^13^C NMR (126 MHz, CD_2_Cl_2_) δ = 164.6, 160.1, 145.4, 137.2, 131.9, 130.1, 125.8, 59.0, 35.9, 34.9, 31.5, 30.0.

IR (neat) [cm^-1^]: 429, 491, 569, 588, 599, 630, 738, 761, 837, 869, 1249, 1547, 1562, 1622.

MS (APCI): [M-Cl^-^] calculated for C_32_H_46_ClN_2_O_2_Ti^+^ 573.2726, found: 573.2728.

## ^t^Bu-(1*R*,2*R*)-Cyclohexyl-Salen- TiCl_2_ (Cat5)

According to GP5 **L5** (1.0 eq., 2.00 mmol, 1.09 g), Ti(O^i^Pr)_4_ (2.00 mmol, 1.0 M in toluene) and TMSCl (2.4 eq., 4.80 mmol, 0.61 mL) were stirred in Toluene (4.5 mL). After filtration and washing the product (1.30 g, 1.96 mmol, 98 %) was obtained as a red solid.

^1^H NMR (499 MHz, CDCl_3_) δ 8.30 (s, 2H), 7.60 (d, *J* = 2.4 Hz, 2H), 7.35 (d, *J* = 2.4 Hz, 2H), 4.04 (dd, *J* = 7.8, 3.5 Hz, 2H), 2.58 (d, *J* = 11.7 Hz, 2H), 2.07 (d, *J* = 9.1 Hz, 2H), 1.56 (m, 2H), 1.53 (s, 18H), 1.42 (m, 2H), 1.33 (s, 18H).

^13^C NMR (126 MHz, CDCl_3_) δ 160.6, 159.9, 144.7, 136.9, 131.3, 130.2, 129.2, 128.4, 125.9, 125.4, 67.8, 35.7, 34.6, 31.5, 30.0, 28.6, 24.2.

The data is in agreement with literature.^[27]^

## ^t^Bu-(1*R*,2*R*)-Diphenyl-Salen-TiCl_2_ (Cat6)

According to GP5 **L6** (1.0 eq., 4.00 mmol, 2.58 g), Ti(O^i^Pr)_4_ (1.0 eq., 4.00 mmol, 1.0 M in Toluene) and TMSCl (2.4 eq., 21.60 mmol, 1.22 mL) were stirred in Toluene (9 mL). After filtration and washing the product (2.5 g, 3.64 mmol, 91 %) was obtained as red solid.

^1^H NMR (500 MHz, CDCl_3_) δ 7.88 (d, *J* = 1.0 Hz, 2H), 7.59 (s, 1H), 7.35 – 7.29 (m, 6H), 7.21 – 7.06 (m, 4H), 7.00 (d, *J* = 2.4 Hz, 2H), 5.55 (s, 2H), 1.56 (s, 18H), 1.25 (s, 17H).

^13^C NMR (126 MHz, CDCl_3_) δ 164.4, 159.7, 144.9, 137.0, 135.9, 131.6, 130.3, 129.5, 129.2, 125.9, 35.7, 34.6, 31.4, 30.0.

The data is in agreement with the literature.^[29]^

## ^t^Bu-Tetramethyl-Salen-TiCl_2_ (Cat7)

According to GP5 **L7** (1.0 eq., 9.00 mmol, 4.94 g), Ti(O^i^Pr)_4_ (1.0 eq., 9.00 mmol, 1.0 M in Toluene) and TMSCl (2.4 eq., 21.60 mmol, 2.74 mL) were stirred in Toluene (20 mL). After filtration and washing the product (5.39 g, 8.10 mmol, 90 %) was obtained as a red solid.

^1^H NMR (499 MHz, CD_2_Cl_2_) δ 8.53 (s, 2H), 7.66 (d, *J* = 2.4 Hz, 2H), 7.45 (d, *J* = 2.4 Hz, 2H), 1.56 (s, 12H), 1.50 (s, 18H), 1.37 (s, 18H).

^13^C NMR (126 MHz, CD_2_Cl_2_) δ 165.0, 160.8, 144.9, 136.9, 132.4, 131.1, 125.9, 74.0, 36.0, 35.0, 31.6, 30.1, 28.5.

IR (neat) [cm^-1^]: 418, 482, 498, 532, 548, 624, 643, 669, 759, 769, 815, 844, 868, 884, 917, 948, 973, 1030, 2253, 1179, 1201, 1254, 1274, 1297, 1329, 1362, 1379, 1393, 1435, 1459, 1508, 1544, 1560, 1600, 2366, 2864, 2901, 2947.

MS (LIFDI): [M-Cl^-^] calculated for C_36_H_54_ClN_2_O_2_Ti^+^ 629.3348, found: 629.3341.

# Catalysis under chemical and electrochemical reduction conditions

## (3-methyl-1-phenylindolin-3-yl)methanol (P1)

Chemical reduction:

According to GP2 **S1** (1.00 eq., 0.50 mmol, 120 mg), **cat7** (0.10 eq., 0.05 mmol, 33 mg) and Mn (0.20 eq., 0.10 mmol, 5.5 mg) were dissolved in dry EtOAc (5 mL). The solution was stirred for 18 h at room temperature. From the crude ^1^H-NMR the conversion towards the product could be determined to be 74%.

According to GP2 **S1** (1.00 eq., 0.50 mmol, 120 mg), **cat7** (0.10 eq., 0.05 mmol, 33 mg), Mn (0.20 eq., 0.10 mmol, 5.5 mg) and Coll*HCl (0.50 eq., 0.25 mmol, 39 mg) were dissolved in dry EtOAc (5 mL). The solution was stirred for 18 h at room temperature. After aqueous workup the crude product was purified by column chromatography (Pentane:Et2O:NEt3 60:38:2, SiO_2_) to obtain **P1** (110 mg, 0.46 mmol, 92%) as a colourless oil.

Electrochemical reduction:

According to GP3 **S1** (1.00 eq., 0,50 mmol, 120 mg) was dissolved in a solution (0.01 mM) of reduced **cat7** in THF (5 mL). The solution was stirred for 18 h at room temperature. From the crude ^1^H-NMR the conversion towards the product could be determined to be 27%.

According to GP3 **S1** (1.00 eq., 0,50 mmol, 120 mg) was dissolved in a solution (0.01 mM) of reduced **cat5** in EtOAc (5 mL). The solution was stirred for 18 h at room temperature. From the crude ^1^H-NMR the conversion towards the product could be determined to be 13%.

According to GP3 **S1** (1.00 eq., 0,50 mmol, 120 mg) was dissolved in a solution (0.01 mM) of reduced **cat3** in EtOAc (5 mL). The solution was stirred for 18 h at room temperature. From the crude ^1^H-NMR the conversion towards the product could be determined to be 17%.

According to GP3 **S1** (1.00 eq., 0,50 mmol, 120 mg) was dissolved in a solution (0.01 mM) of reduced **cat6** in EtOAc (5 mL). The solution was stirred for 18 h at room temperature. After workup the crude product was purified by column chromatography (Pentane:Et_2_O:NEt_3_ 60:38:2, SiO_2_) to obtain **P1** (22 mg, 0.09 mmol, 18%) as a colourless oil.

According to GP3 **S1** (1.00 eq., 0,50 mmol, 120 mg) was dissolved in a solution (0.01 mM) of reduced **cat7** in EtOAc (5 mL). The solution was stirred for 18 h at room temperature. After workup the crude product was purified by column chromatography (Pentane:Et_2_O:NEt_3_ 60:38:2, SiO_2_) to obtain **P1** (98 mg, 0.40 mmol, 79%) as a colourless oil.

^1^H NMR (499 MHz, C_6_D_6_) δ 7.23 – 7.17 (m, 2H), 7.14 – 7.09 (m, 2H), 7.06 – 6.99 (m, 1H), 6.93 (dd, *J* = 7.3, 1.4 Hz, 1H), 6.89 (tt, *J* = 7.3, 1.2 Hz, 1H), 6.76 (td, *J* = 7.3, 0.9 Hz, 1H), 3.61 (d, *J* = 9.3 Hz, 1H), 3.29 (d, *J* = 3.4 Hz, 1H), 3.27 (d, *J* = 4.7 Hz, 1H), 3.20 (d, *J* = 10.6 Hz, 1H), 1.39 (d, *J* = 4.8 Hz, 0H), 1.14 (s, 3H).

^13^C NMR (126 MHz, C_6_D_6_) δ 147.0, 144.4, 136.3, 129.5, 128.4, 123.6, 121.2, 119.4, 118.0, 108.9, 68.8, 61.7, 45.5, 22.1.

IR (neat) {cm^-1^]: 472, 494, 692, 740, 1021, 1332, 1460, 1482, 1499, 1589, 2863, 2922, 2958, 3350.

MS (ESI+): [M+H^+^] calculated for C_16_H_18_NO^+^: 240.1383, found: 240.1379.

## (3,5-dimethyl-1-(p-tolyl)indolin-3-yl)methanol (P2)

Chemical reduction:

According to GP2 **S2** (1.00 eq., 0,50 mmol, 134 mg), **cat7** (0.10 eq., 0.05 mmol, 33 mg), Mn (0.20 eq., 0.10 mmol, 5.5 mg) and Coll*HCl (0.50 eq., 0.25 mmol, 39 mg) were dissolved in dry EtOAc (5 mL). The solution was stirred for 18 h at room temperature. After aqueous workup the crude product was purified by column chromatography (Pentane:Et_2_O:NEt_3_ 60:38:2, SiO_2_) to obtain **P2** (132 mg, 0.49 mmol, 98%) as a colourless oil.

Electrochemical reduction:

According to GP3 **S2** (1.00 eq., 0,50 mmol, 134 mg) was dissolved in a solution (0.01mM) of reduced **cat7** in EtOAc (5 mL). The solution was stirred for 18 h at room temperature. After workup the crude product was purified by column chromatography (Pentane:Et_2_O:NEt_3_ 60:38:2, SiO_2_) to obtain **P2** (118 mg, 0.44 mmol, 88%) as a colourless oil.

^1^H NMR (499 MHz, C_6_D_6_) δ 7.15 – 7.08 (m, 3H), 7.08 – 7.01 (m, 2H), 6.89 – 6.82 (m, 2H), 3.67 (d, *J* = 9.2 Hz, 1H), 3.35 (d, *J* = 9.7 Hz, 2H), 3.27 (d, *J* = 10.6 Hz, 1H), 2.20 (d, *J* = 2.7 Hz, 6H), 1.19 (s, 3H).

^13^C NMR (126 MHz, C_6_D_6_) δ 145.4, 142.5, 136.4, 130.1, 130.0, 128.6, 124.4, 118.1, 108.7, 69.0, 62.2, 45.6, 22.2, 21.0, 20.8.

MS (APCI): [M+H]^+^ calculated: 268.1696, found: 268.1696.

IR (neat) [cm^-1^]: 414, 469, 493, 515, 555, 586, 756, 1001, 1018, 1327, 1370, 1454, 1484, 1513, 1608, 2918, 3424.

## 1,1-dimethyl-2,3,6,7-tetrahydro-1H,5H-pyrido[3,2,1-ij]quinolin-2-ol (P3)

Chemical reduction:

According to GP2 **S3** (1.00 eq., 0,50 mmol, 109 mg), **cat7** (0.10 eq., 0.05 mmol, 33 mg), Mn (0.20 eq., 0.10 mmol, 5.5 mg) and Coll*HCl (0.50 eq., 0.25 mmol, 39 mg) were dissolved in dry EtOAc (5 mL). The solution was stirred for 18 h at room temperature. After aqueous workup the crude product was purified by column chromatography (Pentane:Et_2_O:NEt_3_ 68:30:2, SiO_2_) to obtain **P3** (82 mg, 0.38mmol, 75%) as a colourless oil.

Electrochemical reduction:

According to GP3 **S3** (1.00 eq., 0,50 mmol, 109 mg) was dissolved in a solution (0.01mM) of reduced **cat7** in EtOAc (5 mL). The solution was stirred for 18 h at room temperature. After workup the crude product was purified by column chromatography (Pentane:Et_2_O:NEt_3_ 60:38:2, SiO_2_) to obtain **P3** (80 mg, 0.37 mmol, 73%) as a colourless oil.

^1^H NMR (400 MHz, C_6_D_6_) δ 7.06 (ddd, *J* = 7.7, 1.6, 0.8 Hz, 1H), 6.81 (ddt, *J* = 7.4, 1.8, 1.0 Hz, 1H), 6.72 (t, *J* = 7.5 Hz, 1H), 3.43 (dd, *J* = 5.1, 2.7 Hz, 1H), 2.98 (dd, *J* = 11.4, 2.7 Hz, 1H), 2.78 (dd, *J* = 11.4, 5.1 Hz, 1H), 2.75 – 2.63 (m, 2H), 2.61 – 2.46 (m, 2H), 1.89 (s, 1H), 1.69 – 1.52 (m, 2H), 1.38 (s, 3H), 1.10 (s, 3H).

^13^C NMR (101 MHz, C_6_D_6_) δ 141.0, 129.4, 127.6, 125.6, 121.9, 117.7, 72.4, 53.1, 50.5, 38.2, 30.7, 28.2, 25.6, 22.3.

MS (EI): [M^+•^] calculated 217.1461 found 217.1462.

IR (neat) [cm^-1^]:475, 495, 515, 680, 750, 765, 780, 800, 830, 850, 900, 915, 1000, 1015, 1030, 1105, 1130, 1160, 1175, 1215, 1260, 1310, 1360, 1380, 1440, 1460, 1505, 1595, 2855, 2930, 3310.

## 4,4-dimethyl-1-phenyl-1,2,3,4-tetrahydroquinolin-3-ol (P4)

Chemical reduction:

According to GP2 **S4** (1.00 eq., 0,50 mmol, 126 mg), **cat7** (0.10 eq., 0.05 mmol, 33 mg), Mn (0.20 eq., 0.10 mmol, 5.5 mg) and Coll*HCl (0.50 eq., 0.25 mmol, 39 mg) were dissolved in dry EtOAc (5 mL). The solution was stirred for 18 h at room temperature. After aqueous workup the crude product was purified by column chromatography (Pentane:Et_2_O:NEt_3_ 60:38:2, SiO_2_) to obtain **P4** (124 mg, 0.49 mmol, 98%) as a colourless oil.

Electrochemical reduction:

According to GP3 **S4** (1.00 eq., 0,50 mmol, 127 mg) was dissolved in a solution (0.01mM) of reduced **cat7** in EtOAc (5 mL). The solution was stirred for 18 h at room temperature. After workup the crude product was purified by column chromatography (Pentane:Et_2_O:NEt_3_ 60:38:2, SiO_2_) to obtain **P4** (98 mg, 0.39 mmol, 78%) as a colourless oil.

^1^H NMR (500 MHz, C_6_D_6_) δ 7.21 (dd, *J* = 7.7, 1.5 Hz, 1H), 7.15 – 7.10 (m, 2H), 7.08 – 7.02 (m, 2H), 6.95 – 6.90 (m, 1H), 6.90 – 6.84 (m, 2H), 6.79 (ddd, *J* = 7.7, 6.8, 1.7 Hz, 1H), 3.45 – 3.39 (m, 2H), 3.31 (dd, *J* = 12.3, 6.4 Hz, 1H), 1.32 (s, 4H), 1.14 (s, 3H).

^13^C NMR (126 MHz, C_6_D_6_) δ 148.4, 142.8, 131.7, 129.8, 127.5, 127.0, 124.7, 124.1, 119.7, 116.5, 73.2, 53.5, 38.2, 29.0, 24.6.

MS (APCI): [M+H]^+^ calculated: 254,1539, found: 254.1539.

IR (neat) [cm^-1^]: 493, 560, 695, 735, 1001, 1040, 1057, 1200, 1241, 1275, 1301, 1443, 1460, 1591, 2866, 2964, 3390.

## (5-bromo-1,3-dimethylindolin-3-yl)methanol (P5)

Chemical reduction:

According to GP2 **S5** (1.00 eq., 0,50 mmol, 128 mg), **cat7** (0.10 eq., 0.05 mmol, 33 mg), Mn (0.20 eq., 0.10 mmol, 5.5 mg) and Coll*HCl (0.50 eq., 0.25 mmol, 39 mg) were dissolved in dry EtOAc (5 mL). The solution was stirred for 24 h at room temperature. After aqueous workup the crude product was purified by column chromatography (CH:EA:NEt_3_ 80:18:2, SiO_2_) to obtain **P5** (118 mg, 0.46 mmol, 92%) as a colourless oil.

Electrochemical reduction:

According to GP3 **S5** (1.00 eq., 0,50 mmol, 127 mg) was dissolved in a solution (0.01mM) of reduced **cat7** in EtOAc (5 mL). The solution was stirred for 18 h at room temperature. After workup the crude product was purified by column chromatography (CH:EA:NEt_3_ 80:18:2, SiO_2_) to obtain **P5** (92 mg, 0.36 mmol, 73%) as a colourless oil.

^1^H NMR (500 MHz, C_6_D_6_) δ 7.20 (dd, *J* = 8.3, 2.1 Hz, 1H), 7.11 (d, *J* = 2.0 Hz, 1H), 5.97 (d, *J* = 8.3 Hz, 1H), 3.21 (d, *J* = 10.5 Hz, 1H), 3.11 (d, *J* = 10.5 Hz, 1H), 3.06 (d, *J* = 8.8 Hz, 1H), 2.54 (d, *J* = 8.8 Hz, 1H), 2.24 (s, 3H), 1.00 (s, 3H).

^13^C NMR (126 MHz, C_6_D_6_) δ 152.2, 137.6, 131.1, 126.2, 109.8, 108.8, 68.3, 65.2, 46.2, 35.1, 21.8.

MS (APCI): [M+H]^+^ calculated 256.0332, found 256.0330.

IR (neat) [cm^-1^]: 418, 476, 569, 592, 629, 738, 800, 1030, 1070, 1259, 1462, 1489, 1598, 2810, 2862, 3344.

## (5-chloro-1,3-dimethylindolin-3-yl)methanol (P6)

Chemical reduction:

According to GP2 **S6** (1.00 eq., 0.50 mmol, 106 mg), **cat7** (0.10 eq., 0.05 mmol, 33 mg), Mn (0.20 eq., 0.10 mmol, 5.5 mg) and Coll*HCl (0.50 eq., 0.25 mmol, 39 mg) were dissolved in dry EtOAc (5 mL). The solution was stirred for 18 h at 60 °C. After aqueous workup the crude product was purified by column chromatography (CH:EA:NEt_3_ 80:19:1, SiO_2_) to obtain **P6** (79 mg, 0.37 mmol, 75%) as a colourless oil.

Electrochemical reduction:

According to GP3 **S6** (1.00 eq., 0.50 mmol, 106 mg) was dissolved in a solution (0.01 mM) of reduced **cat7** in EtOAc (5 mL). The solution was stirred for 18 h at room temperature. After workup the crude product was purified by column chromatography (CH:EA:NEt_3_ 80:19:1, SiO_2_) to obtain **P6** (85 mg, 0.40 mmol, 80%) as a colourless oil.

^1^H NMR (500 MHz, CDCl_3_) δ 7.12 (dd, *J* = 8.3, 2.1 Hz, 1H), 7.01 (d, *J* = 2.2 Hz, 1H), 6.52 (d, *J* = 8.3 Hz, 1H), 3.63 (d, *J* = 10.8 Hz, 1H), 3.60 – 3.54 (m, 2H), 3.07 (d, *J* = 9.3 Hz, 1H), 2.80 (s, 3H), 1.33 (s, 3H).

^13^C NMR (126 MHz, CDCl_3_) δ 137.3, 129.7, 128.4, 123.4, 121.9, 110.0, 68.9, 65.6, 46.6, 37.0, 22.0.

The data is in agreement with the literature.^[3]^

## (1,3-dimethyl-5-(trifluoromethyl)indolin-3-yl)methanol (P7)

Chemical reduction

According to GP2 **S7** (1.00 eq., 0,50 mmol, 123 mg), **cat7** (0.10 eq., 0.05 mmol, 33 mg), Mn (0.20 eq., 0.10 mmol, 5.5 mg) and Coll*HCl (0.50 eq., 0.25 mmol, 39 mg) were dissolved in dry EtOAc (5 mL). The solution was stirred for 18 h at 60 °C. After aqueous workup the crude product was purified by column chromatography (Pentane:Et_2_O:NEt_3_ 63:35:2, SiO_2_) to obtain **P7** (101 mg, 0.41 mmol, 82%) as a colourless solid.

Electrochemical reduction:

According to GP3 **S7** (1.00 eq., 0,50 mmol, 127 mg) was dissolved in a solution (0.01mM) of reduced **cat7** in EtOAc (5 mL). The solution was stirred for 18 h at room temperature. After workup the crude product was purified by column chromatography (CH:EA:NEt_3_ 80:18:2, SiO_2_) to obtain **P7** (75 mg, 0.30 mmol, 61%) as a colourless oil.

^1^H NMR (400 MHz, C_6_D_6_) δ 7.37 (ddd, *J* = 8.2, 1.8, 0.9 Hz, 1H), 7.27 (d, *J* = 1.9 Hz, 1H), 6.00 (d, *J* = 8.3 Hz, 1H), 3.17 (d, *J* = 10.5 Hz, 1H), 3.10 (d, *J* = 9.0 Hz, 1H), 3.06 (d, *J* = 10.5 Hz, 1H), 2.57 (d, *J* = 9.0 Hz, 1H), 2.22 (s, 3H), 1.38 (s, 0H), 0.99 (s, 3H).

^13^C NMR (101 MHz, C_6_D_6_) δ 155.4, 135.4, 126.6 (q, *J* = 4.1 Hz), 120.0 (q, *J* = 3.6 Hz), 119.2 (q, *J* = 31.9 Hz), 105.9, 68.3, 64.7, 45.8, 34.1, 21.9.

IR (neat) [cm^-1^]: 415, 480, 510, 585, 630, 810, 1053, 1070, 1100, 1155, 1275, 1320, 1335, 1450, 1510, 1620, 2830, 2870, 2924, 2960, 3380.

MS (EI) calculated [M^●+^] for C_12_H_14_F_3_NO^•+^ 245.1022, found 245.1022.

## (5-fluoro-1,3-dimethylindolin-3-yl)methanol (P8)

Chemical reduction:

According to GP2 **S8** (1.00 eq., 0,51 mmol, 100 mg), **cat7** (0.10 eq., 0.05 mmol, 33 mg), Mn (0.20 eq., 0.10 mmol, 5.5 mg) and Coll*HCl (0.50 eq., 0.25 mmol, 39 mg) were dissolved in dry EtOAc (5 mL). The solution was stirred for 18 h at room temperature. After aqueous workup the crude product was purified by column chromatography (CH:EA:NEt_3_ 80:18:2, SiO_2_) to obtain **P8** (86 mg, 0.44 mmol, 86%) as a colourless oil.

Electrochemical reduction:

According to GP3 **S8** (1.00 eq., 0.50 mmol, 98 mg) was dissolved in a solution (0.01mM) of reduced **cat7** in EtOAc (5 mL). The solution was stirred for 18 h at room temperature. After workup the crude product was purified by column chromatography (CH:EA:NEt_3_, 80:19:1, SiO_2_) to obtain **P8** (83 mg, 0.42 mmol, 83%) as a colourless oil.

^1^H NMR (499 MHz, C_6_D_6_) δ 6.78 (ddd, *J* = 9.3, 8.5, 2.7 Hz, 1H), 6.71 (dd, *J* = 8.2, 2.7 Hz, 1H), 6.05 (dd, *J* = 8.5, 4.1 Hz, 1H), 3.29 (d, *J* = 10.4 Hz, 1H), 3.19 (d, *J* = 10.4 Hz, 1H), 3.09 (d, *J* = 8.7 Hz, 1H), 2.57 (d, *J* = 8.7 Hz, 1H), 2.30 (s, 3H), 1.06 (s, 3H).

^13^C NMR (126 MHz, C_6_D_6_) δ 157.3 (d, *J* = 234.6 Hz), 149.6 (d, *J* = 1.3 Hz), 137.0 (d, *J* = 7.2 Hz), 114.2 (d, *J* = 23.1 Hz), 110.9 (d, *J* = 23.8 Hz), 107.8 (d, *J* = 8.0 Hz), 68.3, 65.9, 46.2 (d, *J* = 1.7 Hz), 36.1, 21.6.

IR (neat) [cm^-1^]: 456, 484, 581, 682, 697, 803, 903, 1030, 1193, 1264, 1464, 1492, 2807, 2863, 2955, 3347.

MS (EI): [M^+•^] calculated 195.1054 found 195.1055.

## methyl 3-(hydroxymethyl)-1,3-dimethylindoline-5-carboxylate (P9)

Chemical reduction:

According to GP2 **S9** (1.00 eq., 0,50 mmol, 118 mg), **cat7** (0.10 eq., 0.05 mmol, 33 mg), Mn (0.20 eq., 0.10 mmol, 5.5 mg) and Coll*HCl (0.50 eq., 0.25 mmol, 39 mg) were dissolved in dry EtOAc (5 mL). The solution was stirred for 18 h at 60 °C. After aqueous workup the crude product was purified by column chromatography (CH:EA:NEt_3_ 80:18:2, SiO_2_) to obtain **P9** (83 mg, 0.35 mmol, 70%) as a colourless solid.

^1^H NMR (500 MHz, C_6_D_6_) δ 8.18 (dd, *J* = 8.3, 1.7 Hz, 1H), 7.96 (d, *J* = 1.7 Hz, 1H), 6.10 (d, *J* = 8.3 Hz, 1H), 3.63 (s, 3H), 3.28 (d, *J* = 10.5 Hz, 1H), 3.19 (d, *J* = 9.1 Hz, 1H), 3.16 (d, *J* = 10.5 Hz, 1H), 2.64 (d, *J* = 9.1 Hz, 1H), 2.25 (s, 3H), 1.06 (s, 3H).

^13^C NMR (126 MHz, C_6_D_6_) δ 167.3, 156.4, 134.6, 132.2, 124.7, 119.5, 105.4, 68.6, 64.6, 51.2, 45.8, 33.7, 22.3.

MS (APCI): [M+H]^+^ calculated 236.1281, found: 236.1280.

IR (neat) [cm^-1^]: 480, 770, 1035, 1100, 1190, 1215, 1255, 1290, 1440, 1605, 2835, 2865, 2950, 3430.

## (8-bromo-1-methyl-1,2,5,6-tetrahydro-4H-pyrrolo[3,2,1-ij]quinolin-1-yl)methanol (P10)

Chemical reduction:

According to GP2 **S10** (1.00 eq., 0,50 mmol, 138 mg), **cat7** (0.10 eq., 0.05 mmol, 33 mg), Mn (0.20 eq., 0.10 mmol, 5.5 mg) and Coll*HCl (0.50 eq., 0.25 mmol, 39 mg) were dissolved in dry EtOAc (5 mL). The solution was stirred for 18 h at room temperature. After aqueous workup the crude product was purified by column chromatography (CH:EA:NEt_3_ 85:13:2, SiO_2_) to obtain **P10** (96 mg, 0.35 mmol, 70%) as a colourless solid.

^1^H NMR (400 MHz, CDCl_3_) δ 6.97 (dt, *J* = 1.9, 1.0 Hz, 1H), 6.96 – 6.94 (m, 1H), 3.60 (d, *J* = 2.8 Hz, 2H), 3.39 (d, *J* = 8.7 Hz, 1H), 3.07 (dt, *J* = 10.5, 4.6 Hz, 1H), 2.95 (d, *J* = 8.8 Hz, 1H), 2.86 – 2.80 (m, 1H), 2.67 – 2.62 (m, 2H), 2.08 – 2.00 (m, 2H), 1.31 (s, 3H).

^13^C NMR (101 MHz, CDCl_3_) δ 148.8, 134.9, 129.8, 124.9, 123.6, 121.5, 121.3, 119.2, 109.9, 68.8, 64.4, 47.0, 46.6, 23.9, 22.7, 21.7.

The data is in agreement with literature.^[3]^

## (5-fluoro-1-(4-fluorophenyl)-3-methylindolin-3-yl)methanol (P11)

Chemical reduction:

According to GP2 **S11** (1.00 eq., 0,50 mmol, 138 mg), **cat7** (0.10 eq., 0.05 mmol, 33 mg), Mn (0.20 eq., 0.10 mmol, 5.5 mg) and Coll*HCl (0.50 eq., 0.25 mmol, 39 mg) were dissolved in dry EtOAc (5 mL). The solution was stirred for 18 h at room temperature. After aqueous workup the crude product was purified by column chromatography (CH:EA:NEt_3_ 85:13:2, SiO_2_) to obtain **P11**(96 mg, 0.35 mmol, 70%) as a colourless solid.

^1^H NMR (500 MHz, C_6_D_6_) δ 6.87 – 6.80 (m, 2H), 6.78 – 6.66 (m, 4H), 6.64 (ddd, *J* = 8.6, 4.3, 0.5 Hz, 1H), 3.45 (d, *J* = 9.3 Hz, 1H), 3.17 (d, *J* = 10.5 Hz, 1H), 3.13 – 3.06 (m, 2H), 1.02 (s, 3H).

^13^C NMR (126 MHz, C_6_D_6_) δ 158.8 (d, *J* = 89.8 Hz), 156.9 (d, *J* = 86.4 Hz), 143.6 (d, *J* = 1.6 Hz), 140.8 (d, *J* = 2.4 Hz), 138.0 (d, *J* = 7.1 Hz), 119.7 (d, *J* = 7.6 Hz), 116.1 (d, *J* = 22.3 Hz), 114.1 (d, *J* = 23.0 Hz), 111.4 (d, *J* = 23.9 Hz), 108.5 (d, *J* = 7.9 Hz), 68.4, 62.61, 45.6 (d, *J* = 1.7 Hz), 21.8.

MS (APCI): [M+H]^+^ calculated: 276.1194, found 276.1192.

IR (neat) [cm^-1^]: 496, 515, 548, 558, 588, 803, 830, 1031, 1176, 1212, 1462, 1483, 1509, 1605, 2853, 3335.

## (5-bromo-1-(4-bromophenyl)-3-methylindolin-3-yl)methanol (P12)

Chemical reduction:

According to GP2 **S12** (1.00 eq., 0.50 mmol, 199 mg), **cat7** (0.10 eq., 0.05 mmol, 33 mg), Mn (0.20 eq., 0.10 mmol, 5.5 mg) and Coll*HCl (0.50 eq., 0.25 mmol, 39 mg) were dissolved in dry EtOAc (5 mL). The solution was stirred for 18 h at room temperature. After aqueous workup the crude product was purified by column chromatography (Pentane:Et_2_O:NEt_3_ 60:38:2 to 40:58:2, SiO_2_) to obtain **P12** (170 mg, 0.43 mmol, 86%) as a colourless solid.

^1^H NMR (300 MHz, C_6_D_6_) δ 7.31 – 7.17 (m, 2H), 7.13 – 7.07 (m, 2H), 6.66 – 6.52 (m, 3H), 3.34 (d, *J* = 9.5 Hz, 1H), 3.05 (d, *J* = 10.6 Hz, 1H), 2.96 (dd, *J* = 10.0, 5.6 Hz, 2H), 1.37 (s, 0H), 0.92 (s, 3H).

^13^C NMR (176 MHz, C_6_D_6_) δ 145.5, 142.8, 139.0, 132.4, 131.0, 126.9, 119.4, 113.7, 111.4, 110.2, 68.4, 61.8, 45.5, 21.9.

IR (neat) [cm^-1^]: 494, 799, 1033, 1325, 1373, 1479, 1487, 1582, 2865, 2924, 2958, 3324.

MS (EI): [M^●+^] calculated for C_16_H_15_Br_2_NO^●+^: 394.9515, found: 394.9508.

## 1,1-dimethyl-2,3-dihydro-1H-pyrrolizin-2-ol (P13)

Chemical reduction:

According to GP2 **S13** (1.00 eq., 0.50 mmol, 75 mg), **cat7** (0.10 eq., 0.05 mmol, 33 mg), Mn (0.20 eq., 0.10 mmol, 5.5 mg) and Coll*HCl (0.50 eq., 0.25 mmol, 39 mg) were dissolved in dry EtOAc (5 mL). The solution was stirred for 18 h at room temperature. After aqueous workup the crude product was purified by column chromatography (CH:E:NEt_3_ 80:19:1, SiO_2_) to obtain **P13** (52 mg, 0.35 mmol, 70%) as a yellow oil.

^1^H NMR (700 MHz, C_6_D_6_) δ 6.37 (t, *J* = 3.1 Hz, 1H), 6.35 (dd, *J* = 2.6, 1.2 Hz, 1H), 5.94 (dd, *J* = 3.4, 1.2 Hz, 1H), 3.80 (t, *J* = 5.8 Hz, 1H), 3.60 (dd, *J* = 10.7, 6.2 Hz, 1H), 3.31 (dd, *J* = 10.7, 5.5 Hz, 1H), 1.10 (d, *J* = 1.1 Hz, 3H), 1.08 (d, *J* = 1.1 Hz, 3H).

^13^C NMR (176 MHz, C_6_D_6_) δ 143.5, 113.4, 111.9, 98.4, 82.3, 52.1, 41.8, 26.3, 21.4.

The data is in agreement with literature.^[3]^

# Derivation of the rate law

Scheme S1: Schematic catalytic cycle for the arylation reaction.

The reaction laws for the intermediates RadA, RadB and [LTiCl] are given by eq. (1) – (3)

$\frac{\partial RadA}{\partial t}=k_{2}\left[ LTiCl \right]\left[ \boldsymbol{S} \right]-k_{3}\left[ RadA \right]$ (1)

$\frac{\partial RadB}{\partial t}=k_{3}\left[ RadA \right]-k_{4}\left[ RadB \right]$ (2)

$\frac{\partial\left[ LTiCl \right]}{\partial t}=k_{1}\left[ {LTiCl}_{2}^{-} \right]-k_{-1}\left[ LTiCl \right]\left[ {Cl}^{-} \right]-k_{2}\left[ LTiCl \right]\left[ \boldsymbol{S} \right]+k_{4}\left[ RadB \right]$ (3)

Steady state approximation for the reactive intermediates is applied, because their concentration is small compared to the sum of the concentrations of **S** and **P** and their change in concentration over time equals 0. The concentrations of the intermediates are then given by eq. (4) and (5) assuming steady state conditions for eq. (1) and (2).

$\left[ RadA \right]=\frac{k_{2}}{k_{3}}\left[ LTiCl \right]\left[ \boldsymbol{S} \right]$ (4)

$\left[ RadB \right]=\frac{k_{3}}{k_{4}}\left[ RadA \right]=\frac{k_{2}}{k_{4}}\left[ LTiCl \right]\left[ \boldsymbol{S} \right]$ (5)

Accordingly, the concentration of [LTiCl] (eq. (6)) is given by the law of mass action for *K*_1_, which is equal to eq. (3) assuming steady state conditions and substitution of eq. (4) and (5) in (3).

$K_{1}=\frac{k_{1}}{k_{-1}}=\frac{\left[ LTiCl \right]\left[ {Cl}^{-} \right]}{\left[ {LTiCl}_{2}^{-} \right]}$ (6)

The total concentration Ti^tot^ of all Titanium species during catalysis equals the initial concentration of the catalyst and it is given by the sum of concentrations of the individual titanium species, eq. (7).

$\left[ {Ti}^{tot} \right]_{0}=\left[ LTiCl \right]+\left[ {LTiCl}_{2}^{-} \right]+\left[ RadA \right]+\left[ RadB \right]$ (7)

Substituting the concentrations of RadA and RadB in eq. (7) by eq. (4) and (5), respectively, as well as rearranging gives eq. (8)

$\left[ {LTiCl}_{2}^{-} \right]=\left[ {Ti}^{tot} \right]_{0}-\left[ LTiCl \right]-\frac{k_{2}}{k_{3}}\left[ LTiCl \right]\left[ \boldsymbol{S} \right]-\frac{k_{2}}{k_{4}}\left[ LTiCl \right]\left[ \boldsymbol{S} \right]$ (8)

Substitution of eq. (8) in the law of mass action *K*_1_ (eq. (6)) expresses the concentration of [LTiCl] in dependence on the starting concentration of the catalyst.

$\left[ LTiCl \right]=\frac{K_{1}\left[ {Ti}^{tot} \right]_{0}}{\left[ {Cl}^{-} \right]+K_{1}+\frac{k_{2}K_{1}}{k_{3}}\left[ \boldsymbol{S} \right]+\frac{k_{2}K_{1}}{k_{4}}\left[ \boldsymbol{S} \right]}$ (9)

The rate of formation of the Product **P** equals the consumption of Epoxide **S** assuming no side reactions and thus, the rate law for **P** is given by eq. (10) in dependence on the [LTiCl] and by eq. (11) in dependence of the starting concentration of the catalyst.

$\frac{\partial\left[ P \right]}{\partial t}=k_{4}\left[ RadB \right]=-\frac{\partial\left[ S \right]}{\partial t}=k_{2}\left[ LTiCl \right]\left[ \boldsymbol{S} \right]$ (10)

$\frac{\partial\left[ P \right]}{\partial t}=\frac{k_{2}K_{1}\left[ {Ti}^{tot} \right]_{0}\left[ \boldsymbol{S} \right]}{\left[ {Cl}^{-} \right]+K_{1}+\frac{k_{2}K_{1}}{k_{3}}\left[ \boldsymbol{S} \right]+\frac{k_{2}K_{1}}{k_{4}}\left[ \boldsymbol{S} \right]}$ (11)

# CV data

Figure S3: Scan rate normalized CV data of **cat1** in THF, 0.1 M [nBu_4_N]PF_6_.

Figure S4: Scan rate normalized CV data of **cat2** in THF, 0.1 M [nBu_4_N]PF_6_.

Figure S5: Scan rate normalized CV data of **cat3** in THF, 0.1 M [nBu_4_N]PF_6_.

Figure S6: Scan rate normalized CV data of **cat4** in THF, 0.1 M [nBu_4_N]PF_6_.

Figure S7: Scan rate normalized CV data of **cat5** in THF, 0.1 M [nBu_4_N]PF_6_.

Figure S8: Scan rate normalized CV data of **cat6** in THF, 0.1 M [nBu_4_N]PF_6_.

Figure S9: Scan rate normalized CV data of **cat7** in THF, 0.1 M [nBu_4_N]PF_6_.

# Simulation of the CV data

The model shown in Scheme S2 has been applied for simulation of the CV data using the DigiElch software. A Butler-Volmer model with fixed *α* = 0.5 and an uncompensated resistance of 2000 Ω was used. The parameters were initially optimized for each catalyst. These optimizations led to very similar electron transfer rates for *E*^0^_1_ and *E*^0^_2_ in all catalysts, which were fixed for further refinement. The optimized parameters for each catalyst are depicted Table S1.

Scheme S2: Coupled electrochemical reduction and follow-up chloride loss equilibrium for **cat1** - **cat7**.

Table S1: Simulation parameters for the CV data depicted in Figure S3 – S9.

|  |  | *D*  /cm² s^−1^ | *E*^0^_1_  /V | *k*_s1_  /cm s^–1^ | *E*^0^_2_  /V | *k*_s2_  /cm s^–1^ | *K*_1_  /M | *k*_1_  /Ms^–1^ | *k*_–1_ /s^–1^ |
| --- | --- | --- | --- | --- | --- | --- | --- | --- | --- |
| **Cat1** |  | 8.2 10^–6^ | –1.095 | 0.01 | –0.75 | 0.002 | 0.001 | 15 | 15000 |
| **Cat2** |  | 7.5 10^–6^ | –1.125 | 0.01 | –0.76 | 0.002 | 0.002 | 40 | 20000 |
| **Cat3** |  | 7.6 10^–6^ | –1.045 | 0.01 | –0.750 | 0.002 | 0.003 | 15 | 5000 |
| **Cat4** |  | 7.2 10^–6^ | –1.265 | 0.01 | –0.865 | 0.002 | 0.03 | 275 | 9167 |
| **Cat5** |  | 6.4 10^–6^ | –1.315 | 0.01 | –0.90 | 0.002 | 0.045 | 700 | 15556 |
| **Cat6** |  | 6.3 10^–6^ | –1.285 | 0.01 | –0.790 | 0.002 | 0.045 | 4000 | 88889 |
| **Cat7** |  | 7.0 10^–6^ | –1.230 | 0.01 | –0.870 | 0.002 | 0.1 | 100 | 1000 |

Figure S10: Measured (black) and simulated (red dotted line) CV data of **cat1** in THF, 0.1 M [nBu_4_N]PF_6_; left: ν = 0.05, 0.1, 0.2. 0.5 V s–1; right: 1, 2, 5, 10 Vs^–1^; simulation according to Figure S3 with the parameter values of Table S1.

Figure S11: Measured (black) and simulated (red dotted line) CV data of **cat2** in THF, 0.1 M [nBu_4_N]PF_6_; left: ν = 0.05, 0.1, 0.2. 0.5 V s–1; right: 1, 2, 5, 10 Vs^–1^; simulation according to Figure S4 with the parameter values of Table S1.

Figure S12: Measured (black) and simulated (red dotted line) CV data of **cat3** in THF, 0.1 M [nBu_4_N]PF_6_; left: ν = 0.05, 0.1, 0.2. 0.5 V s–1; right: 1, 2, 5, 10 Vs^–1^; simulation according to Figure S5 with the parameter values of Table S1.

Figure S13: Measured (black) and simulated (red dotted line) CV data of **cat4** in THF, 0.1 M [nBu_4_N]PF_6_; left: ν = 0.05, 0.1, 0.2. 0.5 V s–1; right: 1, 2, 5, 10 Vs^–1^; simulation according to Figure S6 with the parameter values of Table S1.

Figure S14: Measured (black) and simulated (red dotted line) CV data of **cat5** in THF, 0.1 M [nBu_4_N]PF_6_; left: ν = 0.05, 0.1, 0.2. 0.5 V s–1; right: 1, 2, 5, 10 Vs^–1^; simulation according to Figure S7 with the parameter values of Table S1.

Figure S15: Measured (black) and simulated (red dotted line) CV data of **cat6** in THF, 0.1 M [nBu_4_N]PF; left: ν = 0.05, 0.1, 0.2. 0.5 V s–1; right: 1, 2, 5, 10 Vs^–1^; simulation according to Figure S8 with the parameter values of Table S1.

Figure S16: Measured (black) and simulated (red dotted line) CV data of **cat7** in THF, 0.1 M [nBu_4_N]PF_6_; left: ν = 0.05, 0.1, 0.2. 0.5 V s–1; right: 1, 2, 5, 10 Vs^–1^; simulation according to Figure S9 with the parameter values of Table S1.

# Computational details

The quantum chemical DFT calculations have been performed with the TURBOMOLE 7.4 suite of programs^[32]^ The structures are fully optimized at the TPSS-D3/def2-SVP + COSMO level of theory, which combines the TPSS meta-GGA density functional^[33]^ with the BJ-damped DFT-D3 dispersion correction^[34,35]^ and the def2-SVP basis set,^[36,37]^ using the Conductor-like Screening Model (COSMO) continuum solvation model^[38]^ for THF solvent (dielectric constant ε = 7.58 and solvent radius R_solv_ = 3.18 Å). The density-fitting RI-J approach^[36,39,40]^  is used to accelerate the geometry optimization and numerical harmonic frequency calculations^[41]^ in solution. The optimized structures are characterized by frequency analysis to identify the nature of located stationary points (no imaginary frequency for true minima and only one imaginary frequency for transition state) and to provide thermal corrections (at 298.15 K and 1 atm) according to the modified ideal gas-rigid rotor-harmonic oscillator model.^[42]^ This choice of dispersion-corrected meta-GGA functional makes the efficient exploration of all potential reaction paths possible.

The final solvation free energies in THF are computed with the COSMO-RS solvation model^[43]^ (parameter file: BP_TZVP_C30_1601.ctd) using the COSMOtherm program package^[44]^ on the above TPSS-D3 optimized structures, and corrected by +1.89 kcal·mol^-1^ to account for higher reference solute concentration of 1 mol·L^−1^ usually used in solution. To check the effects of the chosen DFT functional on the reaction energies and barriers, single-point calculations at the meta-GGA TPSS-D3^[33]^ and hybrid-meta-GGA PW6B95-D3^[45]^ levels are performed using a larger def2-TZVP basis set.^[37,46]^ The final reaction Gibbs free energies (ΔG) are determined from the electronic single-point energies plus TPSS-D3 thermal corrections and COSMO-RS solvation free energies. In our discussion, higher-level PW6B95-D3 Gibbs free energies (in kcal/mol, at 298.15 K and 1 mol/L standard state concentration) will be used unless specified otherwise.

**Table S2.** TPSS-D3/def2-SVP + COSMO computed lowest imaginary frequency (ImF), zero-point energies (ZPE), gas-phase enthalpic (Hc) and Gibbs free-energy (Gc) corrections; the COSMO-RS computed solvation enthalpic (Hsol) and Gibbs free-energy (Gsol) corrections in THF solution; TPSS-D3/def2-TZVP and PW6B95-D3/def2-TZVP single-point energies (TPSS-D3 and PW6B95-D3); the relative electronic energies (ΔE_T_ and ΔE_P_) and Gibbs free-energies (ΔG_T_ and ΔG_P_) at the TPSS-D3 and PW6B95-D3 levels; relative reaction enthalpies (ΔH_P_) at the PW6B95-D3 level. Each structure is labeled either by its molecular formula or a specific name in bold.

| Reactions | ImF | ZPE | Hc | Gc | Hsol | Gsol | TPSS-D3 | PW6B95-D3 | G_P_ | ΔE_T_ | ΔE_P_ | ΔG_P_ | ΔG_T_ | ΔH_p_ |
| --- | --- | --- | --- | --- | --- | --- | --- | --- | --- | --- | --- | --- | --- | --- |
| in THF (1M) | cm^-1^ | kcal  /mol | kcal  /mol | kcal  /mol | kcal  /mol | kcal  /mol | E_h_ | E_h_ | E_h_ | kcal  /mol | kcal  /mol | kcal  /mol | kcal  /mol | kcal  /mol |
| **Cat4** + Cl^−^ | 0 | 448.47 | 475.59 | 399.94 | -105.44 | -90.90 | -3278.31582 | -3281.03453 | -3280.53603 | 0.00 | 0.00 | *0.00* | 0.00 | 0.00 |
| **Cat4**Cl^−^ | 0 | 447.56 | 474.71 | 406.95 | -73.66 | -62.11 | -3278.39149 | -3281.10943 | -3280.55689 | -47.49 | -47.00 | *-13.09* | -13.58 | -16.11 |
|  |  |  |  |  |  |  |  |  |  |  |  |  |  |  |
| **Cat4** + **Cat7**Cl^−^ | 0 | 964.35 | 1020.81 | 881.89 | -113.09 | -90.60 | -6253.76972 | -6259.03749 | -6257.77046 | 0.00 | 0.00 | *0.00* | 0.00 | 0.00 |
| **Cat4**Cl^−^ + **Cat7** | 0 | 963.75 | 1020.45 | 881.02 | -113.40 | -91.26 | -6253.77277 | -6259.04055 | -6257.77596 | -1.91 | -1.92 | *-3.45* | -3.44 | -2.60 |
|  |  |  |  |  |  |  |  |  |  |  |  |  |  |  |
| **Cat4** + **Cat6**Cl^−^ | 0 | 996.27 | 1055.10 | 910.87 | -120.56 | -96.40 | -6558.81020 | -6564.41948 | -6563.11552 | 0.00 | 0.00 | *0.00* | 0.00 | 0.00 |
| **Cat4**Cl^−^ + **Cat6** | 0 | 995.89 | 1054.78 | 910.56 | -121.00 | -96.96 | -6558.80888 | -6564.41889 | -6563.11630 | 0.83 | 0.37 | *-0.49* | -0.03 | -0.38 |
|  |  |  |  |  |  |  |  |  |  |  |  |  |  |  |
| **Cat6** + **Cat7**Cl^−^ | 0 | 1064.21 | 1126.78 | 976.14 | -121.84 | -97.43 | -6716.16030 | -6721.93591 | -6720.52958 | 0.00 | 0.00 | *0.00* | 0.00 | 0.00 |
| **Cat6**Cl^−^ + **Cat7** | 0 | 1063.99 | 1126.74 | 975.57 | -121.72 | -97.53 | -6716.16468 | -6721.93957 | -6720.53430 | -2.74 | -2.30 | *-2.97* | -3.41 | -2.22 |
|  |  |  |  |  |  |  |  |  |  |  |  |  |  |  |
| **Cat4** + OC_2_H_4_ | 0 | 483.87 | 512.10 | 430.06 | -43.56 | -30.88 | -2971.91005 | -2974.44619 | -2973.80402 | 0.00 | 0.00 | *0.00* | 0.00 | 0.00 |
| **Cat4**_OC_2_H_4_ | 0 | 484.76 | 513.21 | 443.15 | -40.42 | -29.47 | -2971.93276 | -2974.47102 | -2973.80877 | -14.26 | -15.58 | *-2.98* | -1.66 | -11.32 |
|  |  |  |  |  |  |  |  |  |  |  |  |  |  |  |
| **Cat4** + **Cat6**_OC_2_H_4_ | 0 | 1032.82 | 1093.23 | 946.17 | -87.21 | -63.92 | -6252.35008 | -6257.77962 | -6256.36765 | 0.00 | 0.00 | *0.00* | 0.00 | 0.00 |
| **Cat6** + **Cat4**_OC_2_H_4_ | 0 | 1033.09 | 1093.29 | 946.77 | -87.75 | -64.32 | -6252.35015 | -6257.78047 | -6256.36818 | -0.04 | -0.53 | *-0.33* | 0.16 | -1.02 |
|  |  |  |  |  |  |  |  |  |  |  |  |  |  |  |
| **Cat7** + **Cat6**_OC_2_H_4_ | 0 | 1100.54 | 1164.87 | 1010.87 | -88.37 | -65.05 | -6409.70456 | -6415.29971 | -6413.78643 | 0.00 | 0.00 | *0.00* | 0.00 | 0.00 |
| **Cat6** + **Cat7**_OC_2_H_4_ | 0 | 1102.33 | 1165.89 | 1013.62 | -88.65 | -65.08 | -6409.70298 | -6415.29936 | -6413.78174 | 0.99 | 0.22 | *2.94* | 3.71 | 0.96 |
|  |  |  |  |  |  |  |  |  |  |  |  |  |  |  |
| **Cat7**_THF + OC_2_H_4_ | 0 | 626.13 | 661.83 | 566.21 | -48.90 | -35.89 | -3361.87807 | -3364.81843 | -3363.96729 | 0.00 | 0.00 | *0.00* | 0.00 | 0.00 |
| **Cat7**_OC_2_H_4_ + THF | 0 | 626.04 | 661.53 | 564.59 | -47.98 | -34.54 | -3361.87315 | -3364.81420 | -3363.96350 | 3.09 | 2.65 | *2.38* | 2.82 | 3.28 |
| **Cat7**_EtOAc + OC_2_H_4_ | 0 | 626.21 | 663.56 | 564.87 | -45.67 | -32.89 | -3437.17813 | -3440.19174 | -3439.33795 | 0.00 | 0.00 | *0.00* | 0.00 | 0.00 |
| **Cat7**_OC_2_H_4_ + EtOAc | 0 | 626.76 | 663.88 | 563.14 | -49.30 | -35.29 | -3437.17264 | -3440.18620 | -3439.33900 | 3.45 | 3.48 | *-0.66* | -0.69 | 0.16 |

**Table S3.** The TPSS-D3/def2-SVP + COSMO optimized atomic Cartesian coordinates (in Å) in THF solution. Each structure is labeled by the specific name (See also **Table S2**), followed by the number of atoms, the total energy, and the detailed atomic coordinates (in double-column text list).

**Cat4**Cl^−^.xyz

85

Energy = -3276.446034247

Ti -0.0992472 0.0881925 -0.6629799

N 1.1774685 1.6842247 -1.1951102

N -1.4087814 1.7452475 -0.6686536

O 1.4876153 -0.9393202 -0.7107911

O -1.6637748 -0.8998481 -0.2829283

Cl 0.3292675 0.5087380 1.6855486

C 0.4813555 2.9029368 -1.5945792

C 2.4919367 1.6713569 -1.2098484

C -0.7370702 3.0408112 -0.6822898

C -2.7222892 1.7128736 -0.6446958

C 2.7878376 -0.7604799 -0.6726257

C -2.9681401 -0.7719903 -0.3768885

H 0.1386050 2.7792311 -2.6400022

H 3.0153478 2.6073614 -1.4757316

C 3.3354323 0.5422738 -0.9180728

H -0.3951063 3.2631671 0.3468907

H -3.2650683 2.6741194 -0.6886506

C -3.5425612 0.5319633 -0.5580383

C 3.6638699 -1.8598193 -0.4011174

C -3.8195582 -1.9149697 -0.2869329

C 4.7457956 0.7203642 -0.9055479

C -4.9523603 0.6641691 -0.6215153

C 5.0393802 -1.6116459 -0.3973923

C 3.0722482 -3.2485029 -0.0994985

C -5.2045753 -1.7120146 -0.3654303

C -3.1995220 -3.3161827 -0.1371693

C 5.6154223 -0.3356649 -0.6474553

H 5.1293858 1.7266391 -1.1026914

C -5.8042110 -0.4379588 -0.5285129

H -5.3638800 1.6728643 -0.7527882

H 5.7145736 -2.4451439 -0.1856708

C 2.1934801 -3.1605844 1.1761986

C 2.2244440 -3.7350774 -1.3038671

C 4.1721787 -4.2990129 0.1543955

H -5.8540326 -2.5858173 -0.3001741

C -2.3157488 -3.6160681 -1.3767405

C -2.3481583 -3.3824810 1.1576698

C -4.2767255 -4.4160624 -0.0462962

C 7.1459850 -0.1803211 -0.6118570

C -7.3311966 -0.2456245 -0.6114387

H 2.8211936 -2.9085109 2.0500607

H 1.4163159 -2.3879754 1.0811162

H 1.7082720 -4.1360511 1.3678481

H 2.8657530 -3.8555752 -2.1961635

H 1.7715420 -4.7171229 -1.0718561

H 1.4212796 -3.0225821 -1.5425097

H 3.7017184 -5.2759256 0.3655526

H 4.8306836 -4.4250390 -0.7239850

H 4.8009588 -4.0344237 1.0234176

H -2.9434047 -3.6634116 -2.2850120

H -1.5541892 -2.8370987 -1.5303842

H -1.8104189 -4.5923634 -1.2530868

H -2.9908549 -3.2260371 2.0433248

H -1.8769681 -4.3792232 1.2472995

H -1.5588086 -2.6164551 1.1576507

H -3.7851370 -5.3997057 0.0588185

H -4.9366361 -4.2740253 0.8286135

H -4.9068511 -4.4509538 -0.9531010

C 7.5875411 1.2643843 -0.9157534

C 7.6676915 -0.5637490 0.7951809

C 7.7851859 -1.1168142 -1.6666225

C -7.7951900 0.6939409 0.5285819

C -7.6948558 0.3885945 -1.9765096

C -8.0946595 -1.5784290 -0.4792778

H 7.2579746 1.5858976 -1.9195397

H 7.1827681 1.9764728 -0.1750428

H 8.6896062 1.3326693 -0.8831242

H 7.4059960 -1.6049091 1.0515876

H 8.7685969 -0.4685531 0.8394188

H 7.2297219 0.0962482 1.5646851

H 8.8872086 -1.0278377 -1.6462603

H 7.5267696 -2.1732386 -1.4781096

H 7.4342841 -0.8563179 -2.6808316

H -7.5501329 0.2600194 1.5142545

H -7.3055914 1.6809097 0.4605749

H -8.8886985 0.8522090 0.4804203

H -7.3735277 -0.2652347 -2.8065158

H -8.7875008 0.5406849 -2.0556305

H -7.2042412 1.3687152 -2.1068676

H -9.1816094 -1.3915914 -0.5408345

H -7.8275438 -2.2823601 -1.2870711

H -7.8907184 -2.0700143 0.4882777

H 1.1391041 3.7913425 -1.5350499

H -1.4115220 3.8509722 -1.0199943

Cl -0.5272838 -0.2816139 -3.0204925

**Cat4**Cl.xyz

85

Energy = -3276.341659425

Ti -0.0975617 0.0824904 -0.6943364

N 1.1819501 1.7139476 -1.2643318

N -1.4093866 1.7872955 -0.6397318

O 1.4417516 -0.9337413 -0.7947021

O -1.6139868 -0.8823202 -0.2800601

Cl 0.4165779 0.6988871 1.5098869

C 0.4705701 2.9407320 -1.6284112

C 2.4798220 1.6835727 -1.2535156

C -0.7217227 3.0797151 -0.6767299

C -2.7057371 1.7321862 -0.6225807

C 2.7579046 -0.7648689 -0.7320156

C -2.9356207 -0.7657821 -0.3592064

H 0.1016418 2.8165426 -2.6623114

H 3.0251769 2.6115126 -1.4977887

C 3.3078188 0.5335258 -0.9510002

H -0.3528718 3.2873890 0.3437040

H -3.2692005 2.6800869 -0.6681319

C -3.5116867 0.5296648 -0.5402061

C 3.6126486 -1.8708011 -0.4576156

C -3.7678476 -1.9125130 -0.2551431

C 4.7113855 0.7178793 -0.9049502

C -4.9157059 0.6630713 -0.6088641

C 4.9899480 -1.6164404 -0.4176765

C 3.0257350 -3.2701800 -0.1965463

C -5.1560754 -1.7095398 -0.3400669

C -3.1539619 -3.3132050 -0.0743689

C 5.5745991 -0.3441189 -0.6353432

H 5.0986174 1.7251122 -1.0824987

C -5.7631958 -0.4463034 -0.5156400

H -5.3293347 1.6689380 -0.7431642

H 5.6580792 -2.4537165 -0.2022383

C 2.1181460 -3.2195835 1.0604925

C 2.2110546 -3.7401134 -1.4296101

C 4.1327695 -4.3125800 0.0611984

H -5.8011640 -2.5851364 -0.2660860

C -2.2620895 -3.6469938 -1.2990094

C -2.3146478 -3.3556750 1.2291586

C -4.2403483 -4.4031011 0.0299640

C 7.1028981 -0.1901573 -0.5613118

C -7.2891056 -0.2643676 -0.6078000

H 2.7110217 -2.9421110 1.9498908

H 1.3063881 -2.4864433 0.9472113

H 1.6712405 -4.2144632 1.2385198

H 2.8646782 -3.8131906 -2.3171638

H 1.7846569 -4.7405744 -1.2336237

H 1.3869500 -3.0484105 -1.6582675

H 3.6672710 -5.2972990 0.2400732

H 4.8116306 -4.4139699 -0.8041638

H 4.7363411 -4.0611595 0.9512377

H -2.8705262 -3.6725000 -2.2202945

H -1.4633748 -2.9033773 -1.4358119

H -1.7976903 -4.6404881 -1.1629552

H -2.9569123 -3.1592166 2.1061786

H -1.8654241 -4.3581399 1.3497691

H -1.5056181 -2.6104279 1.2149166

H -3.7546638 -5.3856328 0.1606961

H -4.9055349 -4.2392308 0.8963767

H -4.8609417 -4.4550200 -0.8820781

C 7.5482005 1.2593111 -0.8351238

C 7.5859754 -0.5946252 0.8537363

C 7.7618677 -1.1145255 -1.6148455

C -7.7590947 0.6829437 0.5236187

C -7.6426938 0.3580319 -1.9812447

C -8.0427342 -1.6017223 -0.4690327

H 7.2454718 1.5937884 -1.8430336

H 7.1263630 1.9612075 -0.0942978

H 8.6483887 1.3266099 -0.7740455

H 7.3227254 -1.6405723 1.0879759

H 8.6844162 -0.4974654 0.9242969

H 7.1292489 0.0534045 1.6222298

H 8.8619583 -1.0225457 -1.5680534

H 7.5039146 -2.1739648 -1.4435635

H 7.4334545 -0.8425109 -2.6333567

H -7.5159022 0.2596808 1.5140944

H -7.2787465 1.6738350 0.4477736

H -8.8526797 0.8312730 0.4680493

H -7.3153105 -0.3009136 -2.8045602

H -8.7348870 0.5035578 -2.0654841

H -7.1577997 1.3403563 -2.1162345

H -9.1294916 -1.4211488 -0.5387154

H -7.7681747 -2.3099831 -1.2704597

H -7.8434217 -2.0832710 0.5044532

H 1.1307578 3.8243070 -1.5813974

H -1.3986522 3.8929941 -0.9912973

Cl -0.6170458 -0.0058465 -2.9796260

**Cat4**_OC_2_H_4_.xyz

91

Energy = -2969.935223832

Ti 0.0416066 0.3608705 -0.4133507

C -0.5310524 3.3077432 0.1233748

C 0.7205040 3.2744468 -0.7482839

N -1.2065410 2.0131322 -0.0248873

N 1.3747948 1.9886521 -0.5070694

Cl -0.3255650 0.2963873 -2.7260074

O -1.4992012 -0.6578321 -0.0216919

O 1.6117470 -0.6670427 -0.3743834

C -2.5165381 1.9841671 0.1308340

H -3.0345454 2.9436095 0.2993864

C 2.6819803 1.9569177 -0.3711963

H 3.2278974 2.9156048 -0.4014963

C -3.3570707 0.8176671 0.1085987

C -4.7649618 0.9910756 0.2117981

C -2.8146513 -0.5035319 0.0021450

C -5.6343498 -0.0937364 0.1798239

H -5.1436935 2.0129677 0.3089837

C -3.6886143 -1.6310002 -0.0715356

C -5.0621393 -1.3851938 0.0243469

H -5.7418837 -2.2390216 -0.0298193

C 3.4913496 0.7778972 -0.2086434

C 4.8957877 0.9282617 -0.0512449

C 2.9218208 -0.5345634 -0.2513062

C 5.7378509 -0.1748616 0.0514369

H 5.2958959 1.9460510 -0.0171625

C 3.7691578 -1.6836681 -0.1880956

C 5.1411245 -1.4619517 -0.0306847

H 5.7989794 -2.3325477 0.0278296

C -3.1262506 -3.0451271 -0.3095075

C -2.4049437 -3.0698020 -1.6829050

C -2.1329060 -3.4384282 0.8122589

C -4.2438721 -4.1077228 -0.3370618

H -1.9486843 -4.0629477 -1.8508126

H -3.1284941 -2.8803891 -2.4957258

H -1.6163557 -2.3037871 -1.7427282

H -1.7884374 -4.4772791 0.6599673

H -1.2533889 -2.7792544 0.8043794

H -2.6163678 -3.3767153 1.8036625

H -3.7957471 -5.1004049 -0.5178180

H -4.7909533 -4.1537475 0.6215962

H -4.9727616 -3.9178239 -1.1445718

C 3.1764012 -3.0991609 -0.3208607

C 2.1523469 -3.3595673 0.8121783

C 2.4798834 -3.2325406 -1.7009442

C 4.2660390 -4.1865472 -0.2302647

H 1.7497924 -4.3853007 0.7272012

H 2.6324416 -3.2572039 1.8018651

H 1.3140758 -2.6517517 0.7519290

H 2.0215854 -4.2341873 -1.7947619

H 1.6931876 -2.4733516 -1.8315115

H 3.2186253 -3.1155656 -2.5139821

H 5.0195114 -4.0815432 -1.0307804

H 4.7876724 -4.1651623 0.7432010

H 3.7971340 -5.1801439 -0.3389089

C 7.2608910 -0.0604937 0.2354664

C 7.7251032 1.4075035 0.2954114

C 7.6682091 -0.7578457 1.5570409

C 7.9760307 -0.7520145 -0.9516125

H 7.4814069 1.9488765 -0.6357148

H 7.2611453 1.9457676 1.1407002

H 8.8202139 1.4457319 0.4309824

H 7.3875681 -1.8254230 1.5540692

H 8.7619515 -0.6950984 1.7032586

H 7.1737005 -0.2773386 2.4196252

H 9.0726182 -0.6868659 -0.8298805

H 7.7064292 -1.8201352 -1.0209165

H 7.7031401 -0.2683392 -1.9060559

C -7.1624679 0.0447661 0.2888115

C -7.5975122 1.5136907 0.4529613

C -7.8217456 -0.5210059 -0.9934094

C -7.6611648 -0.7542067 1.5182616

H -7.1700549 1.9636944 1.3663966

H -7.2902333 2.1272854 -0.4121689

H -8.6974651 1.5692376 0.5319653

H -7.5717126 -1.5858782 -1.1412749

H -8.9219229 -0.4371026 -0.9287021

H -7.4818751 0.0360526 -1.8841828

H -8.7600481 -0.6760408 1.6075220

H -7.4033445 -1.8244619 1.4370127

H -7.2081526 -0.3637145 2.4466277

O 0.3917484 0.4434156 1.8065799

C 0.1695978 -0.6731800 2.7065118

C -0.6542023 0.5404787 2.8058212

H 0.9941381 -0.8429313 3.4085809

H -0.2584507 -1.5593413 2.2254042

H -0.4490181 1.2821507 3.5869863

H -1.6715189 0.5278867 2.3962171

H 0.4293899 3.3163100 -1.8145465

H 1.3905804 4.1257726 -0.5278096

H -0.2354877 3.4401206 1.1822045

H -1.1987179 4.1440905 -0.1532449

**Cat4**.xyz

84

Energy = -2816.195883802

Ti -0.1035187 0.3194458 0.3790474

N 1.0909009 1.8200100 -0.4597638

N -1.4606127 1.8820801 -0.0011864

O 1.4035224 -0.7409980 0.1254950

O -1.5801043 -0.7609043 0.0235704

Cl -0.0638208 0.6447575 2.6528782

C 0.4359062 3.1045704 -0.7762219

C 2.3683046 1.7196943 -0.8068886

C -0.8253596 3.1989733 0.0729873

C -2.7486242 1.8036797 -0.2399196

C 2.6914704 -0.6766115 -0.1905769

C -2.8843188 -0.6981505 -0.2465946

H 0.1630075 3.1196346 -1.8485379

H 2.8453432 2.6099178 -1.2481436

C 3.2063998 0.5640517 -0.6787655

H -0.5589876 3.3919075 1.1285729

H -3.3116543 2.7431273 -0.3692329

C -3.5062280 0.5840359 -0.3688631

C 3.5465020 -1.8090527 -0.0460440

C -3.6543677 -1.8835337 -0.4066371

C 4.5745951 0.6522421 -1.0604555

C -4.8924212 0.6658775 -0.6438304

C 4.8827571 -1.6486283 -0.4298395

C 3.0123711 -3.1283890 0.5409169

C -5.0261778 -1.7306578 -0.6687195

C -2.9955576 -3.2723877 -0.3069661

C 5.4273212 -0.4407746 -0.9467138

H 4.9353981 1.6120036 -1.4414831

C -5.6764803 -0.4803484 -0.7923770

H -5.3378779 1.6630368 -0.7368609

H 5.5535194 -2.5044380 -0.3230376

C 2.5131940 -2.8769741 1.9879517

C 1.8533755 -3.6693661 -0.3332340

C 4.1077322 -4.2123201 0.5982158

H -5.6237346 -2.6348751 -0.7841262

C -1.9227789 -3.4072131 -1.4177216

C -2.3480755 -3.4549520 1.0900340

C -4.0205191 -4.4084628 -0.5012938

C 6.9135549 -0.3948148 -1.3415348

C -7.1842671 -0.3559535 -1.0815643

H 3.3503947 -2.5496680 2.6301093

H 1.7291346 -2.1049159 2.0206535

H 2.1022718 -3.8123336 2.4098340

H 2.2006453 -3.8576877 -1.3648815

H 1.4833988 -4.6231861 0.0847875

H 1.0151145 -2.9596552 -0.3697777

H 3.6808884 -5.1361421 1.0259969

H 4.4975610 -4.4552483 -0.4062642

H 4.9557788 -3.9077933 1.2367288

H -2.3906727 -3.3275616 -2.4151209

H -1.1542813 -2.6254678 -1.3271098

H -1.4269167 -4.3920081 -1.3459459

H -3.1182747 -3.3985567 1.8798202

H -1.8653207 -4.4472792 1.1503995

H -1.5879867 -2.6849065 1.2878734

H -3.5020560 -5.3799348 -0.4233540

H -4.8101890 -4.3861932 0.2704950

H -4.5019601 -4.3641726 -1.4942739

C 7.3218783 0.9871746 -1.8868937

C 7.7823258 -0.7082126 -0.0980796

C 7.1838971 -1.4542723 -2.4384003

C -7.8614471 0.4230208 0.0728708

C -7.3862340 0.4139868 -2.4099600

C -7.8675669 -1.7319919 -1.2074395

H 6.7446860 1.2538718 -2.7898499

H 7.1745166 1.7810264 -1.1336218

H 8.3913171 0.9767953 -2.1607755

H 7.5548714 -1.7063886 0.3146617

H 8.8543905 -0.6883718 -0.3653653

H 7.6084489 0.0379285 0.6971328

H 8.2503059 -1.4418645 -2.7278950

H 6.9404334 -2.4722965 -2.0881214

H 6.5771840 -1.2481024 -3.3377024

H -7.7292717 -0.1075522 1.0322187

H -7.4356664 1.4356450 0.1809794

H -8.9445942 0.5286691 -0.1194412

H -6.9107498 -0.1240391 -3.2489514

H -8.4636926 0.5201821 -2.6316723

H -6.9485509 1.4260990 -2.3608957

H -8.9432886 -1.5923056 -1.4124122

H -7.4418877 -2.3251017 -2.0360249

H -7.7760209 -2.3195383 -0.2769621

H 1.1190845 3.9509891 -0.5843287

H -1.4958734 4.0024730 -0.2813919

**Cat6**Cl^−^.xyz

105

Energy = -3738.361494706

Ti -0.0903059 0.2510997 -0.2866075

N 1.2218085 1.8816794 -0.4494880

N -1.4246324 1.8640750 -0.1240517

O 1.4571031 -0.8111451 -0.4352598

O -1.6227516 -0.8315397 -0.1443255

Cl 0.2232175 0.3841788 2.1250539

C 0.5947826 3.2011698 -0.6041165

C 2.5348940 1.8097987 -0.5411257

C -0.8115982 3.1862323 0.0611726

C -2.7378591 1.7761331 -0.0569590

C 2.7643305 -0.6856942 -0.4353078

C -2.9321193 -0.7253320 -0.1837840

H 0.4439391 3.3619885 -1.6882263

C 1.3954744 4.3541161 -0.0229048

H 3.0964067 2.7523970 -0.6477791

C 3.3428417 0.6224987 -0.4996193

H -0.6612711 3.3193616 1.1491893

C -1.6222570 4.3471360 -0.4889926

H -3.3135523 2.7099838 0.0496986

C -3.5292776 0.5772014 -0.1271409

C 3.6090140 -1.8385472 -0.3770006

C -3.7584268 -1.8848132 -0.2767036

C 1.6341982 5.5212845 -0.7649365

C 1.8529772 4.2790491 1.3089901

C 4.7575337 0.7571926 -0.5287377

C -1.8730594 5.4901138 0.2860299

C -2.0753031 4.3077803 -1.8239622

C -4.9422055 0.6885952 -0.1343861

C 4.9911323 -1.6328982 -0.4054384

C 2.9807204 -3.2389977 -0.2655476

C -5.1482394 -1.7010782 -0.2855778

C -3.1091584 -3.2758847 -0.3899422

C 2.3198394 6.6033523 -0.1901219

H 1.2716592 5.5840741 -1.7970789

C 2.5412000 5.3551906 1.8818725

H 1.6726848 3.3602055 1.8806248

C 5.5995877 -0.3497647 -0.4848127

H 5.1668076 1.7712207 -0.5781545

C -2.5653512 6.5832070 -0.2590538

H -1.5139946 5.5256746 1.3206555

C -2.7706003 5.3946277 -2.3673661

H -1.8863784 3.4080445 -2.4224240

C -5.7725783 -0.4303801 -0.2111444

H -5.3730288 1.6966188 -0.0878160

H 5.6453819 -2.5079666 -0.3606252

C 2.1525404 -3.3222007 1.0438305

C 2.0703574 -3.5074997 -1.4920061

C 4.0511476 -4.3479165 -0.2211418

H -5.7812561 -2.5864630 -0.3575049

C -2.2520029 -3.3307116 -1.6820699

C -2.2212717 -3.5490249 0.8518245

C -4.1628926 -4.3988055 -0.4713369

C 2.7751782 6.5218871 1.1334833

H 2.4987943 7.5102410 -0.7783308

H 2.8979953 5.2864179 2.9155703

C 7.1345642 -0.2439202 -0.5080791

C -3.0158476 6.5371116 -1.5860250

H -2.7529020 7.4711306 0.3548249

H -3.1235385 5.3532625 -3.4038183

C -7.3040800 -0.2575889 -0.2239626

H 2.8192521 -3.2224049 1.9195634

H 1.3975071 -2.5235431 1.0946918

H 1.6415842 -4.3013478 1.1067590

H 2.6694210 -3.4937942 -2.4207723

H 1.5986916 -4.5037651 -1.3997950

H 1.2790288 -2.7483431 -1.5753541

H 3.5547613 -5.3312686 -0.1376986

H 4.6692682 -4.3589446 -1.1369937

H 4.7234299 -4.2357788 0.6483907

H -2.9012562 -3.2287617 -2.5705304

H -1.5083309 -2.5202854 -1.7071833

H -1.7252599 -4.3013782 -1.7467381

H -2.8385499 -3.5491303 1.7686798

H -1.7397005 -4.5405543 0.7594316

H -1.4381550 -2.7842018 0.9578859

H -3.6508947 -5.3741106 -0.5550110

H -4.8012284 -4.4294247 0.4301213

H -4.8170078 -4.2860395 -1.3545333

H 3.3134473 7.3639045 1.5827739

C 7.6117711 1.2185322 -0.5983449

C 7.7128074 -0.8653547 0.7872881

C 7.6855747 -1.0124330 -1.7344729

H -3.5589824 7.3878585 -2.0123852

C -7.7525333 0.4551219 1.0754791

C -7.7141289 0.6008278 -1.4457587

C -8.0426796 -1.6078795 -0.3129983

H 7.2454413 1.7083834 -1.5178986

H 7.2686262 1.8105583 0.2684091

H 8.7158303 1.2511185 -0.6145107

H 7.4284295 -1.9273403 0.8865132

H 8.8172112 -0.8076562 0.7858455

H 7.3364949 -0.3284654 1.6759073

H 8.7897931 -0.9582487 -1.7620700

H 7.3987287 -2.0779226 -1.7045402

H 7.2921486 -0.5808012 -2.6717879

H -7.4722606 -0.1406261 1.9622365

H -7.2816318 1.4488191 1.1716185

H -8.8494992 0.5959878 1.0830699

H -7.4037219 0.1112400 -2.3856718

H -8.8107609 0.7423342 -1.4712673

H -7.2424836 1.5981184 -1.4121369

H -9.1337610 -1.4353456 -0.3160264

H -7.7847049 -2.1526781 -1.2382489

H -7.8061862 -2.2584861 0.5474042

Cl -0.4056332 0.3786686 -2.6962687

**Cat6**Cl.xyz

105

Energy = -3738.260088229

Ti -0.0886744 0.1995684 -0.2977374

N 1.2284692 1.8772462 -0.5178167

N -1.4273169 1.8597316 -0.0629348

O 1.4257394 -0.8289196 -0.4922013

O -1.5901109 -0.8483043 -0.1201954

Cl 0.3074163 0.5396513 1.9939838

C 0.5597746 3.1839787 -0.6798126

C 2.5240796 1.7967843 -0.5774947

C -0.7719380 3.1698135 0.1241595

C -2.7217063 1.7642306 -0.0182461

C 2.7486480 -0.7094835 -0.4532203

C -2.9145551 -0.7462374 -0.1798138

H 0.2978500 3.2547454 -1.7505959

C 1.3899833 4.3840599 -0.2703786

H 3.0968880 2.7315156 -0.6902481

C 3.3252158 0.5928405 -0.5000765

H -0.5099663 3.2200813 1.1960440

C -1.6141769 4.3699573 -0.2588484

H -3.3065306 2.6909076 0.1000038

C -3.5074511 0.5510245 -0.1223608

C 3.5778802 -1.8640431 -0.3737004

C -3.7279747 -1.9049534 -0.2915581

C 1.6025929 5.4456531 -1.1641981

C 1.9113916 4.4661744 1.0368620

C 4.7340649 0.7354980 -0.4847800

C -1.8425851 5.4060688 0.6606982

C -2.1316876 4.4792544 -1.5656746

C -4.9135518 0.6737207 -0.1555606

C 4.9628608 -1.6506232 -0.3548442

C 2.9596394 -3.2717585 -0.2949596

C -5.1197949 -1.7104517 -0.3276504

C -3.0930873 -3.3043325 -0.3891543

C 2.3244407 6.5791935 -0.7605399

H 1.1914083 5.3865040 -2.1777045

C 2.6368864 5.5943267 1.4383976

H 1.7568168 3.6341807 1.7345929

C 5.5757984 -0.3742184 -0.4102394

H 5.1440665 1.7483451 -0.5272961

C -2.5755783 6.5413125 0.2828254

H -1.4348190 5.3258618 1.6741311

C -2.8683323 5.6090281 -1.9416051

H -1.9649343 3.6671868 -2.2838061

C -5.7456783 -0.4460142 -0.2601788

H -5.3418606 1.6811394 -0.1044744

H 5.6135891 -2.5261376 -0.2923929

C 2.0906078 -3.3788468 0.9855080

C 2.0956337 -3.5392389 -1.5545687

C 4.0434096 -4.3666446 -0.2248862

H -5.7522048 -2.5941145 -0.4139378

C -2.2086133 -3.3763627 -1.6615333

C -2.2397823 -3.5871361 0.8743704

C -4.1628407 -4.4104732 -0.4930015

C 2.8424147 6.6549073 0.5402586

H 2.4822837 7.4034456 -1.4641200

H 3.0418728 5.6481024 2.4544431

C 7.1096566 -0.2675757 -0.3825406

C -3.0891955 6.6441964 -1.0178308

H -2.7453995 7.3456760 1.0063368

H -3.2697842 5.6842351 -2.9576837

C -7.2746174 -0.2724263 -0.3034556

H 2.7174099 -3.2506457 1.8856600

H 1.2997248 -2.6146882 1.0055025

H 1.6163334 -4.3758143 1.0328399

H 2.7190189 -3.4951206 -2.4653822

H 1.6493192 -4.5481394 -1.4917556

H 1.2824252 -2.8050014 -1.6515228

H 3.5551086 -5.3548826 -0.1685968

H 4.6909925 -4.3622263 -1.1195868

H 4.6821791 -4.2571531 0.6693266

H -2.8268102 -3.2379511 -2.5661073

H -1.4272574 -2.6022981 -1.6578476

H -1.7215321 -4.3663944 -1.7229630

H -2.8731277 -3.5667417 1.7791477

H -1.7824764 -4.5901247 0.7971466

H -1.4353267 -2.8465913 0.9939225

H -3.6618715 -5.3915508 -0.5621226

H -4.8210036 -4.4308896 0.3937084

H -4.7921720 -4.2917052 -1.3926939

H 3.4079000 7.5382705 0.8555376

C 7.5850217 1.1965789 -0.4513426

C 7.6398178 -0.8962548 0.9299827

C 7.6954755 -1.0328008 -1.5949961

H -3.6631676 7.5289868 -1.3131097

C -7.7432325 0.4346069 0.9922989

C -7.6523533 0.5945425 -1.5300181

C -8.0089831 -1.6229505 -0.4153286

H 7.2515765 1.6894549 -1.3816705

H 7.2155972 1.7847891 0.4072611

H 8.6881213 1.2291900 -0.4304982

H 7.3575398 -1.9601106 1.0129810

H 8.7424641 -0.8349563 0.9638825

H 7.2343317 -0.3652737 1.8090205

H 8.7987534 -0.9745012 -1.5858332

H 7.4131880 -2.0996844 -1.5761271

H 7.3321703 -0.5991410 -2.5431885

H -7.4824971 -0.1657148 1.8815753

H -7.2771187 1.4290864 1.1028402

H -8.8391302 0.5744727 0.9761128

H -7.3245456 0.1104776 -2.4666185

H -8.7472500 0.7358553 -1.5754884

H -7.1841719 1.5929018 -1.4798382

H -9.0986472 -1.4486164 -0.4402318

H -7.7338072 -2.1620506 -1.3388736

H -7.7928490 -2.2773958 0.4474019

Cl -0.4914996 0.5586220 -2.5860669

**Cat6**_OC_2_H_4_.xyz

111

Energy = -3431.848443101

Ti 0.0515004 0.5097936 -0.3401081

C -0.5976056 3.4320288 0.3636301

C 0.7870266 3.4259035 -0.3312718

N -1.2009264 2.0892390 0.2247813

N 1.4038194 2.1023979 -0.1296626

Cl -0.2262835 0.8777838 -2.6213072

O -1.4801293 -0.5633168 -0.1231217

O 1.5881571 -0.5694741 -0.4006518

C -2.4922988 2.0005304 0.5036609

H -3.0152515 2.9244996 0.7974702

C 2.7144571 2.0080234 -0.0469753

H 3.2961574 2.9418781 0.0073318

C -3.3153919 0.8270430 0.4385952

C -4.7030845 0.9511192 0.7256532

C -2.7844962 -0.4494792 0.0696948

C -5.5648684 -0.1350450 0.6240568

H -5.0731253 1.9378187 1.0191137

C -3.6563007 -1.5678584 -0.0949167

C -5.0104196 -1.3724890 0.1967540

H -5.6891288 -2.2211006 0.0809810

C 3.4922359 0.7986710 -0.0390798

C 4.9002738 0.9001500 0.1285640

C 2.8980346 -0.4852847 -0.2522576

C 5.7214439 -0.2220926 0.0891891

H 5.3201336 1.8976234 0.2900209

C 3.7221045 -1.6510340 -0.3235984

C 5.0984864 -1.4779555 -0.1449970

H 5.7382890 -2.3626568 -0.1925832

C -3.1172713 -2.9051140 -0.6353921

C -2.5432291 -2.6689267 -2.0575877

C -2.0088933 -3.4664798 0.2895694

C -4.2281703 -3.9698510 -0.7367763

H -2.1108084 -3.6082089 -2.4494718

H -3.3471454 -2.3466401 -2.7431312

H -1.7617887 -1.8932581 -2.0566121

H -1.6773427 -4.4543436 -0.0783170

H -1.1368117 -2.7976947 0.3072297

H -2.3856923 -3.5921102 1.3205810

H -3.8000049 -4.9042025 -1.1401447

H -4.6687058 -4.1989951 0.2500542

H -5.0404214 -3.6534159 -1.4146483

C 3.0976924 -3.0305597 -0.6028179

C 2.0912756 -3.3866877 0.5198973

C 2.3712689 -3.0012686 -1.9731216

C 4.1640041 -4.1431590 -0.6534666

H 1.6419715 -4.3774338 0.3251323

H 2.6004176 -3.4232582 1.4995960

H 1.2836638 -2.6430948 0.5703864

H 1.8987256 -3.9816514 -2.1677268

H 1.5929246 -2.2236900 -1.9998196

H 3.0940827 -2.8015587 -2.7844485

H 4.9052148 -3.9656462 -1.4528336

H 4.7035470 -4.2398296 0.3055200

H 3.6711812 -5.1092259 -0.8607008

C 7.2469807 -0.1592493 0.2767534

C 7.7394010 1.2816054 0.5125913

C 7.6466460 -1.0201967 1.5006379

C 7.9437556 -0.7132169 -0.9906515

H 7.5015290 1.9373454 -0.3435764

H 7.2903980 1.7202406 1.4211959

H 8.8357451 1.2834288 0.6436030

H 7.3454090 -2.0740703 1.3695146

H 8.7419993 -0.9960906 1.6463610

H 7.1649466 -0.6395451 2.4185655

H 9.0419267 -0.6842828 -0.8691086

H 7.6527804 -1.7594367 -1.1885026

H 7.6762524 -0.1109302 -1.8766906

C -7.0702425 -0.0486565 0.9284924

C -7.4883106 1.3675404 1.3688505

C -7.8742319 -0.4228543 -0.3412295

C -7.4177869 -1.0387736 2.0674789

H -6.9566116 1.6815097 2.2844295

H -7.2889122 2.1132547 0.5790831

H -8.5711749 1.3859894 1.5837856

H -7.6409948 -1.4466951 -0.6815132

H -8.9594196 -0.3727186 -0.1375072

H -7.6424190 0.2721640 -1.1673810

H -8.4988183 -1.0001237 2.2946718

H -7.1665258 -2.0773608 1.7906163

H -6.8597513 -0.7863406 2.9863853

O 0.3912125 0.2493211 1.8877119

C 0.0423244 -0.9384523 2.6423089

C -0.6054760 0.3495120 2.9332337

H 0.8525890 -1.3080570 3.2815532

H -0.5120027 -1.6877941 2.0662626

H -0.2822686 0.9453748 3.7954485

H -1.6270213 0.5249558 2.5743068

H 0.6011079 3.4954552 -1.4198191

H -0.4388341 3.6305555 1.4407211

C -1.4491658 4.5431666 -0.2273844

C -1.6769888 5.7336019 0.4804916

C -1.9631387 4.4032755 -1.5327783

C -2.4126301 6.7772020 -0.1029810

H -1.2687082 5.8459323 1.4911047

C -2.6999284 5.4430337 -2.1127576

H -1.7844771 3.4705308 -2.0819938

C -2.9260308 6.6329214 -1.3996503

H -2.5854431 7.7023356 0.4575441

H -3.1005525 5.3272690 -3.1258077

H -3.5029144 7.4449891 -1.8555713

C 1.5988251 4.6299380 0.1075113

C 1.8805723 5.6611715 -0.8038900

C 2.0341932 4.7608299 1.4417637

C 2.5817353 6.8053929 -0.3944950

H 1.5379268 5.5676924 -1.8403179

C 2.7412307 5.8980799 1.8519400

H 1.8265672 3.9604374 2.1613375

C 3.0146548 6.9253135 0.9337965

H 2.7903517 7.6035070 -1.1149792

H 3.0784535 5.9851507 2.8903730

H 3.5649123 7.8162826 1.2548653

**Cat6**.xyz

104

Energy = -3278.110491739

Ti -0.1049355 0.3948454 0.6486531

N 1.0898121 1.8926717 -0.1477998

N -1.4616383 1.9415124 0.2150312

O 1.3945077 -0.6727706 0.3885905

O -1.5499394 -0.7142840 0.2296107

Cl -0.0789702 0.8120909 2.8960736

C 0.5171194 3.2567357 -0.2724396

C 2.3254135 1.7601467 -0.6239873

C -0.8506320 3.2669740 0.4476429

C -2.7466238 1.8366228 -0.0334062

C 2.6501560 -0.6398653 -0.0411443

C -2.8337523 -0.6670814 -0.1231859

H 0.3496037 3.4691631 -1.3443678

C 1.4204599 4.3282282 0.3134497

H 2.7946663 2.6536405 -1.0647028

C 3.1353798 0.5815621 -0.6010815

H -0.6519668 3.3034912 1.5350138

C -1.6754299 4.4795624 0.0648743

H -3.3370498 2.7620317 -0.1200265

C -3.4708669 0.6079867 -0.2352702

C 3.5004754 -1.7792596 0.0596855

C -3.5717231 -1.8573812 -0.3748417

C 1.6792250 5.5146505 -0.3914470

C 1.9614251 4.1564855 1.6035048

C 4.4598854 0.6360112 -1.1185236

C -2.0765594 5.3915467 1.0562412

C -2.0336733 4.7292552 -1.2757197

C -4.8445235 0.6767648 -0.5708837

C 4.7931413 -1.6535280 -0.4613034

C 3.0125225 -3.0646216 0.7519510

C -4.9318515 -1.7175070 -0.6980440

C -2.8891433 -3.2364908 -0.3129254

C 2.4724354 6.5215316 0.1807232

H 1.2530548 5.6517917 -1.3915939

C 2.7552042 5.1597583 2.1724461

H 1.7593897 3.2289023 2.1522493

C 5.3005180 -0.4700412 -1.0652317

H 4.7987521 1.5820694 -1.5502505

C -2.8161723 6.5350235 0.7199497

H -1.7990829 5.2046000 2.0995759

C -2.7788139 5.8662833 -1.6135913

H -1.7360369 4.0259005 -2.0616466

C -5.6002248 -0.4746467 -0.8001877

H -5.3027631 1.6695062 -0.6492986

H 5.4592554 -2.5171636 -0.3955842

C 2.6582755 -2.7394723 2.2270580

C 1.7674858 -3.6262103 0.0218504

C 4.0976741 -4.1600665 0.7524642

H -5.5052100 -2.6262663 -0.8809865

C -1.7687554 -3.2898535 -1.3829686

C -2.2968593 -3.4774825 1.0992230

C -3.8794518 -4.3813669 -0.6084729

C 3.0123322 6.3451149 1.4625952

H 2.6700640 7.4429421 -0.3776294

H 3.1762517 5.0187097 3.1738680

C 6.7396842 -0.4608151 -1.6085199

C -3.1695604 6.7739626 -0.6158681

H -3.1163947 7.2393706 1.5029851

H -3.0531052 6.0465333 -2.6583995

C -7.0954184 -0.3637448 -1.1529990

H 3.5550633 -2.3866808 2.7670226

H 1.8838247 -1.9598674 2.2962650

H 2.2874664 -3.6500282 2.7325621

H 2.0049970 -3.8566656 -1.0320670

H 1.4339804 -4.5590615 0.5119147

H 0.9360565 -2.9084459 0.0430533

H 3.7067396 -5.0574976 1.2629881

H 4.3841017 -4.4541639 -0.2730161

H 5.0077202 -3.8381671 1.2890927

H -2.1985486 -3.1720787 -2.3936391

H -1.0257245 -2.4945758 -1.2226958

H -1.2492685 -4.2639971 -1.3390664

H -3.0995564 -3.4706219 1.8580928

H -1.8003581 -4.4643221 1.1321162

H -1.5590689 -2.7061709 1.3635922

H -3.3435303 -5.3450730 -0.5561119

H -4.7019870 -4.4138077 0.1279335

H -4.3187995 -4.2966276 -1.6181873

H 3.6343058 7.1284095 1.9093165

C 7.1157250 0.9023772 -2.2204081

C 7.7251195 -0.7675372 -0.4536421

C 6.8779702 -1.5457972 -2.7050975

H -3.7488929 7.6648734 -0.8807613

C -7.8380252 0.3569488 -0.0009116

C -7.2524147 0.4547842 -2.4581546

C -7.7468239 -1.7448761 -1.3633193

H 6.4536012 1.1653654 -3.0641646

H 7.0620685 1.7120565 -1.4713727

H 8.1508836 0.8654543 -2.6025131

H 7.5245071 -1.7537008 -0.0003015

H 8.7648954 -0.7720601 -0.8281338

H 7.6442630 -0.0040763 0.3399731

H 7.9097250 -1.5617592 -3.1009238

H 6.6511455 -2.5513951 -2.3104408

H 6.1871994 -1.3440080 -3.5426739

H -7.7387855 -0.2090913 0.9419596

H -7.4366540 1.3715144 0.1667292

H -8.9132051 0.4523934 -0.2378275

H -6.7289483 -0.0403767 -3.2948662

H -8.3205546 0.5513310 -2.7245215

H -6.8372968 1.4719395 -2.3501746

H -8.8148827 -1.6152169 -1.6105868

H -7.2739690 -2.2969038 -2.1946615

H -7.6852258 -2.3675327 -0.4534717

**Cat7**Cl^−^.xyz

97

Energy = -3433.619748888

Ti -0.0021288 0.0001887 0.3794261

C 0.0657949 -1.5761850 4.5546540

C 0.4444434 -0.6491145 3.3822352

C -0.4527405 0.6493726 3.3815348

C -0.0766545 1.5762637 4.5548652

H 0.8137785 -2.3771590 4.6810729

H 0.9138893 2.0395152 4.4195921

H -0.8248132 2.3773681 4.6794938

H -0.9243324 -2.0396037 4.4169207

N 0.2430065 -1.2794131 2.0483292

N -0.2486326 1.2797861 2.0481618

Cl -2.3303342 -0.5934033 0.1909783

Cl 2.3252924 0.5959558 0.1953523

O 0.3393972 -1.5409164 -0.6740787

O -0.3425165 1.5414984 -0.6744898

C 0.2510259 -2.5957367 1.9631386

H 0.3247518 -3.1800092 2.8923579

C -0.2534159 2.5959993 1.9630021

H -0.3267346 3.1804942 2.8921199

C 0.1839969 -3.4056630 0.7749081

C 0.1091049 -4.8187993 0.9145328

C 0.2321532 -2.8362137 -0.5384083

C 0.0628719 -5.6672592 -0.1888787

H 0.0830544 -5.2247767 1.9315293

C 0.1837550 -3.6877690 -1.6882611

C 0.0993350 -5.0669593 -1.4781466

H 0.0529349 -5.7232803 -2.3518999

C -0.1829850 3.4057103 0.7747676

C -0.1041410 4.8186424 0.9145234

C -0.2326706 2.8366847 -0.5385324

C -0.0552246 5.6669098 -0.1888997

H -0.0769088 5.2245003 1.9315338

C -0.1835855 3.6881175 -1.6883250

C -0.0945997 5.0668628 -1.4781736

H -0.0474186 5.7231814 -2.3518767

C 0.1903691 -3.0562676 -3.0906598

C -1.0570511 -2.1459557 -3.2393605

C 1.4852547 -2.2252597 -3.2888904

C 0.1383299 -4.1234362 -4.2021709

H -1.0453208 -1.6470536 -4.2272505

H -1.9775006 -2.7529440 -3.1653628

H -1.0958454 -1.3806037 -2.4505087

H 1.4710883 -1.7394487 -4.2830128

H 1.5859208 -1.4500503 -2.5146002

H 2.3703197 -2.8865387 -3.2429784

H 0.1463237 -3.6253437 -5.1883930

H 1.0087387 -4.8032963 -4.1605377

H -0.7799987 -4.7350256 -4.1419784

C -0.1963455 3.0568156 -3.0907133

C 1.0482664 2.1433327 -3.2436212

C -1.4938979 2.2288068 -3.2849480

C -0.1452436 4.1240509 -4.2021847

H 1.0322758 1.6446225 -4.2315393

H 1.9704986 2.7479373 -3.1723041

H 1.0873494 1.3777437 -2.4550243

H -1.4841007 1.7435730 -4.2794178

H -1.5929624 1.4527669 -2.5112229

H -2.3778454 2.8913947 -3.2352037

H -1.0137718 4.8060899 -4.1574750

H 0.7748370 4.7332889 -4.1448391

H -0.1576257 3.6262239 -5.1885000

H -0.0666807 1.0024639 5.4956788

H 0.0534512 -1.0024624 5.4954901

C 1.9412874 -0.2859346 3.4957465

H 2.5431024 -1.1980149 3.3415788

H 2.2264729 0.4363748 2.7137292

H 2.1736689 0.1148294 4.4974938

C -1.9498395 0.2862008 3.4919471

H -2.5513510 1.1982814 3.3365992

H -2.2334777 -0.4360747 2.7093502

H -2.1842553 -0.1146219 4.4931930

C 0.0516577 7.1978900 -0.0730089

C 0.0688663 7.6678824 1.3945163

C 1.3605799 7.6732223 -0.7511108

C -1.1579589 7.8576491 -0.7797141

H -0.8536422 7.3725471 1.9251791

H 0.9310819 7.2502869 1.9439052

H 0.1443826 8.7693472 1.4337786

H 1.3855090 7.3914899 -1.8181033

H 1.4551971 8.7733842 -0.6867781

H 2.2384609 7.2178873 -0.2595779

H -1.0911403 8.9599061 -0.7177062

H -1.2018525 7.5797971 -1.8470749

H -2.1039415 7.5396811 -0.3067095

C -0.0391561 -7.1985786 -0.0729289

C -0.0552713 -7.6685107 1.3946367

C -1.3463760 -7.6782033 -0.7513036

C 1.1727592 -7.8545450 -0.7792292

H 0.8662210 -7.3703781 1.9254972

H -0.9188769 -7.2534849 1.9437941

H -0.1274888 -8.7701929 1.4339506

H -1.3719692 -7.3967412 -1.8183479

H -1.4374450 -8.7786535 -0.6868403

H -2.2258483 -7.2256511 -0.2600508

H 1.1093096 -8.9570021 -0.7172582

H 1.2161467 -7.5765398 -1.8465758

H 2.1176042 -7.5337445 -0.3058498

**Cat7**Cl.xyz

97

Energy = -3433.520966912

Ti 0.0001427 0.0000043 0.3528273

C 0.0185384 -1.5922325 4.5579911

C 0.4402831 -0.6574518 3.4079393

C -0.4390583 0.6573697 3.4081048

C -0.0168977 1.5921183 4.5580261

H 0.7551062 -2.4009457 4.6983331

H 0.9738917 2.0423471 4.3874495

H -0.7534594 2.4007844 4.6986648

H -0.9723646 -2.0423603 4.3878130

N 0.2574552 -1.2856578 2.0642975

N -0.2566843 1.2856199 2.0644040

Cl -2.2643836 -0.5622765 0.3584810

Cl 2.2646813 0.5622655 0.3576821

O 0.3271492 -1.5124880 -0.6736998

O -0.3273557 1.5125012 -0.6734845

C 0.2514094 -2.5859001 1.9574644

H 0.3206813 -3.1846155 2.8780638

C -0.2509845 2.5858741 1.9576339

H -0.3202718 3.1845807 2.8782359

C 0.1748523 -3.3888451 0.7586707

C 0.0931523 -4.7971502 0.9088660

C 0.2280718 -2.8216591 -0.5484228

C 0.0485016 -5.6437464 -0.1964502

H 0.0607346 -5.2023912 1.9244214

C 0.1933518 -3.6608800 -1.7006665

C 0.1002876 -5.0396559 -1.4798177

H 0.0647341 -5.6959410 -2.3529716

C -0.1749946 3.3888697 0.7588322

C -0.0931057 4.7971676 0.9090358

C -0.2283010 2.8216830 -0.5482491

C -0.0491221 5.6437815 -0.1962920

H -0.0597709 5.2022822 1.9246131

C -0.1934611 3.6608647 -1.7005072

C -0.1008916 5.0396751 -1.4796552

H -0.0660013 5.6959922 -2.3528104

C 0.2412746 -3.0465208 -3.1104940

C -0.9848762 -2.1171931 -3.3095066

C 1.5566588 -2.2428995 -3.2856541

C 0.1982690 -4.1314980 -4.2052551

H -0.9484053 -1.6632630 -4.3166123

H -1.9218633 -2.6952776 -3.2231789

H -1.0102054 -1.3111763 -2.5622551

H 1.5789512 -1.7811695 -4.2895398

H 1.6488307 -1.4464002 -2.5320013

H 2.4303808 -2.9131937 -3.1964617

H 0.2383927 -3.6473385 -5.1964086

H 1.0570341 -4.8225248 -4.1337423

H -0.7319646 -4.7252679 -4.1597343

C -0.2403001 3.0464286 -3.1103381

C 0.9861556 2.1172922 -3.3083950

C -1.5554315 2.2426077 -3.2864611

C -0.1966451 4.1313638 -4.2051154

H 0.9504304 1.6631617 -4.3154379

H 1.9229713 2.6955814 -3.2215731

H 1.0111820 1.3114221 -2.5609726

H -1.5769111 1.7808865 -4.2903681

H -1.6480402 1.4460967 -2.5328785

H -2.4293227 2.9127657 -3.1979055

H -1.0556583 4.8221608 -4.1343764

H 0.7333886 4.7253868 -4.1588247

H -0.2357842 3.6471464 -5.1962800

H 0.0161567 1.0262650 5.5026583

H -0.0140952 -1.0264000 5.5026536

C 1.9410602 -0.3252957 3.5713214

H 2.5360846 -1.2306763 3.3660895

H 2.2568160 0.4530472 2.8615472

H 2.1496126 -0.0025928 4.6047473

C -1.9397794 0.3252226 3.5720397

H -2.5348631 1.2306071 3.3669909

H -2.2558171 -0.4531419 2.8624175

H -2.1479610 0.0025826 4.6055607

C 0.0525690 7.1739975 -0.0830491

C 0.0847785 7.6398712 1.3853381

C 1.3517625 7.6496801 -0.7794226

C -1.1714150 7.8210443 -0.7772306

H -0.8316355 7.3436538 1.9258946

H 0.9558038 7.2268974 1.9242139

H 0.1569252 8.7406996 1.4234203

H 1.3643742 7.3707088 -1.8473387

H 1.4387945 8.7493668 -0.7156354

H 2.2388343 7.2015444 -0.2984439

H -1.1088154 8.9223001 -0.7126741

H -1.2238098 7.5478415 -1.8453919

H -2.1103563 7.4972943 -0.2947203

C -0.0544318 -7.1738677 -0.0831291

C -0.0863875 -7.6396856 1.3852773

C -1.3544473 -7.6482795 -0.7788780

C 1.1685730 -7.8221236 -0.7778852

H 0.8305466 -7.3442955 1.9254063

H -0.9567965 -7.2259364 1.9245515

H -0.1595253 -8.7404470 1.4233864

H -1.3672295 -7.3693771 -1.8468111

H -1.4426197 -8.7478697 -0.7149547

H -2.2408275 -7.1991547 -0.2975476

H 1.1048288 -8.9233170 -0.7133894

H 1.2207924 -7.5489054 -1.8460498

H 2.1080717 -7.4994198 -0.2957579

**Cat7**_OC_2_H_4_.xyz

103

Energy = -3127.126421725

Ti -0.0017636 0.4413849 -0.2748253

C -1.5764531 4.5874865 -0.1092329

C -0.6369657 3.4343649 0.2940548

C 0.6745798 3.4395437 -0.5781368

C 1.5861222 4.6219788 -0.2031037

H -2.3764415 4.7289605 0.6364124

H 2.0148692 4.5224288 0.8070053

H 2.4130337 4.7224255 -0.9256424

H -2.0400273 4.4148645 -1.0934490

N -1.2576483 2.0879968 0.0896941

N 1.3068908 2.1047979 -0.3517798

Cl -0.4896784 0.1776613 -2.5589142

O -1.4831704 -0.6368040 0.1985847

O 1.5596004 -0.5940949 -0.2919212

C -2.5688286 1.9842640 0.2129641

H -3.1490089 2.9047850 0.3700682

C 2.6160713 2.0311070 -0.2210815

H 3.1952192 2.9651737 -0.2190374

C -3.3730189 0.7913274 0.1644082

C -4.7876895 0.9370220 0.1741351

C -2.8010546 -0.5186477 0.1325552

C -5.6334596 -0.1651325 0.1141293

H -5.1930482 1.9525265 0.2183152

C -3.6459930 -1.6673811 0.0508479

C -5.0276132 -1.4481723 0.0423167

H -5.6852472 -2.3181907 -0.0267855

C 3.4269873 0.8458329 -0.1103420

C 4.8328021 0.9983110 0.0366439

C 2.8704071 -0.4665757 -0.2015043

C 5.6833092 -0.1022602 0.0759153

H 5.2281476 2.0159585 0.1118718

C 3.7240964 -1.6118727 -0.2256263

C 5.0961201 -1.3883162 -0.0727019

H 5.7622431 -2.2546002 -0.0755687

C -3.0422113 -3.0793898 -0.0624094

C -2.1771193 -3.1632265 -1.3466937

C -2.1724650 -3.3866782 1.1817342

C -4.1339641 -4.1649516 -0.1516329

H -1.7070591 -4.1615802 -1.4180944

H -2.8074165 -3.0123123 -2.2409656

H -1.3873015 -2.3975604 -1.3565399

H -1.7478062 -4.4040151 1.1040119

H -1.3437300 -2.6691198 1.2631762

H -2.7782972 -3.3333000 2.1040508

H -3.6538054 -5.1555200 -0.2369643

H -4.7765566 -4.1783337 0.7468322

H -4.7782516 -4.0270999 -1.0379026

C 3.1400740 -3.0169385 -0.4653262

C 2.1039676 -3.3768885 0.6285770

C 2.4535588 -3.0423105 -1.8567916

C 4.2378107 -4.0997584 -0.4514925

H 1.7432735 -4.4102789 0.4762630

H 2.5569623 -3.3140918 1.6342382

H 1.2393087 -2.7000828 0.5847193

H 2.0040703 -4.0364673 -2.0356045

H 1.6595610 -2.2822321 -1.9281738

H 3.1953911 -2.8524167 -2.6530968

H 4.9949650 -3.9283451 -1.2369531

H 4.7539340 -4.1483604 0.5239998

H 3.7777667 -5.0856818 -0.6389462

H 1.0107611 5.5604287 -0.2432461

H -1.0116037 5.5323140 -0.1496609

C -0.2920090 3.5663005 1.7935399

H -1.1960795 3.3661375 2.3934885

H 0.4885536 2.8437345 2.0781564

H 0.0526281 4.5866961 2.0279985

C 0.3371521 3.5318638 -2.0819834

H 1.2541159 3.3539522 -2.6687608

H -0.4009010 2.7653352 -2.3651108

H -0.0444526 4.5354435 -2.3329666

C 7.2069918 0.0141792 0.2526124

C 7.6600234 1.4799963 0.3937390

C 7.6315468 -0.7546890 1.5282494

C 7.9169284 -0.6015249 -0.9785146

H 7.4055574 2.0722065 -0.5029536

H 7.1979287 1.9646061 1.2719408

H 8.7557650 1.5197075 0.5237025

H 7.3588917 -1.8225550 1.4662987

H 8.7260411 -0.6916278 1.6684905

H 7.1411885 -0.3284267 2.4211745

H 9.0140199 -0.5343906 -0.8626604

H 7.6550516 -1.6660615 -1.1072399

H 7.6315517 -0.0657271 -1.9009534

C -7.1677292 -0.0531880 0.1104653

C -7.6386735 1.4111580 0.2015175

C -7.7227519 -0.6656465 -1.1993212

C -7.7403656 -0.8278323 1.3230647

H -7.2860873 1.8940481 1.1301299

H -7.2811434 2.0070148 -0.6567983

H -8.7422253 1.4477978 0.2003897

H -7.4429664 -1.7287358 -1.2992432

H -8.8261074 -0.6024086 -1.2158010

H -7.3297733 -0.1256315 -2.0786551

H -8.8439448 -0.7673301 1.3314454

H -7.4601034 -1.8949510 1.2903635

H -7.3614895 -0.4043979 2.2699871

O 0.4093036 0.4930945 1.9450156

C -0.5991478 0.3093405 2.9713840

C 0.4595482 -0.6920010 2.7801861

H -1.6018129 0.1057141 2.5781819

H -0.5274053 1.0323815 3.7920119

H 0.2240923 -1.6236864 2.2551312

H 1.3237716 -0.7164356 3.4539418

**Cat7**.xyz

96

Energy = -2973.387912694

Ti 0.7992040 0.0088109 0.4659268

C 0.3475178 -1.6031402 4.6035488

C 0.7669355 -0.6783765 3.4481833

C -0.1468350 0.5987498 3.3647207

C 0.1087212 1.5417465 4.5564626

H 1.0804311 -2.4163918 4.7368242

H 1.0855593 2.0443731 4.4756625

H -0.6770830 2.3105639 4.6369439

H -0.6472611 -2.0507499 4.4482779

N 0.6234491 -1.3090325 2.1024347

N 0.1776322 1.2398482 2.0448136

Cl 3.0894856 -0.0335654 0.2423393

O 0.2483509 -1.4454756 -0.5532538

O 0.4243908 1.4815967 -0.6060004

C 0.4783342 -2.6137930 2.0092950

H 0.5025408 -3.2114271 2.9310984

C -0.1102115 2.5328775 1.9281998

H -0.4509716 3.0639605 2.8275857

C 0.2561217 -3.3837721 0.8116416

C 0.1273869 -4.7934313 0.9265908

C 0.1180095 -2.7668236 -0.4651433

C -0.1201321 -5.5907763 -0.1876750

H 0.2290444 -5.2345324 1.9226376

C -0.1480175 -3.5533351 -1.6232342

C -0.2513132 -4.9378833 -1.4411816

H -0.4442075 -5.5574991 -2.3202996

C -0.0535328 3.3491362 0.7513325

C -0.3187167 4.7435043 0.8609922

C 0.2234963 2.7893457 -0.5319486

C -0.2864186 5.5743386 -0.2535536

H -0.5428167 5.1436705 1.8543149

C 0.2711683 3.6159588 -1.6930344

C 0.0170577 4.9793650 -1.5098407

H 0.0558219 5.6313470 -2.3857456

C -0.3055523 -2.8915303 -3.0045253

C -1.4998480 -1.9039400 -2.9687269

C 0.9992948 -2.1420075 -3.3796001

C -0.5858321 -3.9319498 -4.1077509

H -1.6089239 -1.4096460 -3.9509298

H -2.4378543 -2.4434123 -2.7467224

H -1.3517319 -1.1272547 -2.2039529

H 0.8793252 -1.6537398 -4.3638548

H 1.2522916 -1.3716522 -2.6366429

H 1.8426128 -2.8521981 -3.4473262

H -0.6943516 -3.4129593 -5.0760441

H 0.2402137 -4.6583806 -4.2071303

H -1.5199118 -4.4903447 -3.9191760

C 0.6216392 3.0182402 -3.0674365

C 2.0463584 2.4073934 -3.0096589

C -0.4084947 1.9253712 -3.4491253

C 0.6073311 4.0902229 -4.1757373

H 2.2960934 1.9491197 -3.9842015

H 2.7916059 3.1951905 -2.7989938

H 2.1295823 1.6360382 -2.2287327

H -0.1597146 1.5063559 -4.4411205

H -0.4091601 1.1043230 -2.7183323

H -1.4257252 2.3532186 -3.5021785

H -0.3884348 4.5560976 -4.2833177

H 1.3465532 4.8885914 -3.9861180

H 0.8639526 3.6179484 -5.1400356

H 0.0853292 0.9694884 5.4972825

H 0.3270960 -1.0336620 5.5458647

C 2.2494402 -0.2908084 3.6362225

H 2.8809842 -1.1817579 3.4846454

H 2.5574518 0.4714866 2.9040143

H 2.4206260 0.0873304 4.6575076

C -1.6394026 0.2072403 3.3227683

H -2.2448140 1.1019931 3.0998580

H -1.8254549 -0.5435617 2.5359647

H -1.9733986 -0.1992373 4.2913494

C -0.5531748 7.0877966 -0.1839746

C -0.8690571 7.5515471 1.2510151

C 0.7001745 7.8499578 -0.6812111

C -1.7605016 7.4367051 -1.0891121

H -1.7700777 7.0528159 1.6496906

H -0.0278434 7.3501620 1.9376026

H -1.0553096 8.6397363 1.2570853

H 0.9519410 7.5803636 -1.7214768

H 0.5240412 8.9404359 -0.6468845

H 1.5741378 7.6185821 -0.0472294

H -1.9604669 8.5231300 -1.0589463

H -1.5739397 7.1561263 -2.1402348

H -2.6680217 6.9067040 -0.7500544

C -0.2563176 -7.1213782 -0.1160885

C -0.0850514 -7.6475351 1.3218301

C -1.6590350 -7.5346756 -0.6263325

C 0.8288684 -7.7723117 -1.0091857

H 0.9109849 -7.3997162 1.7292514

H -0.8511723 -7.2325167 2.0003581

H -0.1881301 -8.7466929 1.3297065

H -1.8223124 -7.2119487 -1.6692126

H -1.7710948 -8.6334678 -0.5902653

H -2.4498296 -7.0835808 -0.0014375

H 0.7413044 -8.8734574 -0.9767398

H 0.7305684 -7.4558884 -2.0620448

H 1.8391300 -7.4932178 -0.6616764

Cl^−^.xyz

1

Energy = -460.2027239630

Cl 0.0000000 0.0000000 0.0000000

OC_2_H_4_.xyz

7

Energy = -153.7100829398

C -0.6673609 -0.4828708 -0.0000010

C 0.0214875 0.8173243 -0.0000038

O 0.7675324 -0.4104776 -0.0000010

H -1.1196677 -0.8586404 0.9297659

H -1.1196301 -0.8586774 -0.9297673

H 0.0784557 1.4026091 0.9297762

H 0.0784160 1.4026366 -0.9297692

**Cat7**_EtOAc.xyz

110

Energy = -3280.968658679

Ti -0.0245640 0.4772502 -0.4943220

C -1.5916936 4.6229827 -0.1160235

C -0.6300724 3.4572353 0.1815358

C 0.6183113 3.4875226 -0.7835440

C 1.5457128 4.6723137 -0.4614908

H -2.3420063 4.7346311 0.6844819

H 2.0149948 4.5904284 0.5320981

H 2.3436963 4.7562757 -1.2182652

H -2.1179607 4.4874463 -1.0742400

N -1.2648271 2.1171125 -0.0135585

N 1.2675547 2.1567695 -0.6074823

Cl -0.7340407 0.2897666 -2.7344523

O -1.4794925 -0.6090049 0.0401401

O 1.5410465 -0.5487193 -0.7301207

C -2.5673525 2.0107621 0.1650412

H -3.1418220 2.9275848 0.3611678

C 2.5717568 2.0820359 -0.4474811

H 3.1541107 3.0140119 -0.4269901

C -3.3690613 0.8153525 0.1465777

C -4.7806788 0.9554116 0.2450745

C -2.7938934 -0.4918012 0.0683459

C -5.6244430 -0.1495356 0.2355787

H -5.1861352 1.9690980 0.3197298

C -3.6396957 -1.6440194 0.0416384

C -5.0190446 -1.4303348 0.1242044

H -5.6767149 -2.3027408 0.0972030

C 3.3638899 0.8913900 -0.2668308

C 4.7360139 1.0402259 0.0714509

C 2.8133251 -0.4202454 -0.4105386

C 5.5567754 -0.0618460 0.2942581

H 5.1271410 2.0575747 0.1685422

C 3.6366762 -1.5702267 -0.1984026

C 4.9730104 -1.3501970 0.1515468

H 5.6089615 -2.2208450 0.3298215

C -3.0379353 -3.0525319 -0.1167182

C -2.2665967 -3.1291911 -1.4600839

C -2.0787748 -3.3598547 1.0602807

C -4.1280808 -4.1432636 -0.1300096

H -1.7943369 -4.1234915 -1.5655093

H -2.9612635 -2.9837906 -2.3063290

H -1.4862918 -2.3564214 -1.5255262

H -1.6657216 -4.3793546 0.9539419

H -1.2403367 -2.6490250 1.0787284

H -2.6172696 -3.3065544 2.0238141

H -3.6506712 -5.1315014 -0.2506933

H -4.7049713 -4.1606180 0.8120074

H -4.8346488 -4.0077601 -0.9678440

C 3.0377379 -2.9831859 -0.3209293

C 1.9126741 -3.1580956 0.7321015

C 2.4619119 -3.1897477 -1.7457886

C 4.0956524 -4.0763767 -0.0709307

H 1.4617930 -4.1628109 0.6412290

H 2.3198744 -3.0582055 1.7547088

H 1.1195400 -2.4097223 0.5919081

H 2.0258171 -4.2021712 -1.8286038

H 1.6797698 -2.4492705 -1.9715213

H 3.2640590 -3.0980435 -2.4999424

H 4.9244746 -4.0184752 -0.7986589

H 4.5215963 -4.0123975 0.9463469

H 3.6244134 -5.0693875 -0.1748007

H 0.9714093 5.6115052 -0.4944742

H -1.0294359 5.5691750 -0.1567432

C -0.1751519 3.5402067 1.6553580

H -1.0266977 3.3015966 2.3149683

H 0.6360131 2.8225501 1.8527632

H 0.1737002 4.5561054 1.9027723

C 0.1748616 3.5966773 -2.2575545

H 1.0495360 3.4350815 -2.9100476

H -0.5754628 2.8280058 -2.4992822

H -0.2306572 4.6006733 -2.4659256

C 7.0402652 0.0561911 0.6843734

C 7.4963214 1.5250222 0.7770348

C 7.2624870 -0.6124751 2.0635191

C 7.9088471 -0.6603890 -0.3789339

H 7.3801604 2.0479963 -0.1886275

H 6.9255767 2.0797131 1.5427320

H 8.5635704 1.5664305 1.0567995

H 6.9671975 -1.6758209 2.0510451

H 8.3282958 -0.5588767 2.3506269

H 6.6693292 -0.1019248 2.8428224

H 8.9788711 -0.5908694 -0.1112916

H 7.6491472 -1.7300417 -0.4602541

H 7.7681624 -0.1982867 -1.3718568

C -7.1561885 -0.0438696 0.3280375

C -7.6263308 1.4186893 0.4464620

C -7.7896314 -0.6603751 -0.9437615

C -7.6487273 -0.8189775 1.5749740

H -7.2186995 1.9040296 1.3509223

H -7.3245992 2.0146634 -0.4329390

H -8.7279941 1.4512192 0.5134931

H -7.5130251 -1.7227241 -1.0592924

H -8.8921109 -0.6007865 -0.8918794

H -7.4537441 -0.1206064 -1.8466122

H -8.7499879 -0.7640548 1.6518294

H -7.3655359 -1.8847805 1.5266554

H -7.2138673 -0.3917214 2.4957634

C 0.3222459 0.0652818 2.7007008

O 0.6514615 0.3859435 1.5526026

O 1.2424743 -0.1446978 3.6381980

C -1.0894216 -0.1361794 3.1653458

H -1.1533904 -0.0883813 4.2612767

H -1.7404799 0.6150245 2.6946475

H -1.4316636 -1.1272204 2.8226125

C 2.6450358 -0.0037675 3.2293739

C 3.5051325 -0.4776413 4.3818867

H 2.8148226 1.0563509 2.9780820

H 2.7977770 -0.5956384 2.3131235

H 4.5671600 -0.3734521 4.1032638

H 3.3221714 0.1198202 5.2906621

H 3.3091823 -1.5393592 4.6085778

**Cat7**_THF.xyz

109

Energy = -3205.757078677

Ti 0.0012638 0.3992170 -0.1881202

C -1.5843862 4.5447004 -0.0262214

C -0.6797510 3.3736854 0.4070485

C 0.6911665 3.4075148 -0.3643597

C 1.5808580 4.5574465 0.1466616

H -2.4306714 4.6726329 0.6687984

H 1.9711073 4.3653839 1.1591410

H 2.4337023 4.7286837 -0.5307033

H -1.9831171 4.4014363 -1.0429674

N -1.2877447 2.0376102 0.1167461

N 1.3074286 2.0579496 -0.1678127

Cl -0.2360285 0.2444223 -2.5282093

O -1.5253866 -0.6698277 0.0622729

O 1.5621066 -0.6394538 -0.1399620

C -2.6055689 1.9409814 0.1610348

H -3.1875772 2.8622893 0.3061539

C 2.6255698 1.9783913 -0.1246156

H 3.2039913 2.9128633 -0.1451977

C -3.4124727 0.7543393 0.0655443

C -4.8279215 0.8952542 0.0683361

C -2.8385518 -0.5534125 0.0094258

C -5.6687328 -0.2098784 -0.0044162

H -5.2376364 1.9082760 0.1263715

C -3.6776295 -1.7056811 -0.0813483

C -5.0592374 -1.4920442 -0.0858356

H -5.7145798 -2.3636683 -0.1584534

C 3.4409813 0.7927417 -0.0760610

C 4.8540398 0.9397584 -0.0093570

C 2.8758173 -0.5188797 -0.1204316

C 5.7001064 -0.1646092 0.0009442

H 5.2582952 1.9556842 0.0318592

C 3.7202509 -1.6700597 -0.1452086

C 5.0999073 -1.4513910 -0.0777099

H 5.7603931 -2.3219994 -0.0897320

C -3.0591424 -3.1098501 -0.2072454

C -2.2199289 -3.1774244 -1.5101495

C -2.1578361 -3.4129714 1.0170707

C -4.1411237 -4.2063798 -0.2770651

H -1.7277233 -4.1644187 -1.5898053

H -2.8737658 -3.0454026 -2.3906875

H -1.4475618 -2.3938616 -1.5372424

H -1.7430853 -4.4339919 0.9326100

H -1.3211676 -2.7020736 1.0778627

H -2.7424580 -3.3550538 1.9529292

H -3.6534779 -5.1924337 -0.3717717

H -4.7664149 -4.2253046 0.6335432

H -4.8037232 -4.0749232 -1.1507345

C 3.1103008 -3.0779866 -0.2748212

C 2.1566555 -3.3643812 0.9131213

C 2.3270096 -3.1695651 -1.6110628

C 4.1971546 -4.1717059 -0.2812046

H 1.7433439 -4.3853868 0.8229049

H 2.7013555 -3.2980080 1.8721575

H 1.3191707 -2.6521526 0.9302460

H 1.8496494 -4.1628949 -1.6988938

H 1.5445228 -2.3978657 -1.6761302

H 3.0154154 -3.0424043 -2.4657543

H 4.8982490 -4.0494491 -1.1256870

H 4.7804424 -4.1763224 0.6569495

H 3.7170768 -5.1605544 -0.3841267

H 1.0018674 5.4943457 0.1699981

H -1.0127808 5.4861972 -0.0045922

C -0.4516837 3.4566331 1.9320819

H -1.4013465 3.2490898 2.4538709

H 0.2916050 2.7115818 2.2507202

H -0.1137317 4.4633783 2.2266704

C 0.4650548 3.5864562 -1.8815472

H 1.4215769 3.4320342 -2.4089549

H -0.2536071 2.8422344 -2.2586654

H 0.1087646 4.6058220 -2.1042692

C 7.2320383 -0.0518935 0.0854038

C 7.6986413 1.4148875 0.1565185

C 7.7270672 -0.7856070 1.3563711

C 7.8660215 -0.7072843 -1.1663809

H 7.3922196 1.9826014 -0.7398076

H 7.2934710 1.9270971 1.0471299

H 8.8003879 1.4519450 0.2183962

H 7.4466316 -1.8531127 1.3421320

H 8.8281426 -0.7250317 1.4308267

H 7.2914164 -0.3302422 2.2631805

H 8.9682260 -0.6429246 -1.1167328

H 7.5929128 -1.7736332 -1.2480257

H 7.5290758 -0.1972365 -2.0859766

C -7.2034888 -0.1038741 -0.0042829

C -7.6801573 1.3583488 0.0906140

C -7.7604102 -0.7163389 -1.3131997

C -7.7692590 -0.8827157 1.2089779

H -7.3295945 1.8402577 1.0204846

H -7.3247703 1.9577646 -0.7661101

H -8.7838517 1.3906468 0.0897133

H -7.4770143 -1.7781916 -1.4159065

H -8.8640496 -0.6573691 -1.3258218

H -7.3725527 -0.1733364 -2.1929755

H -8.8730630 -0.8267409 1.2210479

H -7.4846257 -1.9486037 1.1738356

H -7.3889845 -0.4590808 2.1552582

O 0.1498647 0.3103041 2.0480485

C 1.3389011 0.0063429 2.8136910

H 2.1845257 0.5027869 2.3177605

H 1.5044325 -1.0866342 2.8028110

C -0.9815088 0.0258579 2.9051407

H -1.8385467 0.5852199 2.5047829

H -1.2032383 -1.0550726 2.8514462

C -0.5296694 0.4580935 4.3122859

H -0.8668808 -0.2667054 5.0702083

H 1.5216238 -0.0678475 4.9952436

H -0.9507010 1.4409997 4.5766499

C 1.0209543 0.5322161 4.2189163

H 1.3651940 1.5741910 4.3243283

EtOAc.xyz

14

Energy = -307.5468604523

C -0.1509803 -0.0612828 -0.0024328

O 1.0566842 -0.2379941 -0.0014234

O -0.7158838 1.1673520 -0.0036041

C -1.2015526 -1.1467650 -0.0025673

H -1.8475279 -1.0426317 0.8858052

H -1.8453359 -1.0444842 -0.8927473

H -0.7148603 -2.1317777 -0.0009387

C 0.2031725 2.2955575 -0.0037078

C -0.6300445 3.5630572 -0.0046816

H 0.8500847 2.2188047 -0.8947616

H 0.8492689 2.2196260 0.8880030

H 0.0359166 4.4427598 -0.0049551

H -1.2716462 3.6110451 -0.9011262

H -1.2722415 3.6120278 0.8912810

THF.xyz

13

Energy = -232.3350812526

O -0.0001870 -1.2509991 -0.0037162

C 1.1681655 -0.4224141 -0.1606846

C -1.1678070 -0.4231156 0.1605733

C -0.7361476 0.9964572 -0.2230319

C 0.7347569 0.9969122 0.2225840

H 1.9753595 -0.8219104 0.4793759

H 1.5118958 -0.4637901 -1.2152612

H -1.9778325 -0.8224876 -0.4758838

H -1.5064923 -0.4652342 1.2168525

H -0.8048712 1.1377993 -1.3170094

H -1.3457061 1.7751168 0.2647597

H 1.3439323 1.7759479 -0.2650785

H 0.8031187 1.1386507 1.3165262

# References

[20] G. R. Fulmer, A. J. M. Miller, N. H. Sherden, H. E. Gottlieb, A. Nudelmann, B. M. Stoltz, J. E. Bercaw, K. I. Goldberg, *Organometallics* **2010**, *29*, 2176 – 2179.

[21] W. Chen, K. Chen, X. Wang, L. Yang, W. Chen, *RSC Adv.* **2024**, *14*, 16624 – 16628.

[22] A. Tlili, F. Monnier, M. Taillefer, *Chem. Commun.* **2012**, *48*, 6408 – 6410.

[23] S. V. Samuelsen, C. Santilli, M. S. G. Ahlquist, R. Madsen, *Chem. Sci.* **2019**, *10*, 1150 – 1157.

[24] Y. Wang, Y. Zhao, S. Zhu, X. Zhuo, J. Xu, X. Xie, R. Poli, *Angew. Chem.* **2022**, *132*, 6044 – 6050.

[25] G. A. Morris, H. Zhou, C. L. Stern, S. T. Nguyen, *Inorg. Chem.* **2001**, *40* 3222 – 3227.

[26] N. Maudoux, T. Roisnel, V. Dorcet, J.-F. Carpentier, Y. Sarazin, *Chem. Eur. J.* **2014**, *20*, 6131 – 6147.

[27] S. C. Gagleva, V. A. Tuskaev, A. S. Lyadov, R. U. Takazova, B. M. Bulychev, *Pet. Chem.* **2020**, *60*, 291 – 297.

[28] S. Hildebrandt, A. Gansäuer, *Angew. Chem. Int. Ed.* **2016**, *55*, 9719 – 9722.

[29] H. Sharma, N. Singh, D. O. Jang, *Tetrahedron* **2014**, *55*, 6623 ­– 6626.

[30] A. Thevenon, A. Cyriac, D. Myers, A. J. P. White, C. B. Durr, C. K. Williams, *J. Am. Chem. Soc.* **2018**, *140*, 6893 – 6903.

[31] W. Chen, H. Sun, J. Jiang, Y.-Q. Zhang, *ChemCatChem* **2024**, *16*, e202400829.

[32] TURBOMOLE V7.4, 2019, a development of University of Karlsruhe and Forschungszentrum Karlsruhe GmbH, 1989-2007, TURBOMOLE GmbH, since 2007; available from <http://www.turbomole.com>.

[33] J. Tao, J. P. Perdew, V. N. Staroverov, G. E. Scuseria, *Phys. Rev. Lett.* **2003**, *91*, 146401.

[34] S. Grimme, J. Antony, S. Ehrlich, H. Krieg, *J. Chem. Phys.* **2010**, *132*, 154104 – 154119.

[35] S. Grimme, S. Ehrlich, L. Goerigk, *J. Comput. Chem.* **2011**, *32*, 1456 – 1465.

[36] F. Weigend, M. Häser, H. Patzelt, R. Ahlrichs, *Chem. Phys. Lett.* **1998**, *294*, 143 – 152.

[37] F. Weigend, R. Ahlrichs, *Phys. Chem. Chem. Phys.* **2005**, *7*, 3297 – 3305.

[38] A. Klamt, G. Schürmann, *J. Chem. Soc. Perk. Trans.* *2* **1993**, 799 – 805.

[39] K. Eichkorn, F. Weigend, O. Treutler R. Ahlrichs, *Theor. Chem. Acc.* **1997**, *97*, 119 – 124.

[40] F. Weigend, *Phys. Chem. Chem. Phys.* **2006**, *8*, 1057-1065.

[41] P. Deglmann, K. May, F. Furche, R. Ahlrichs, *Chem. Phys. Lett.* **2004**, *384*, 103-107.

[42] S. Grimme, *Chem. Eur. J.* **2012**, *18*, 9955-9964.

[43] F. Eckert, A. Klamt, *AIChE Journal* **2002**, *48*, 369-385.

[44] F. Eckert, A. Klamt, COSMOtherm, Version C3.0, Release 16.01; COSMOlogic GmbH & Co. KG, Leverkusen, Germany 2015.

[45] Y. Zhao, D. G. Truhlar, *J. Phys. Chem. A* **2005**, *109*, 5656-5667.

[46] F. Weigend, F. Furche, R. Ahlrichs, *J. Chem. Phys.* **2003**, *119*, 12753-12762.

# NMR Spectra

^1^H-NMR of **S1**

^13^C-NMR of **S1**

^1^H-NMR of **S2**

^13^C-NMR of **S2**

^1^H-NMR of **S3**

^13^C-NMR of **S3**

^1^H-NMR of **S4**

^13^C-NMR of **S4**

^1^H-NMR of **S7**

^13^C-NMR of **S7**

^1^H-NMR of **S9**

^13^C-NMR of **S9**

^1^H-NMR of **S11**

^13^C-NMR of **S11**

^1^H-NMR of **S12**

^13^C-NMR of **S12**

^1^H-NMR of **S13**

^13^C-NMR of **S13**

^1^H-NMR of **P1**

^13^C-NMR of **P1**

^1^H-NMR of **P2**

^13^C NMR of **P2**

^1^H-NMR of **P3**

^13^C-NMR of **P3**

^1^H-NMR of **P4**

^13^C-NMR of **P4**

^1^H-NMR of **P5**

^13^C-NMR of **P5**

^1^H-NMR of **P7**

^13^C-NMR of **P7**

^1^H-NMR of **P8**

^13^C-NMR of **P8**

^1^H-NMR of **P9**

^13^C-NMR of **P9**

^1^H-NMR of **P11**

^13^C-NMR of **P11**

^1^H-NMR of **P12**

^13^C-NMR of **P12**

^1^H-NMR of **L3**

**
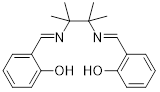

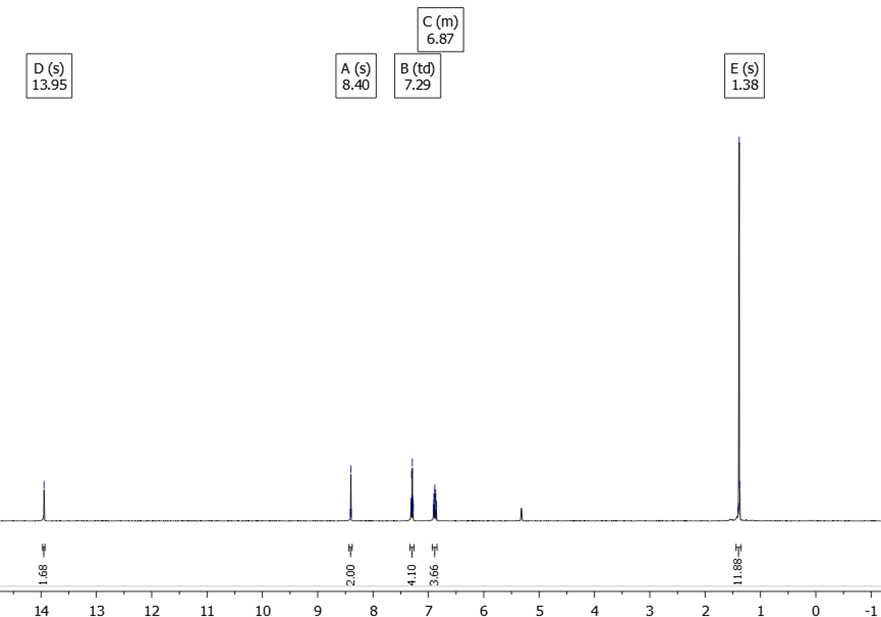
**

^13^C-NMR of **L3**

**
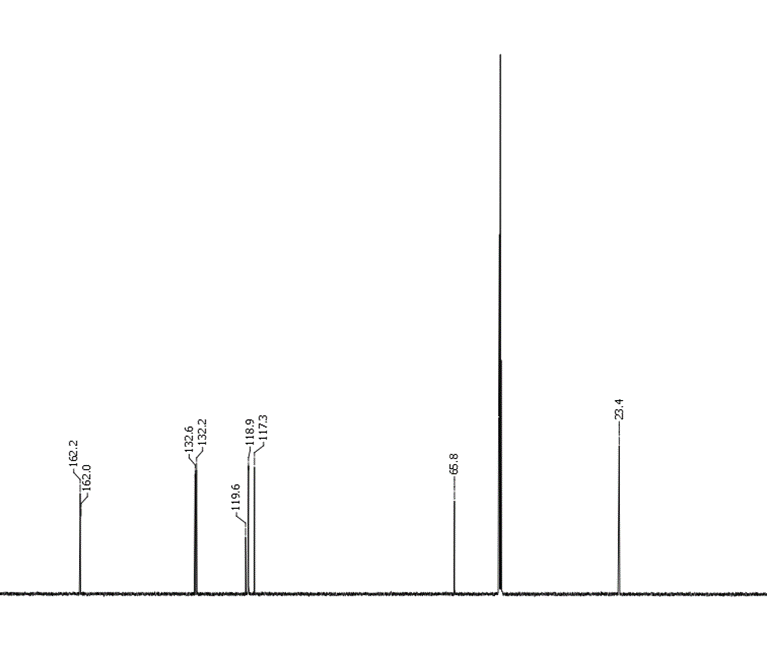
**

^1^H-NMR of **cat1**

^13^C-NMR of **cat1**

^1^H-NMR of **cat3**

**
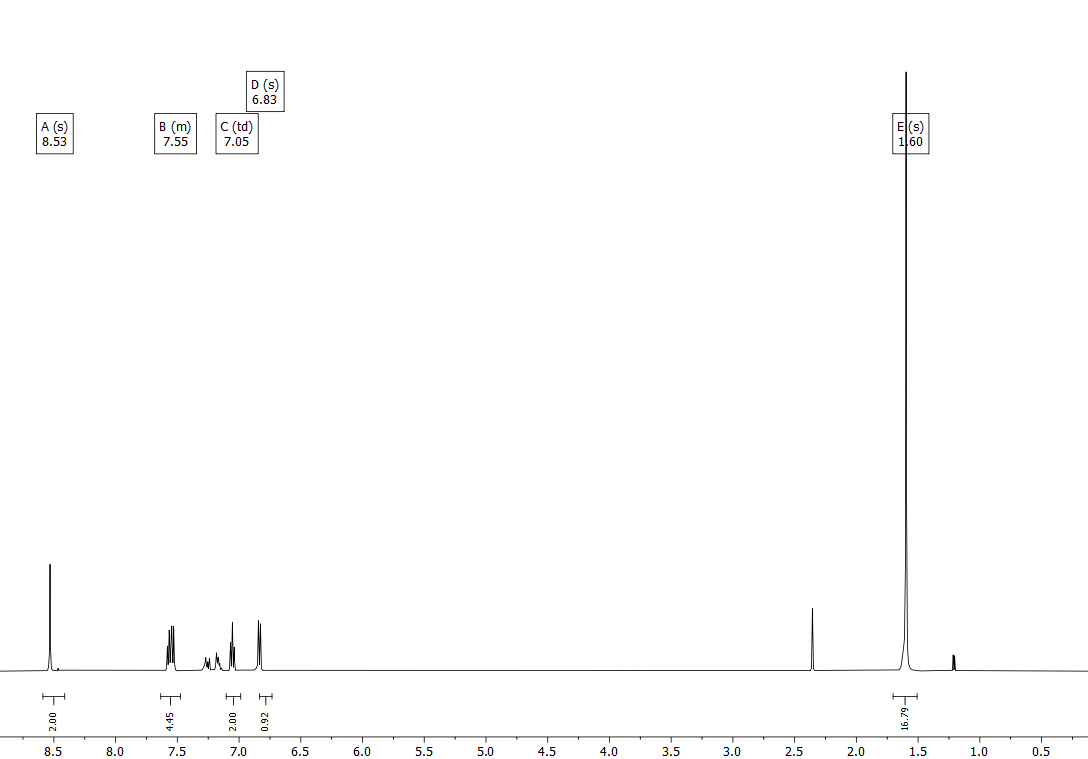
**

^13^C-NMR of **cat3**

**f**


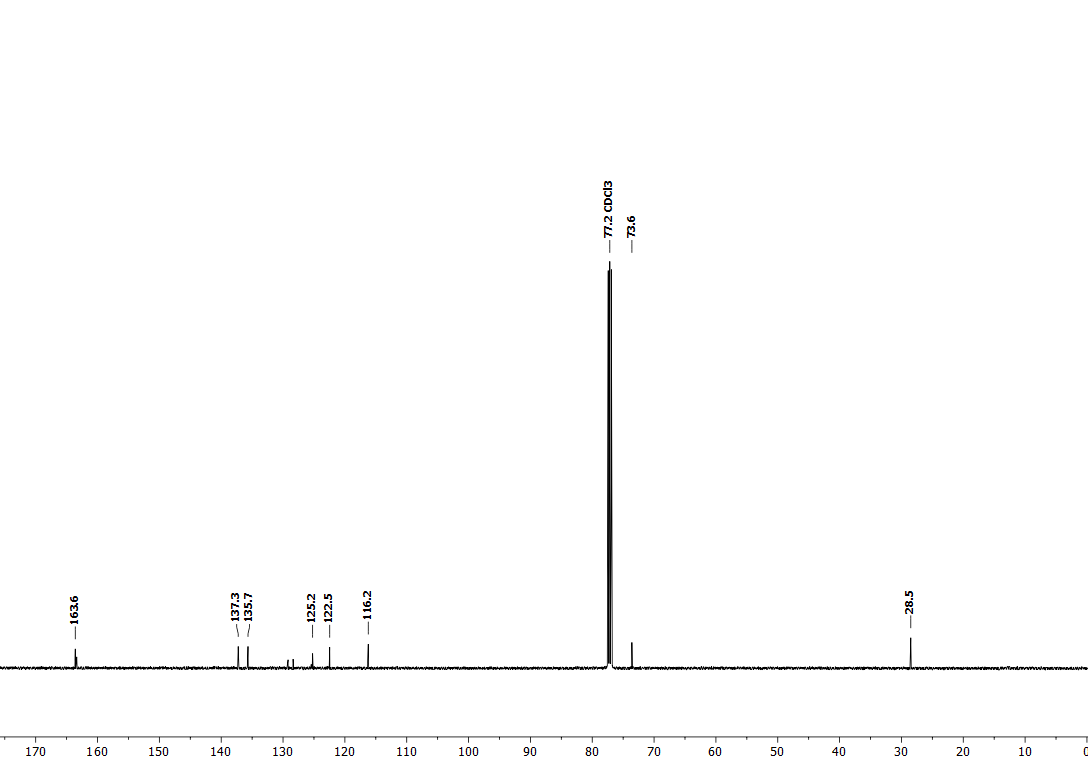


^1^H-NMR of **cat4**

^13^C-NMR of **cat4**

^1^H-NMR of **cat7**


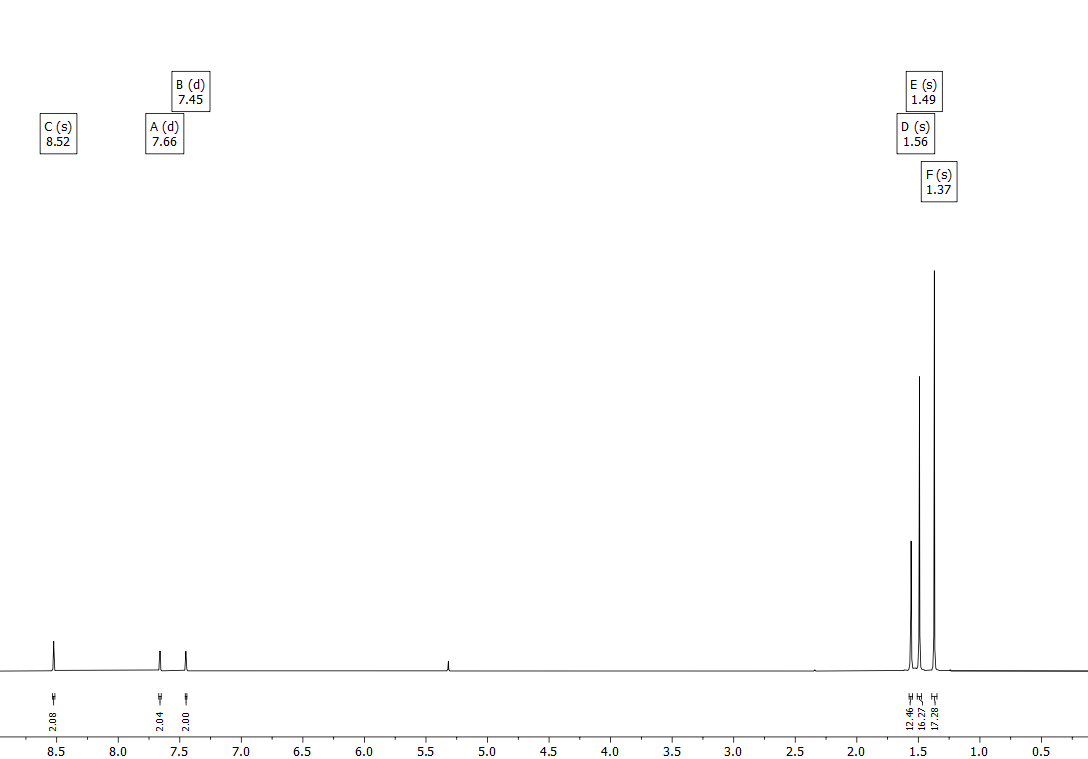


^13^C-NMR of **cat7**


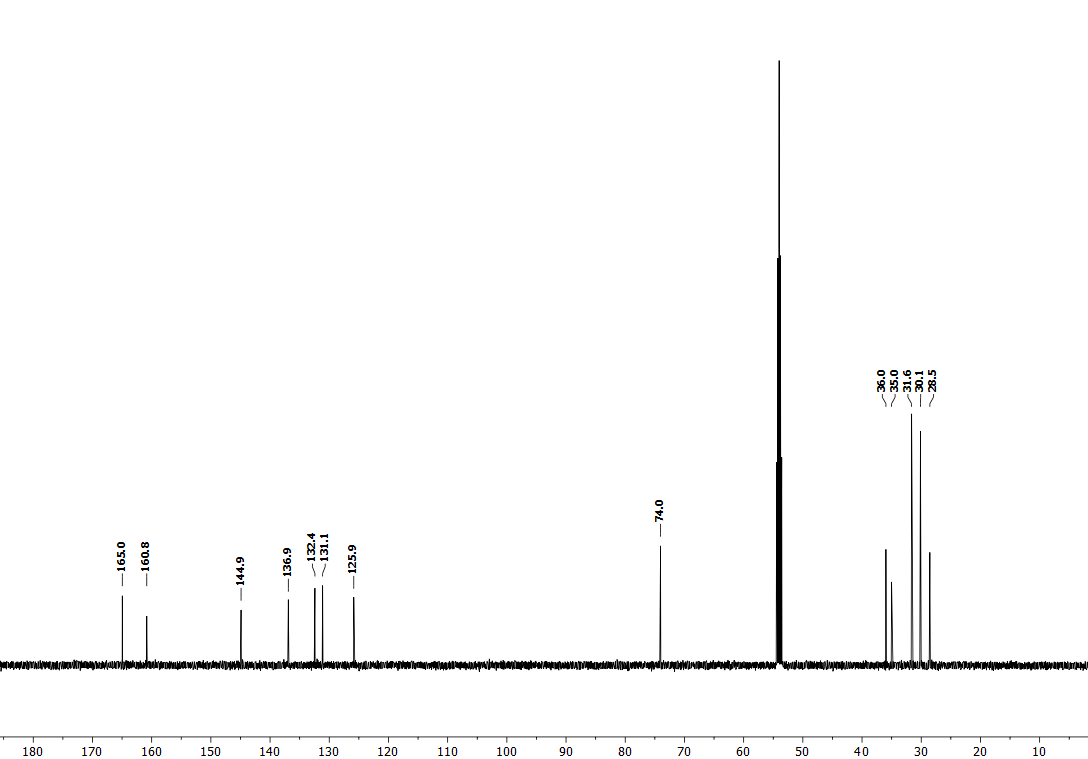

Supplement: Supplementary file 1 — Supporting Information [file ANIE-64-e202507673-s001.docx]
